# A refined *Saccharomyces cerevisiae* reference transcriptome from direct RNA sequencing, with a reusable pipeline for UTR annotation updates

**DOI:** 10.1093/femsyr/foag052

**Published:** 2026-09-23

**Authors:** Orlane Rossini, Alice Cleynen, Nikolay E Shirokikh

**Affiliations:** CNRS, University of Montpellier, Montpellier 34090, France; IRL FAMSI, CNRS International Lab, Australian National University, Canberra 2600, Australia; Australian Centre for RNA Therapeutics in Cancer, School of Human Sciences, University of Western Australia, Perth 6009, Australia

**Keywords:** UTR annotation, direct RNA sequencing, reference transcriptome, genome annotation, segmentation

## Abstract

Untranslated regions (UTRs) flanking the coding sequence govern mRNA translation, localization, and decay, making accurate UTR boundaries essential for quantitative RNA sequencing and the study of post-transcriptional control. Reference transcriptomes built from short-read sequencing have been invaluable to the yeast community, yet in a genome as gene-dense as that of *Saccharomyces cerevisiae*, short reads cannot be assigned to a single transcript of origin, leaving roughly one quarter of transcripts without a defined UTR. Here, we use direct RNA sequencing (DRS), in which native polyadenylated molecules are sequenced without fragmentation or amplification, to reduce this ambiguity and deliver two complementary resources. First, an updated *S. cerevisiae* S288C reference: change-point segmentation of per-gene DRS coverage defined boundaries for 5682 of the 6607 genes in the backbone model, and a merge-max rule retaining the longer UTR from each source ensures no gene loses existing annotation. The result adds previously absent UTRs to 1213 (5′) and 1220 (3′) of the genes that the earlier short-read annotation left unannotated, and extends 28.9% of 5′ and 19.4% of 3′ boundaries among comparable genes. Second, the complete, documented pipeline so that the transcriptome can be rebuilt from any DRS data. Validation on two independent datasets shows improved mapping rates, reduced soft-clipping, and metagene profiles consistent with genuine transcript signal.

## Teaser

Direct RNA sequencing reads each yeast transcript end to end, delivering a refined transcriptome reference that improves the accuracy of transcript quantification and any downstream analysis relying on UTR-inclusive sequencing.

## Introduction

Accurate gene expression measurement by RNA sequencing relies on a reference transcript model that correctly defines the boundaries of each expressed region of the DNA. Untranslated regions (UTRs), the stretches of mRNA upstream (5′ UTR) and downstream (3′ UTR) of the coding sequence, are not merely spacers: they are the primary sites of post-transcriptional control, harbouring upstream open reading frames that modulate ribosome recruitment and translational efficiency, binding sites for RNA-binding proteins, and sequence elements governing mRNA stability, localization, and decay (Ingolia et al. 2009, Archer et al. 2016, Hinnebusch et al. 2016, Hentze et al. 2018, Shirokikh and Preiss 2018, Janapala et al. 2019). When UTR boundaries are under-specified in the reference (either too short or absent altogether) reads originating in the untranslated portion of the transcript fail to map, quantification estimates are biased towards the coding region alone, and any downstream biological inference built on those estimates inherits this systematic error. The practical consequences compound in proportion to the fraction of a gene’s signal that falls in its UTRs, which for many yeast transcripts is substantial.

Much of the *S. cerevisiae* UTR annotation in routine use today traces back to foundational short-read transcriptome studies, most influentially that of Nagalakshmi et al. (2008), which mapped the transcribed regions of the yeast genome at nucleotide resolution and revealed a transcriptome considerably more extensive than the annotated coding sequences alone. These resources remain widely used and are well validated for coding-region quantification. Their UTR coverage, however, is bounded not by the care of any individual study but by an intrinsic property of short-read data: a short cDNA read is a fragment and carries no information about the full-length transcript from which it originated. In the compact *S. cerevisiae* genome, where adjacent genes are often separated by only a few hundred nucleotides (Goffeau et al. 1996), reads from neighbouring transcripts (whether through transcriptional read-through, alternative polyadenylation at a proximal site, or simple gene overlap) are difficult to distinguish from reads genuinely derived from the UTR of the focal gene (Tian and Manley 2017). Boundaries must therefore be inferred from aggregate coverage patterns, and only the most unambiguous UTRs, well separated from any neighbour, can be called with confidence. As a consequence a substantial fraction of genes carry no defined UTR (approximately one quarter of protein-coding genes in current short-read-derived annotations lack a 5′ or 3′ boundary call), and the boundaries that are annotated tend towards conservative, truncated estimates.

Direct RNA sequencing (DRS) by Oxford Nanopore Technologies (Garalde et al. 2018) addresses this limitation at the level of individual molecules. DRS reads native polyadenylated RNA without reverse transcription or fragmentation, so each read derives from one RNA molecule and is anchored at the 3′ poly-A tail. Sequencing then proceeds from that anchor towards the 5′ end. A read that spans the whole molecule carries its transcript of origin explicitly, which is the information a short cDNA fragment cannot carry, and the 3′ boundary is recovered on close to every read that maps.

This is a real advantage over short reads, but it is not an absolute one, and two limits matter for what follows. First, reads truncate. A read that stops before the transcription start site under-reports 5′ UTR length, and because the poly-A anchor sits at the 3′ end, truncation falls almost entirely on the 5′ boundary. We quantify that asymmetry and its consequences below. Second, DRS has a short blind region of ~14 nucleotides at the extreme 5′ terminus that is not captured in the read (Workman et al. 2019). Our pipeline applies no correction for it, so 5′ boundary calls should be read as short by about that amount.

Prior long-read studies in yeast have begun to exploit DRS advantages (Jenjaroenpun et al. 2018). Al Kadi et al. (2020) applied Oxford Nanopore sequencing of full-length cDNA to annotate transcript boundaries across the *S. cerevisiae* genome (the UNAGI resource), reporting improved UTR coordinates alongside novel transcripts and isoforms. The present work differs from this and related efforts in three respects. First, we use DRS of native RNA rather than cDNA sequencing, avoiding the reverse transcription and amplification steps that can introduce length-dependent bias and displace apparent 5′ ends (Garalde et al. 2018). Second, UTR boundary characterization relative to the community-standard Nagalakshmi et al. reference (Nagalakshmi et al. 2008) is the primary aim, allowing a focused and exhaustively validated treatment of UTR length rather than a general transcript reconstruction. Third, we produce an explicit merge against the Nagalakshmi et al. annotation at every gene boundary, retaining the longer UTR from either source, that is formally verified to be no more restrictive than the established reference at any locus. The result is a transcript FASTA that can serve as a drop-in replacement for the standard short-read reference in any existing yeast RNA-seq pipeline, without requiring users to evaluate whether the substitution introduces regressions.

Here, we present an updated *S. cerevisiae* transcript reference derived from DRS data generated in (Horvath et al. 2024) and extended by change-point segmentation of per-gene coverage profiles. We validate the reference against two independent datasets, an independent long-read DRS experiment (Fonzino et al. 2026) and the short-read Illumina data of (Al Kadi et al. 2020), across four complementary measures: read mapping rate, multi-mapping rate, antisense alignment rate, and soft-clip burden. We further validate boundary placement via metagene coverage profiles in a shared ORF-relative coordinate system, demonstrating that our extended boundaries capture genuine transcript signal rather than artefactual sequence inclusion. Together these constitute two complementary deliverables: an updated, ready-to-use *S. cerevisiae* S288C reference (transcript FASTA and GFF3 annotation) and a complete, reproducible pipeline for generating or extending such a reference from new DRS data. Both are provided as a community resource at https://github.com/Arnaroo/Yeast_Annotation [Zenodo 10.5281/zenodo.20828038].

## Materials and methods

### Data accession

Sequencing data used to build the transcriptome reference were generated in Horvath et al. (2024) and are described in full therein. Briefly, the dataset consists of long-read DRS libraries prepared from ribosome–disome–trisome (RDT) and higher polysome (PS) RNA fractions of *S. cerevisiae* (strain BY4741, an S288C background derivative) sampled under non-starved conditions (NS) and after 10 min of glucose starvation (S10). Libraries were sequenced on an Oxford Nanopore Technologies platform using the SQK-RNA002 Kit, which runs on R9.4.1 pore chemistry. All raw sequencing data are publicly accessible at the NCBI Sequence Read Archive (SRA) under BioProject PRJNA1022817; the eight individual run accessions used here, one per condition/replicate combination, are listed in Supplementary Table S1.

Long read validation data were generated in Fonzino et al. (2026) and are fully described therein. Briefly, total RNA from *S. cerevisiae* was used for Nanopore libraries preparation with the SQK-RNA002 Direct RNA Sequencing Kit, and was loaded on a single R9.4.1 PromethIon flow cell for sequencing. Raw sequencing data are publicly accessible at the NCBI SRA under experiment accession SRX28193073. The single run analysed here is SRR32918129.

Short read validation data were generated in Al Kadi et al. (2020) and are described in full therein. Briefly, *S. cerevisiae* BY4741 strain total RNA was used to generated full-length cDNA using the SMARTer Ultra Low RNA Kit for Illumina Sequencing and sequenced with HiSeq 2500 × 75 nt. The datasets extracted from this study are publicly accessible at DDBJ database (BioProject PRJDB8048) under the accession codes DRR170478 and DRR170479.

### In-house DRS reference

Two custom transcript (mRNA) references for *S. cerevisiae* were constructed to compare the effect of UTR definition on downstream transcript-level analyses while holding the genome assembly and gene models constant. Both references were built from the SGD S288C genome assembly R64-4-1 [release 2023-08-23; (Cherry et al. 2012)] and a shared canonical single-isoform gene model per locus (gene, CDS and intron features for 6607 genes from the SGD R64-4-1 annotation, reduced to one transcript per gene). The two references differ only in the source of the 5′ and 3′ UTR lengths used to extend each transcript. We describe below how those UTR lengths were obtained for the in-house DRS reference.

Each of the four fraction-level conditions (NS_PS, NS_RDT, S10_PS, and S10_RDT) was sequenced in two biological replicates. Reads were mapped to the *S. cerevisiae* ORF ± 1000 nt reference [SGD S288C assembly R64-4-1] using minimap 2.31 (Li 2018). Before segmentation, per-gene, per-position read counts were summed across replicates within a condition to produce a single combined coverage profile; conditions were not segmented per replicate and then merged. NS_RDT is an exception: one of its two replicates showed a primary mapping rate of 39.5%, against 82%–96% for every other replicate across all conditions, and was excluded as a likely library-quality problem; NS_RDT therefore uses its single remaining replicate alone. Two further, coarser profiles, NS and S10, were formed by additionally pooling the two RNA fractions (PS and RDT) within each physiological state, so that six coverage profiles in total (NS_PS, NS_RDT, S10_PS, S10_RDT, and NS, S10) enter the per-gene maximum described below. In each resulting condition, Genes with 20 or fewer reads at their position of maximum coverage were excluded from further analysis, the implemented test being strictly >20 (a gene that fails it in all six receives no data-driven UTR estimate at that end, falling back on the Nagalakshmi et al. boundary where one exists). Breakpoint detection was performed gene-by-gene using the Segmentor3IsBack algorithm (Cleynen et al. 2014) with a negative binomial model, which accounts for the overdispersion characteristic of sequencing data [Segmentor(data, model = 3)]. Because the data violate the independence assumptions of the base model, the initial segmentation typically yields a large number of breakpoints. We first remove noisy, closely spaced breakpoints by examining breakpoints within a 40 nt window. For each cluster of nearby breakpoints, we compute the mean coverage on the left and right sides and retain only the breakpoint with the largest absolute difference between these means. We then filter the remaining breakpoints by effect size, keeping only those where the mean coverage difference across the breakpoint exceeds 10% of the gene’s maximum transcript count.

Expressed regions were defined within the 1000 nt-ORF-1000 nt window: a segment was called expressed if its mean transcript count exceeded the lower of (i) 5% of the maximum count or (ii) 100 reads. UTR boundaries were extended beyond the 1000 nt window sequentially if the flanking segment met a stricter threshold of 10% of the maximum (or 100 reads), continuing until coverage dropped below threshold. Final UTR boundaries per gene were taken as the maximum UTR length observed across all 6 conditions and RNA fractions [PS/RDT]. The overall process is illustrated in Fig. 1a.

**Figure 1:**
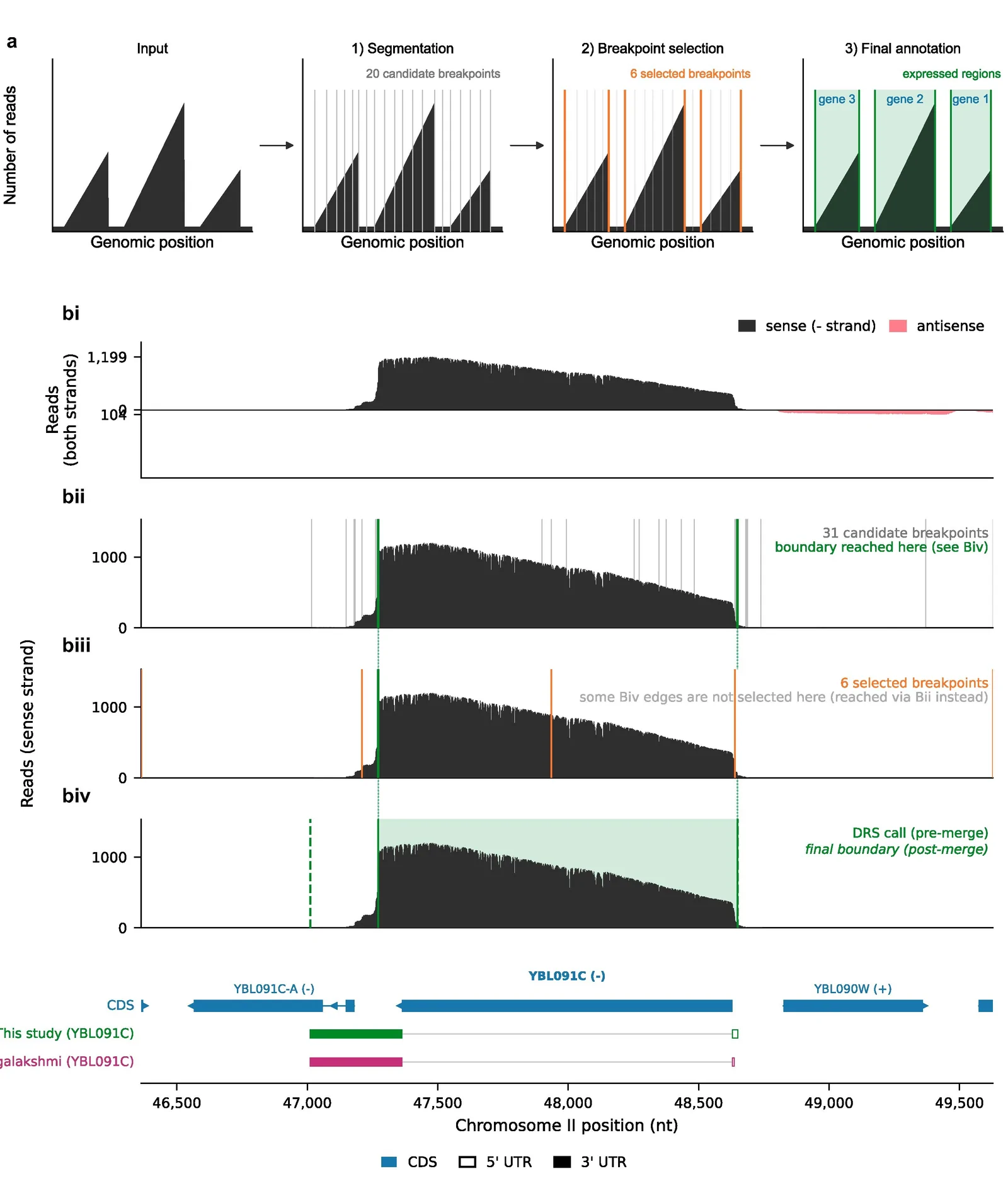
The DRS pipeline for calling UTR boundaries, and the same four steps applied to one locus. (a) Schematic of the four steps, drawn left to right for a window holding three adjacent genes. Panel a is a sketch. No axis in it carries a number and the shapes are illustrative rather than measured. Input, the per nucleotide read count across a genomic window. Step 1, segmentation, in which change point detection places a breakpoint wherever the level of coverage shifts, deliberately over segmenting the locus. Step 2, breakpoint selection, which keeps only the breakpoints supported by enough reads and by a large enough change in level. Step 3, final annotation, in which the surviving breakpoints define the expressed region and the 5′ and 3′ UTRs of each gene. (b) The same four steps on a real locus, YBL091C on chromosome II, positions 46 363–49 628. Read depth is on a logarithmic axis, sense reads above the axis and antisense reads below, so the strand of every signal is visible. (bi) Raw coverage. (bii) The 31 candidate breakpoints returned by segmentation. (biii) The 6 breakpoints that survive selection. (biv) The expressed region the method retains, shaded. Beneath the tracks, three rows share the same coordinates: the coding sequences with strand arrows, the annotation from this study, and the Nagalakshmi et al. (2008) annotation. At this locus the 5′ UTR is extended from 8 nt to 21 nt and the 3′ UTR is unchanged at 352 nt. Coverage in panel b is real construction-stage DRS coverage, summed over the four base real conditions (NS_PS, NS_RDT, S10_PS, and S10_RDT), the same data type the released boundaries were called from, not an independent library. The dashed line in panel biv marks the actual released, post-merge boundary, verified to reproduce the deposited final_utr.tsv exactly; the shaded solid-line span is this study’s own pre-merge DRS-only call on the same real data. Per locus annotations are merged with the Nagalakshmi et al. (2008) short read reference by retaining the longer boundary at each end, as described in Methods.

For each annotated gene, UTR coordinates from our segmentation were compared to the corresponding coordinates in Nagalakshmi et al. (2008). At each boundary (5′ and 3′ independently), the longer UTR was retained.

For the comparison reference, 5′ and 3′ UTR lengths were taken directly from (Nagalakshmi et al. 2008) genome-wide transcript boundary annotation, in place of the in-house DRS estimates.

### GFF3 annotation

For each reference a GFF3 annotation was generated by adding, for every transcript, a 5′ UTR and a 3′ UTR feature immediately flanking the gene model on the appropriate strand: on the plus strand the 5′ UTR spans (gene start − 5′ UTR length) to (gene start – 1) and the 3′ UTR spans (gene end + 1) to (gene end + 3′ UTR length); the assignment is reversed on the minus strand. UTR intervals were clamped to chromosome bounds, and a gene with a zero or sub-2 nt UTR interval received no UTR on that side. Spliced transcript sequences (coding sequence plus UTRs, introns removed) were then extracted from the genome with gffread [v0.12, (Pertea and Pertea 2020)], with locus clustering and identical-transcript collapsing enabled and the coding extent recorded in each FASTA description (CDS = start-end). Each reference comprises 6767 transcript sequences. Comparisons between annotations were made per boundary rather than per gene: a gene enters a comparison at a given end only where both annotations report a non-zero length at that end, and the 20 nt agreement tolerance used throughout is inclusive.

### Threshold selection and sensitivity

Four thresholds govern the segmentation: the coverage floor for admitting a gene (a maximum per-position coverage strictly >20 reads), the window within which neighbouring breakpoints are clustered before the strongest is retained (40 nt), the effect size a breakpoint must show to survive filtering (10% of the gene maximum), and the floor at which a segment is called expressed (the lower of 5% of the gene maximum and 100 reads).

To assess the sensitivity of the released annotation to these choices, we re-ran the full boundary-calling procedure over a grid in which each of the four thresholds took five values spanning well beyond the released value on each side: coverage floor 5, 10, 20, 40, and 80 reads; clustering window 10, 20, 40, 80, and 160 nt; effect size 2.5%, 5%, 10%, 20%, and 35% of the gene maximum; and expression floor 1%, 2.5%, 5%, 10%, and 20%. All 625 combinations were run on all six real construction-stage conditions (NS_PS, NS_RDT, S10_PS, S10_RDT, and NS, S10) and reduced to the same per-gene maximum used to build the released annotation, so that the sensitivity analysis directly evaluates the released calls rather than approximating them on an unrelated library.

For each parameter combination, we quantified the proportion of genes for which the resulting 5′ and 3′ boundaries were unchanged relative to the released annotation. The complete per-combination parameter-wise summaries are reported in Supplementary Fig. S1.

### Validation of the transcriptome

#### Comparison of Nagalakshmi et al. and long-read UTR annotations

To validate our UTR annotation, we compared the UTR lengths derived from the Nagalakshmi et al. (2008) short-read sequencing based annotation with those in our final merged table, on the subset of genes present in both and with non-missing Nagalakshmi estimates. Because our final UTR lengths are defined as the longest UTR observed across all available evidence including the Nagalakshmi annotation itself, a necessary property of a correct merge is that the Nagalakshmi length should never exceed the final length for any given gene. We therefore used this as a primary sanity check, computing the per-gene difference (final − Nagalakshmi) for both 5′ and 3′ UTRs and flagging any gene where this difference was negative.

Beyond this binary check, we examined the overall relationship between the two annotations by computing Spearman and Pearson correlations between Nagalakshmi and final UTR lengths, restricted to genes where both sources reported a non-zero length (this yielded 4792 and 4817 comparable genes for 5′ and 3′, respectively). We also compared the distributions of UTR lengths across both annotations and quantified the extent to which the long-read data extended UTR boundaries relative to the Nagalakshmi baseline. All analyses were performed in R using the dplyr, tidyr, and ggplot2 packages and Python using the pysam library.

To benchmark the final annotation against a fully independent transcript-boundary dataset, we additionally compared it with the transcript-isoform sequencing (TIF-seq) map (Gene Expression Omnibus accession GSE39128) (Pelechano et al. 2013), which captures the 5′ and 3′ ends of individual transcripts simultaneously. For each ORF, we identified the major covering transcript isoform (mTIF), defined as the most highly read-supported isoform (summed counts across all reported conditions) whose 5′ end lay upstream of the start codon and whose 3′ end lay downstream of the stop codon, and converted its boundaries to per-gene 5′ and 3′ UTR lengths using the ORF coordinates from the gene-model backbone. Because TIF-seq is independent of our reference and plays no part in the longest-UTR merge, boundary differences were treated as signed in both directions and concordance was summarized by Spearman rank correlation and by the fraction of genes agreeing within 20 nt (Supplementary Fig. S2).

#### Comparision of UNAGI annotation

A second independent benchmark was drawn from the UNAGI annotation of *S. cerevisiae* (Al Kadi et al. 2020), built from nanopore full-length cDNA. The two supplementary tables of that study report, for each gene, the transcript start or end alongside the coordinate of the first or stop codon, so a UTR length is the difference of two columns of a single table and requires no projection between coordinate systems. Genes were matched to the gene-model backbone on chromosome, strand, and the genomic coordinate of the start or stop codon, rather than through an external accession map. The offset between the two coordinate conventions was measured rather than assumed, and is that of a 0 based half open interval against a 1 based inclusive one; it resolved every joinable row on each strand. Rows matching more than one gene were discarded. The comparison then followed the rule used for TIF-seq, per boundary, both tails signed, and concordance assessed at tolerances of 20, 50, and 100 nt, a gene counting as concordant when the absolute difference falls below the tolerance.

### Read alignment of validation data

#### Long read DRS

DRS reads were aligned to each reference using minimap2 v2.31. For the genome reference, the splice-aware preset -ax splice was used with the additional flags -uf (forward-strand-only, appropriate for DRS data derived from polyadenylated mRNA) and -k14. For all four transcriptome references (ORF only, ORF ± 1000 nt, our annotation, Nagalakshmi), the preset -ax map-ont -k14 -N10 was used. This preset is optimized for Oxford Nanopore reads aligned to a linear (non-spliced) reference. The -N10 flag retains up to 10 secondary alignments per read, enabling quantification of multi-mapping behaviour. All BAMs were coordinate-sorted and indexed with samtools v1.23 (Danecek et al. 2021).

#### Short reads Illumina

For short-read alignment to transcriptome references, STAR v2.7.11b (Dobin et al. 2013) was used. Because STAR requires a pre-built genome index, each transcriptome FASTA was indexed independently using –runMode genomeGenerate with –genomeSAindexNbases 11 and –genomeChrBinNbits 11, parameters scaled to the small size of the yeast transcriptome (~14 Mb for the ORF-only reference, larger for UTR-containing references; the recommended value follows the formula min[14, log2(GenomeLength)/2 − 1)]. Reads were aligned with –outSAMattributes NH HI AS NM to retain the NH (number of hits) tag required for multi-mapping quantification. Output was written directly as coordinate-sorted BAM. The two short-read samples were aligned separately and then merged with samtools merge.

Short-read alignment to the genome reference used bwa-mem2 v2.2.1 (Li and Durbin 2009, Vasimuddin et al. 2019) with default parameters (bwa-mem2 mem). Each sample was aligned separately, sorted, and then merged with samtools merge.

### Alignment quality metrics

The following metrics were computed for every BAM file using samtools v1.23 and custom shell scripts. Unless stated otherwise, secondary (SAM flag 0 × 100) and supplementary (0 × 800) alignments were excluded from all counts using samtools view -F 0 × 900.

#### Mapping rate

For DRS BAMs and the genome-aligned short-read BAM, the mapping rate was computed from samtools flagstat as the fraction of primary reads with at least one alignment. For short-read transcriptome BAMs, STAR does not write unmapped reads to the BAM output by default, causing flagstat to report a spurious 100% mapping rate. The true denominator was therefore computed directly from the input FASTQ files: reads were counted as the number of lines divisible by 4 (i.e. awk ‘NR%4==1’ | wc -l), and the total across both samples (DRR170478 and DRR170479) was used as the denominator for all short-read transcriptome mapping rates. The mapping rate is reported as the number of primary mapped reads in the BAM divided by this FASTQ-derived total.

#### Multi-mapping rate

The metric definition differed by aligner, because minimap2 and STAR use different conventions for recording multiple alignments. For STAR short-read BAMs, the NH auxiliary tag (number of loci the read maps to) is written for every primary alignment. Reads with NH ≥ 2 were counted as multi-mappers using samtools view piped to an awk filter (NH:i: tag parser). The multi-mapping rate is the fraction of all primary reads with NH ≥ 2. For minimap2 DRS BAMs, the NH tag is not written. Multi-mapping was instead quantified from the alignment-per-read distribution: for each read identified by its QNAME, all mapped records in the BAM were counted using a single-pass awk hash table (samtools view -F 0 × 804 | awk ‘{count[$1]++}’), and the frequency distribution of counts was computed. The multi-mapping rate is reported as the fraction of reads with more than one alignment record, derived from this distribution.

#### Per-transcript read counts

Primary alignments for each BAM were extracted with samtools view -F 0 × 900 -b and re-sorted and indexed, then per-transcript read counts were obtained with samtools idxstats.

#### Per-transcript coverage depth

Mean per-transcript coverage depth and quantized coverage fractions (0×, 1 to 4×, 5 to 9×, 10 to 49×, and ≥50×) were computed with mosdepth using the flag –quantize 0:1:5:10:50: (Pedersen and Quinlan 2018).

#### Antisense alignment rate

For each BAM, primary reads mapping to the reverse strand (SAM flag 0 × 10) were counted with samtools view -F 0 × 904 -f 0 × 10 -c. The antisense rate is the fraction of all primary mapped reads. Because transcriptome FASTA references present all sequences in the sense orientation, a reverse-strand primary alignment represents an antisense read. Note that STAR suppresses antisense alignments by default when aligning to a transcriptome reference; antisense rates for STAR transcriptome BAMs are therefore not interpretable and are reported as not applicable.

### Soft-clip analysis (DRS only)

Soft-clip statistics were computed for DRS transcriptome BAMs using a custom Python script (softclip_metrics.py) implemented with pysam. For each primary alignment, the CIGAR string was parsed to identify leading and trailing soft-clip operations (CIGAR code S, pysam code 4). The lengths of soft-clipped bases at the 5′ and 3′ ends of the read were extracted. For reads mapping to the reverse strand, 5′ and 3′ assignments were swapped to express clipping relative to transcript (rather than read) orientation. Transcripts with fewer than 5 primary alignments were excluded. Per-transcript summary statistics computed include: mean total soft-clip length per read (5′ + 3′ combined); mean clip fraction (total soft-clip bases divided by read length); and asymmetry, defined as the mean per-read difference between 5′ and 3′ soft-clip length.

### Metagene coverage profiles

To compare how each transcriptome reference captures read depth in the vicinity of the ORF start and the ORF end, metagene profiles were computed directly from BAM files using a custom Python script (compute_metagene.py) implemented with pysam, without relying on deepTools.

For each reference, per-base coverage across every transcript sequence in the BAM was computed using pysam.AlignmentFile.count_coverage, restricted to primary alignments (secondary and supplementary reads excluded via a read callback). No additional normalization was applied beyond per-transcript averaging; the profiles represent mean per base read depth (reads per base) at each position, averaged across all transcripts in the restricted gene set. Genes whose ORF is shorter than 200 nt were excluded. This floor is applied to the coding length rather than to the transcript length, and it leaves 4700 genes for the two naive references and 4696 for our annotation and for Nagalakshmi et al., identically for both technologies and both anchors.

Two metagene profiles were computed per BAM, one anchored at the ORF start and one at the ORF end. These anchors are ORF boundaries within the transcript, not transcript ends. The distinction matters, because the region upstream of the ORF start is the annotated 5′ UTR and is present in the reference wherever a 5′ UTR was called. For the ORF start profile, a vector of length 2 × 1000 nt was initialized to zero. Positions [−1000, 0) were filled from the min(1000, *u*) nucleotides lying immediately upstream of the ORF start, where *u* is the annotated 5′ UTR length in that reference, and positions [0, 1000) from the first min(1000, *C*) nucleotides of the ORF, where *C* is the coding length. The ORF end profile was constructed analogously, from the final min(1000, *C*) nucleotides of the ORF and the min(1000, *d*) nucleotides of annotated 3′ UTR that follow it. Where a reference provides no sequence at a position, that position contributes a zero, so the far flanks of every profile average real depth together with padded zeros. This affects all four references alike and is the reason the profiles are read at 100 nt from each anchor rather than at the extremes. The mean and standard error of the mean were then computed position by position across all included genes.

This design allows direct comparison of the four references in a shared coordinate system anchored at ORF boundaries. In the ORF-only reference, no sequence extends beyond the annotated ORF start or end, so coverage is zero at all upstream and downstream positions. In the ORF ±1000 nt reference, coverage is present in the ±1000 nt flanks irrespective of UTR length, and may include signal from neighbouring genes given the compact *S. cerevisiae* genome. In the UTR-annotated references (our annotation and Nagalakshmi et al.), coverage beyond the ORF boundaries reflects reads originating from the annotated UTR regions, and its shape and extent directly report on UTR length and coverage uniformity.

## Results

### Coverage segmentation recovers gene-specific UTR boundaries from DRS data

To define transcript boundaries directly from sequencing data, we applied change-point segmentation to per-gene DRS coverage profiles spanning each annotated ORF and its 1000 nt flanking regions using a negative binomial model (see Methods). Because read coverage in this window is highly autocorrelated, the raw output of Segmentor3IsBack is substantially over-segmented: a typical gene yields on the order of dozens of candidate breakpoints, the majority of which reflect local coverage fluctuation rather than genuine transcript structure. Filtering these candidates to retain only transitions where the mean coverage difference across the breakpoint exceeds 10% of the gene’s maximum transcript count collapses this redundancy into a small number of biologically interpretable segments, typically corresponding to the 5′ UTR onset, the expressed ORF, and the 3′ UTR decay (Fig. 1a).

Figure 1 illustrates this process for a representative gene, YBL091C, chosen for its clean, well-separated data. Raw DRS coverage over the ORF ± 1000 nt window (Fig. 1bi) shows the expected profile: a sharp rise at the transcription start, a stable plateau across the expressed region, and a decline towards the 3′ end. The unfiltered segmentation (Fig. 1bii) identifies 28 breakpoints distributed throughout the plateau, the majority spurious. After filtering (Fig. 1biii), only the 10 breakpoints flanking the true expressed region remain, and the resulting boundaries (Fig. 1biv) extend the 5′ UTR from the 8 nt reported by Nagalakshmi et al. (2008) to 21 nt while leaving the 3′ UTR unchanged (352 nt), illustrating that the algorithm extends boundaries only where DRS coverage provides direct support for doing so, rather than systematically inflating every call.

A recurring complication in the compact *S. cerevisiae* genome is read-through from a neighbouring gene, which produces a secondary peak adjacent to the focal gene’s true expressed region. Because this secondary peak is separated from the focal plateau by a qualifying breakpoint, the filtering step correctly excludes it from the called UTR rather than incorporating it as additional transcript length (Supplementary Fig. S3 shows additional examples, including genes with overlap at one or both boundaries).

The backbone gene model contains 6607 genes, the same set annotated by Nagalakshmi et al. Segmentation was attempted for every gene with more than 20 reads at its position of maximum coverage, and returned a boundary call for 5682 genes (5770 in total, before excluding 88 genes absent from the backbone; Supplementary Table S2), or 86.0% of the backbone. Those calls are non-zero at the 5′ end for 5495 genes and at the 3′ end for 5648 genes, before any merging.

Because the merge is a union rather than an intersection, the final UTR table is larger than the gene model. It carries 6695 rows: the 6607 backbone genes, plus 88 genes that received a DRS call without a corresponding gene record. Of those 88, 84 are ORFs that the backbone represents by a coding sequence and its mRNA parent but not by a gene feature. The remaining 4 (YFL056C, YLL016W, YPL275W, and YPL276W) are absent from the backbone altogether, so their calls do not reach the distributed sequence files. Within the merged table the reference provides a non-zero 5′ UTR for 6005 genes and a non-zero 3′ UTR for 6037 genes, with 5977 genes annotated at both ends. The distributed transcriptome FASTA contains 6767 sequences, one for each mRNA parent in the backbone, and that figure is identical for our reference and for the Nagalakshmi reference because both are built on the same backbone. Supplementary Table S2 gives the count at every stage and reconciles the 6607 gene model, the 6695 row UTR table, and the 6767 distributed sequences.

Boundary calls are not distributed evenly across ORF classes. Using the SGD R64-4-1 classification, DRS called a boundary for 93.9% of the 5255 Verified ORFs, 61.1% of the 668 Uncharacterized and 49.4% of the 684 Dubious (chi-square *P* < 10⁻²⁹⁹, Cramér *V* = 0.46). The released annotation assigns Dubious ORFs a median UTR of 86 nt at the 5′ end and 20 nt at the 3′ end, both well short of the Verified class medians of 97 and 160 nt, so it makes a comparatively modest claim about them. Because Dubious ORFs are also short, with a median span of 348 nt against 1278 nt for Verified, the deficit was tested with ORF span held constant. Stratifying by ORF span decile, the Mantel-Haenszel odds ratio for a DRS call, Dubious against Verified, is 0.31. Agreement with the independent TIF-seq boundaries follows the same order, with under-calls at the 20 nt tolerance rising from 3.8% of Verified to 25.8% of Dubious ORFs at the 5′ end (Supplementary Table S3).

Additionally, UTR length can be influenced by environmental and physiological conditions (Tuller et al. 2009, Lin and Li 2012). The inclusion of transcripts from both the non-starved condition (NS) and the 10 min glucose starvation condition (S10) may therefore introduce a modest condition-dependent bias in the final annotation. To assess this, we modelled log-transformed UTR lengths as a function of condition. For 5′ UTRs, we observed a statistically significant but small reduction in S10 compared to NS (β = −0.099, *P* = 5.7 × 10^−5^), corresponding to an average decrease of ∼9%; however, the effect explained very little variance (*R*^2^ ≈ 0.0016). For 3′ UTRs, no significant difference was detected (β = −0.023, *P* = 0.08; *R*^2^ ≈ 3 × 10^−4^). At the gene level, 69.2% of 5′ UTRs were longer or equal in NS compared to S10, while this proportion reached 78.7% for 3′ UTRs, indicating some directional asymmetry but without a strong shift in global magnitude. Overall, these results suggest that condition-dependent effects are detectable but small, and are unlikely to substantially impact the global properties of the merged annotation.

### The merge rule trades over-extension against truncation, by design

We combined the two sources by taking the longer boundary at each end. Because the final length is by definition the maximum of the DRS estimate and the Nagalakshmi et al. value, the final annotation cannot fall below the Nagalakshmi value at any gene. That is a property of the rule rather than an empirical result, and we state it here as construction rather than as evidence. Its practical consequence is the useful part: the resulting FASTA can be substituted for the Nagalakshmi reference in an existing pipeline without any gene losing annotated sequence.

The reasoning behind the rule is conservatism about the direction of error. An annotation used to assign reads should not shorten a boundary that an established reference already reports, because a transcript that is truncated in the annotation loses reads outright, whereas one that is too long gains only a poorly covered tail. This is not simply a hedge against measurement error: UTR length in yeast is itself condition-dependent, with growth phase and stress state shifting UTR usage genome-wide (Zhan et al. 2025), so a DRS call shorter than the Nagalakshmi value does not by itself mean the Nagalakshmi value is wrong, only that it was measured under different conditions. We see this directly in our own data: segmented separately, before either is merged with Nagalakshmi et al., our own two real physiological conditions disagree with each other by more than 20 nt for 28.6% of comparable genes at the 5′ end and 13.1% at the 3′ end (Results). That asymmetry is deliberate, and it is not free. Across the 5682 genes carried by both sources the rule leaves most boundaries unchanged, but at the 3052 genes at the 5′ end and 3534 genes at the 3′ end where our own call was shorter, it discards that call and retains 357 997 nt and 466 657 nt of UTR beyond what our data support. Measured against the TIF-seq boundaries of Pelechano et al., which took no part in construction, the retained boundary is the worse of the two at those genes, by a median of 12 nt at the 5′ end and 29 nt at the 3′ end (Wilcoxon signed rank *P* < 10⁻¹⁵², rank biserial correlation 0.55 and 0.54).

We keep the rule despite that cost. The penalty is a bounded over-extension on a minority of genes, whereas the alternative admits truncation, which is the more damaging error for quantification. The choice is also not simply a concession, because a substantial minority of the discarded DRS calls are themselves truncated rather than correct, as we show below. Users who need the unmerged DRS calls will find them in the deposited DRS boundary table.

Extending boundaries in a genome this compact necessarily creates overlaps. Relative to the prior reference, 605 gene pairs newly overlap, with a median overlap of 102 nt. This should be read against the pre-existing burden. The ORF bodies alone overlap in 738 pairs, and the prior reference annotation raises this to 2820, so the merged annotation adds roughly a third as many overlaps as the reference it extends. No previously overlapping pair was resolved, as expected from a merge rule that never shortens a boundary. Of the 6767 transcripts, 5676 (83.9%) gain no new overlap. The subset relevant to stranded quantification, pairs on the same strand where the new span reaches into the partner’s ORF, numbers 102 pairs and affects 200 genes (2.96%). These are listed in Supplementary Table S4 so that users can exclude them.

A second, distinct contribution of this resource is the recovery of UTR boundaries for genes that Nagalakshmi et al. could not annotate at all. Of the 6607 genes in the shared gene set, Nagalakshmi reports no 5′ UTR for 1815 genes and no 3′ UTR for 1790 genes. DRS coverage provided a usable boundary for 927 of these genes at the 5′ end and 896 at the 3′ end. Those counts are restricted to genes that carry a Nagalakshmi record. Counted instead across all 6695 rows of the merged table, which adds the 88 genes with no such record, the figures are 1013 and 984. This resource directly fills roughly half of the annotation gaps left by the original short-read study. Representative examples of high-coverage genes such as YJR029W, YER138C, and YGR027W-B, where DRS show read counts at the position of maximum coverage exceed 1000, leaving little ambiguity that the called region is genuinely transcribed despite the absence of any Nagalakshmi annotation (Fig. 2c–e).

**Figure 2:**
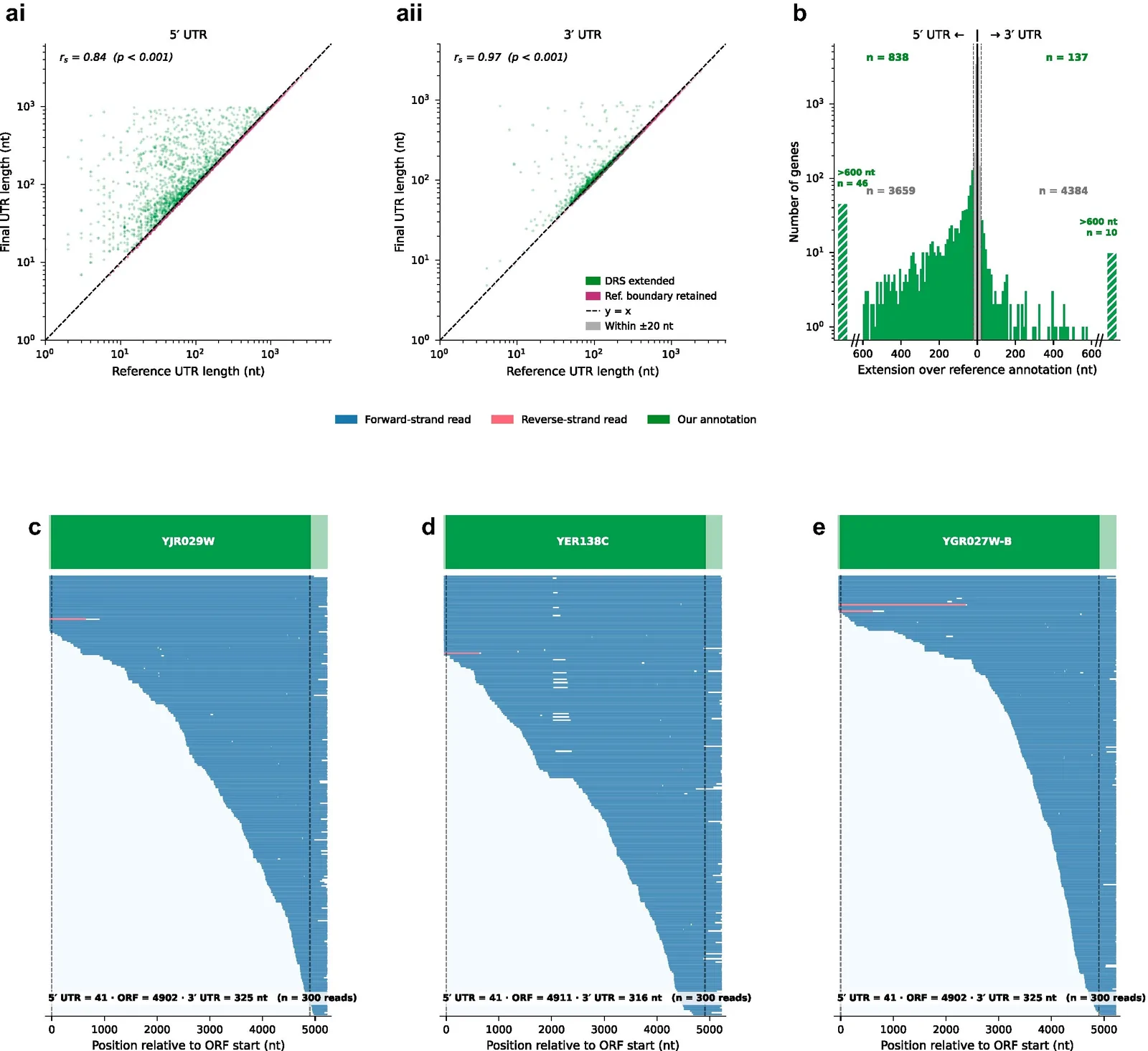
The final annotation agrees with the Nagalakshmi et al. (2008) short read reference and extends it. (ai, aii) Final UTR length against the length in the prior reference, 5′ UTR in panel ai and 3′ UTR in panel (aii), for the genes carrying a non-zero length in both. Both axes are logarithmic. The dashed line marks equality and the grey band marks agreement within 20 nt. No point falls below the line, because the merge rule takes the longer boundary from either source and so can never shorten one. Spearman ρ = 0.84 at the 5′ end and 0.98 at the 3′ end, both *P* < 0.001. The 5′ cloud is the more scattered of the two. (b) Extension of the final annotation over the prior reference, drawn back to back with the 5′ UTR to the left and the 3′ UTR to the right, gene counts on a logarithmic axis. The quantity plotted is an extension and is zero or positive by construction. It is not a signed difference between the two sources; for that comparison see Supplementary Fig. S5. The printed label on each side gives the genes whose final boundary lies within 20 nt of the prior reference, 3659 at the 5′ end and 4384 at the 3′ end. The detached hatched bar beyond the plotted range holds every gene extended by more than 600 nt, 46 at the 5′ end and 10 at the 3′ end, with the count printed on the bar. No gene is dropped from the panel and nothing is stacked onto the terminal in range bin. (c, d, e) Read pileups for YJR029W, YER138C, and YGR027W-B, three genes the prior reference leaves without a UTR. Each panel carries a gene model above and up to 300 sampled primary alignments below, drawn as horizontal bars coloured by strand, against position relative to the ORF start in nt. The decline in coverage towards the 5′ end is the known length dependent signal loss of DRS and not a boundary.

Where both annotations report a UTR, the two are in strong overall agreement. Because UTR length distributions are right-skewed, we report Spearman rank correlation as the primary summary statistic: ρ = 0.850 (5′ UTR, *n* = 4792) and ρ = 0.974 (3′ UTR, *n* = 4817); Pearson correlation gives consistent results (*r* = 0.942 and *r* = 0.980, respectively). The markedly tighter agreement at the 3′ end is consistent with the higher confidence of 3′ boundary calls from DRS data, which is anchored directly at the poly-A tail, whereas 5′ UTR length additionally depends on read completeness through the full length of the transcript (see Discussion). Restricting at each end separately to genes with a non-zero call both sides (i.e. before the longest-UTR merge step), which is 4370 genes at the 5′ end and 4516 at the 3′ end, the raw DRS boundary was actually shorter than the corresponding Nagalakshmi annotation for the majority of genes: 67.3% at the 5′ end and 78.1% at the 3′ end. To exclude differences plausibly attributable to noise in either annotation, we applied a 20 nt minimum-difference threshold; even under this conservative criterion, the DRS-derived boundary was meaningfully shorter than Nagalakshmi for 39% of comparable genes at the 5′ end (median shortfall 66 nt among these) and 59.2% at the 3′ end (median shortfall 104 nt where shorter; Supplementary Fig. S4 shows representative examples, while Supplementary Fig. S5 summarizes the main differences). In every one of these cases, the final merged annotation retains the longer Nagalakshmi value rather than the shorter DRS-derived call, confirming that the longest-UTR rule performs substantial, genuine gene-by-gene arbitration between the two sources rather than defaulting to either one.

As a fully independent validation, we compared the final annotation against the TIF-seq boundaries (Pelechano et al. 2013), an orthogonal method that maps both transcript ends directly and was not used in constructing the reference. Across the genes with a covering major transcript isoform (*n* = 5 448 at the 5′ end and *n* = 5 506 at the 3′ end), the two annotations were positively correlated (Spearman ρ = 0.48 and ρ = 0.51). We report both disagreement tails. At the 20 nt tolerance the final annotation under-called the TIF-seq boundary for 5.2% of genes at the 5′ end (285 of 5448) and 7.2% at the 3′ end (397 of 5506), and over-called it for 54.0% (2940) and 47.9% (2640), respectively. Relaxing the tolerance to 50 nt, which is closer to the reproducibility of the benchmark itself, the over-call rates fall to 37.3% and 36.7%.

Two comparisons place this asymmetry. First, the prior reference alone over-calls TIF-seq at a similar rate, 45.2% at the 5′ end and 49.8% at the 3′ end, so the excess length is largely inherited rather than introduced. Second, the DRS calls alone agree with TIF-seq more closely than the merged table at both ends (Spearman ρ = 0.58 and 0.69, median signed difference +4 nt and +1 nt, against +25 nt and +17 nt for the merged table). The merge rule, which retains the longer of the two sources, is therefore what widens the gap. The largest provenance class at both ends is a boundary where our own segmentation called shorter and the prior value was retained, and it carries the highest over-call rate, 57.7% at the 5′ end and 69.5% at the 3′ end.

Much of the remaining gap reflects a difference in definition rather than an error. The major covering isoform is the most read-supported single isoform, whereas the final annotation is a maximum across conditions and sources. Comparing the released boundary against the full range of ends TIF-seq observed for the same gene, 93.3% of 5′ boundaries and 97.3% of 3′ boundaries fall within an isoform that TIF-seq also recorded. Only 362 genes (6.7%) at the 5′ end and 148 (2.7%) at the 3′ end extend beyond every observed isoform, and these are the only unambiguous over-extensions against this benchmark. The over-call is not an artefact of sparse benchmark sampling. It rises with the read support of the major isoform, from 40.6% to 59.5% at the 5′ end and from 20.4% to 57.8% at the 3′ end, between genes whose major isoform rests on a single read and those supported by 20 or more (Supplementary Figs S2 and S6).

### Robustness of boundary calls to threshold selection

At the released settings, 5770 genes received a boundary call, of which 5768 had a non-zero UTR at one end or both. Overall, the released annotation showed good but not unconditional stability across the explored parameter space.

The coverage floor had the largest effect on boundary calls at both ends. Because the released call is the maximum across six conditions rather than a single library, changing this threshold can admit or withdraw a different condition’s contribution to a given gene’s maximum even when no single condition’s own segmentation changes. A single grid-step change in the coverage floor moved the boundary for 18.0% of genes at the 5′ end and 8.5% at the 3′ end. The effect-size threshold had the next largest influence, moving 14.4% and 7.4% of genes at the 5′ and 3′ ends, respectively. The expression floor and clustering window were comparatively inert, although their relative influence differed between ends: 5.5% and 2.6% of genes moved at the 5′ end, compared with 0.6% and 1.7% at the 3′ end.

Across all 624 non-released combinations, jointly varying all four thresholds by up to four grid steps yielded retained-boundary proportions ranging from 0.087 to 1.000 and identical-boundary proportions ranging from 0.263 to 0.984, with these extrema considered independently across the grid. No single combination attained both extremes simultaneously. The combination yielding the lowest identical-boundary proportion (coverage floor 5, clustering window 160 nt, effect size 35%, expression floor 20%) retained 43.3% of genes and left 26.3% of retained boundaries unchanged.

The released values therefore sit inside a region of good but not unconditional stability: most single-step perturbations leave the great majority of calls untouched, while the extreme corner of the grid moves a substantial minority. Per-combination results, the whole-grid summary and the per-parameter breakdown are given in Supplementary Table S5 and Supplementary Fig. S1.

### Two real construction conditions bound how much a single condition’s DRS call can differ from the merged annotation

The threshold sweep above tests sensitivity to segmentation parameters; it does not test whether a single real experimental condition is itself a reliable proxy for the released, six-condition-pooled annotation. We tested this directly using the two independent physiological conditions behind the six construction libraries, NS and S10, each already the pool of its own two biological replicates. Segmenting each condition separately, before either is merged with Nagalakshmi et al., the two conditions' own DRS calls agree exactly for 21.5% of the 5002 comparable genes at the 5′ end and 42.9% at the 3′ end, and agree within 20 nt for 67.7% and 77.9%. After each condition is independently merged with Nagalakshmi et al., the same way the release itself is built, and checked against the actual released annotation, both conditions individually reproduce the real call for 58.6% of genes at the 5′ end and 73.5% at the 3′ end; only one condition does for 21.7% and 16.7%; neither does for 19.6% and 9.8%. Even in that last category the two conditions mostly land on the same answer as each other (37.8% and 49.7% of the time) rather than disagreeing at random, pointing to a shared, systematic source of movement between conditions rather than independent noise. Full per-gene results are given in Supplementary Table S6.

### Two independent benchmarks disagree with each other as much as either disagrees with this resource

We repeated this comparison against UNAGI (Al Kadi et al. 2020), a transcript annotation built from nanopore full-length cDNA in a different laboratory, which likewise took no part in constructing the present reference. Genes were matched to UNAGI on the genomic coordinate of the start or stop codon rather than through an accession map, which resolved 5509 5′ ends and 5752 3′ ends onto the shared gene model and reproduced exactly the counts of genes with alternative sites reported in that study, 1089 at the 5′ end and 1 482 at the 3′ end. UNAGI gives a median 5′ UTR of 83 nt and a median 3′ UTR of 147 nt. The two resources are not nested: our annotation reports a 5′ boundary for 688 genes that UNAGI leaves uncalled and a 3′ boundary for 500, while UNAGI covers 192 and 215 genes that we leave uncalled.

Across comparable genes, 5317 at the 5′ end and 5537 at the 3′ end, the two annotations were positively correlated (Spearman ρ = 0.46 and ρ = 0.51), matching the correlation obtained against TIF-seq almost exactly. At the 20 nt tolerance the final annotation over-called UNAGI for 34.7% of genes at the 5′ end and 35.0% at the 3′ end, and under-called it for 25.7% and 21.3%. At 50 nt the over-call rates fall to 24.5% and 26.3%. Where over-calls outnumbered under-calls by more than tenfold against TIF-seq, the two tails here are of comparable size.

The difference between the two benchmarks is one of definition. The TIF-seq major isoform is a mode, the single most read-supported transcript, so comparing a maximum against it charges a definition mismatch as error. The UNAGI boundary is itself a maximum: it coincides with the furthest of the alternative sites that study lists, for 2305 of 2305 genes at the 3′ end and 1058 of 1063 at the 5′ end. The rates above therefore require no envelope correction and are already like for like. UNAGI further records how far apart the ends it observed for one gene lie. That spread has a median of 23 nt at the 5′ end and 9 nt at the 3′ end, and reaches or exceeds 20 nt for 54.6% and 36.8% of the genes concerned, so a tolerance narrower than the dispersion of the benchmark itself measures isoform heterogeneity rather than annotation error.

Splitting by boundary provenance reproduces the pattern seen against TIF-seq. The largest class at both ends remains a boundary where our own segmentation called shorter and the prior value was retained, and it carries the highest over-call rate, 38.2% at the 5′ end and 51.6% at the 3′ end. Paired on identical gene sets, the merge left agreement with UNAGI unchanged at the 5′ end (761 genes closer, 525 further, 3250 tied, *p* = 0.58) and improved it at the 3′ end (747 closer, 139 further, 3729 tied, Wilcoxon signed rank *P* = 2.6 × 10⁻⁸⁰).

Setting the two benchmarks against each other calibrates every rate above. On the genes both cover, 5160 at the 5′ end and 5314 at the 3′ end, UNAGI and TIF-seq agree with each other within 20 nt for only 48.0% and 56.8% of genes (Spearman ρ = 0.58 and ρ = 0.65). On those same genes our annotation agrees within 20 nt with UNAGI for 40.2% and 43.1%, with TIF-seq for 42.0% and 45.4%, and with both at once for 27.0% and 31.9%. Two established boundary sets built from different chemistries therefore disagree with each other on roughly half of all genes, and this resource sits at an almost equal distance from each. Disagreement on this scale is the ordinary condition of UTR boundary calls across technologies rather than a property of any one annotation, and it sets a practical ceiling on how closely any two such resources can be expected to agree (Supplementary Table S7).

### Where our own calls were shorter, the prior reference is more often over-extended than we are truncated

Our DRS segmentation called a shorter UTR than the prior reference for 3070 of 4497 genes at the 5′ end and 3534 of 4521 at the 3′ end, with a median difference of 28 nt and 73 nt. Because the merge takes the longer of the two calls, these are the genes where the released annotation departs from its own primary evidence. We therefore asked whether the shorter call reflects a shorter transcript or a limitation of the sequencing. Against TIF-seq, which took no part in constructing the annotation, the DRS call was the closer of the two for 67.4% of these genes at the 5′ end and 69.0% at the 3′ end (median absolute error 12 nt against 29 nt, and 9 nt against 51 nt, sign test *P* < 10⁻⁸⁶ at both ends). The verdict strengthened when the benchmark was restricted to isoforms supported by at least 20 reads, to 72.5% and 75.1%. It reversed on the genes where our call was longer instead, where the prior reference was closer for 80.9% and 58.5%.

TIF-seq lay beyond both calls, the only configuration in which sequence is demonstrably lost, for 701 genes at the 5′ end (23.0%) and 734 at the 3′ end (20.8%). Within the shortfall the odds of that configuration rose 5.16 fold per ten fold increase in coding length at the 5′ end (95% CI 3.63 to 7.34, *P* = 6.7 × 10⁻²⁰) and, more modestly but still detectably, 2.33 fold at the 3′ end (95% CI 1.67 to 3.26, *P* = 7.0 × 10^-7^), and fell with sequencing depth at both ends.

Read geometry gives the same result without reference to any UTR call. Of 10 237 196 primary alignments, 34.1% ended within 10 nt of the annotated 3′ end but only 3.7% reached within 10 nt of the 5′ end. Across deciles of transcript length the fraction reaching the 5′ end fell from 16.2% to 1.7%, while the fraction reaching the 3′ end rose from 34.5% to 52.8%. We therefore attribute the shortfall to a mixture. In the majority of cases, the prior annotation was over-extended and the shorter DRS call is the more accurate of the two. In roughly a fifth of cases at the 5′ end and a sixth at the 3′ end, the DRS call is genuinely truncated, with a length dependence at the 5′ end that matches the 3′ to 5′ direction of DRS (Supplementary Fig. S7).

### The merged reference improves read mapping relative to naive alternatives, on both long- and short-read data

To evaluate the practical effect of UTR definition on downstream alignment, we mapped both an independent DRS data (Fonzino et al. 2026) and an independent short-read dataset (Al Kadi et al. 2020) to five references built from the same underlying gene models: the full genome, an ORF-only transcriptome, a transcriptome with a fixed ±1000 nt flank around each ORF, the Nagalakshmi et al. annotation alone, and our final merged annotation (‘our ref’). Because the fixed-flank and ORF-only references share no biological basis for their boundaries, they serve as naive baselines against which to judge whether UTR-aware references offer a genuine improvement, and whether that improvement extends beyond the data used to construct it.

For the DRS data (3 423 258 primary reads), mapping to our reference recovered a 94.14% mapping rate, closely matching the genome (95.21%) and Nagalakshmi (93.37%) references and substantially exceeding the ORF-only transcriptome (85.38%), which by construction provides no homology target for reads originating in UTRs (Fig. 3a). Critically, this gain in mapping rate was not accompanied by the loss of specificity seen in the naive ±1000 nt flank reference, which achieved a similar mapping rate (94.36%) but at a multi-mapping rate of 84.21%, or more than five times that of our reference (15.31%), reflecting the fact that fixed flanks routinely capture sequence from neighbouring genes in the compact yeast genome (Fig. 3b). Antisense mapping was lowest for our reference of all five (5.96%), below both Nagalakshmi (6.62%) and ORF-only (8.59%), indicating that the merged boundaries are not simply longer but remain well localized to genuine sense-strand transcript sequence rather than incidentally capturing adjacent or antisense signal (Fig. 3c).

**Figure 3:**
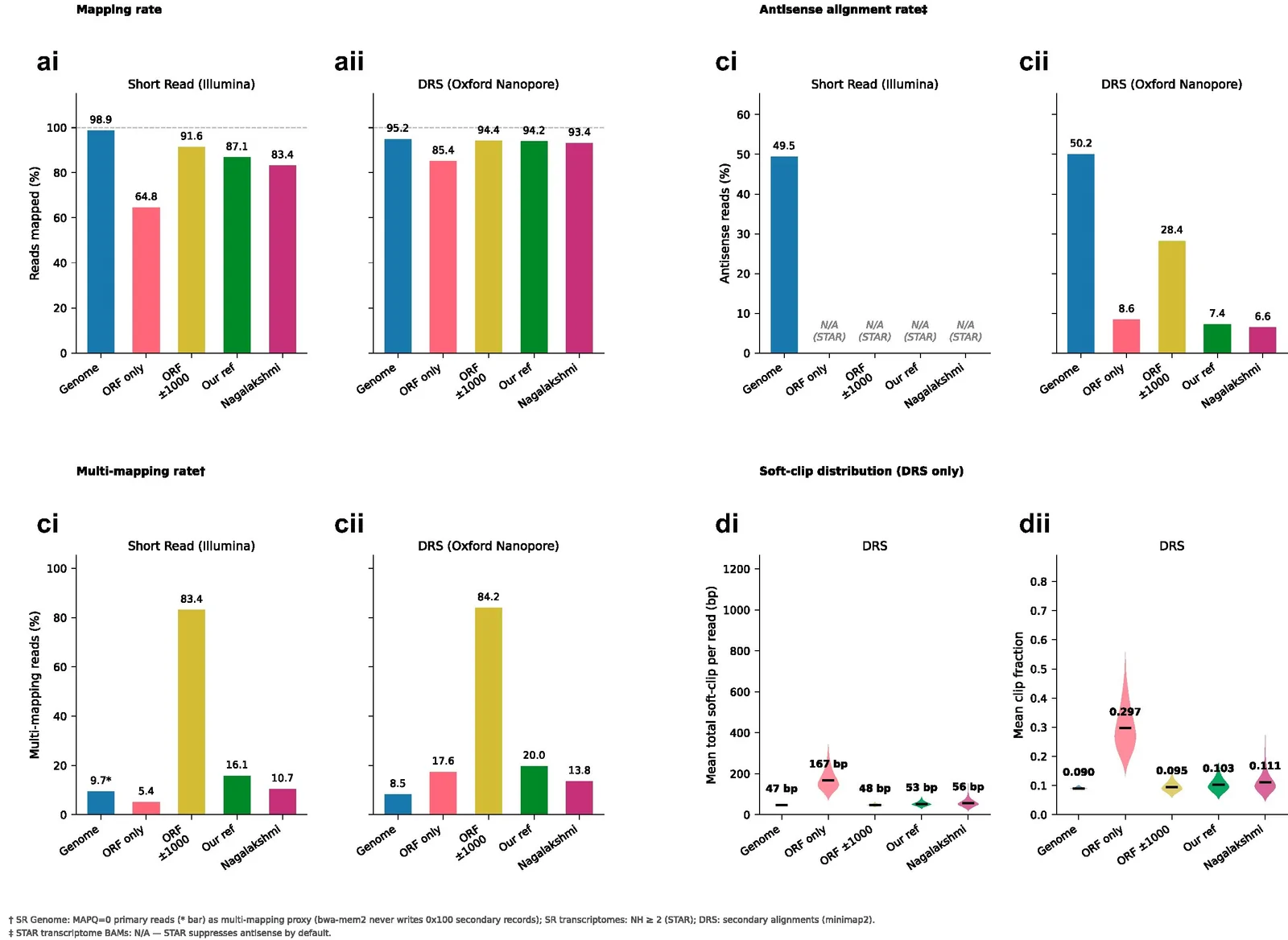
Alignment quality across five reference transcriptomes, for short read (Illumina) and DRS. (ai, aii) Mapping rate, short read in panel ai and DRS in panel Aii, as the percentage of input reads carrying a primary alignment. The denominator is the raw FASTQ read count for short reads and the total records in the BAM including unmapped for DRS. The dashed line marks 100%. (bi, bii) Multi-mapping rate. Three definitions are in play because the five references are not all aligned by one tool, and they are not interchangeable. For the genome reference aligned with bwa-mem2, marked with an asterisk, multi-mapping is the fraction of primary alignments with MAPQ = 0, because bwa-mem2 reports no secondary alignments. For the STAR aligned transcriptome references, it is the fraction of reads carrying an NH tag of 2 or more. For DRS, aligned with minimap2, it is the fraction of reads with at least one secondary alignment. The high rate for ORF ±1000 nt follows from the transcript overlap that fixed flanks create in a genome this compact. (ci, cii) Antisense alignment rate, primary mapped reads only. STAR suppresses antisense alignments to transcriptome references by default, so those references are marked N/A rather than zero. (di, dii) Per transcript soft-clipping, DRS only. Panel (di) gives the mean total soft-clipped bases per read and panel (dii) the mean soft-clip fraction, clipped bases divided by read length. Violin bodies show the distribution across transcripts and the horizontal bar marks the median, which is printed. The ORF only reference clips most, which is what reads running past a reference that stops at the ORF must do. This reference and the Nagalakshmi reference clip similarly little, close to the full genome reference.

A complementary line of evidence comes from soft-clip length, which measures how much of each aligned read falls outside the reference sequence rather than whether the read maps at all. Soft-clip length was compared per transcript rather than per read, since the same reads are realigned to each reference and are not independent observations. Across the 5 862 transcripts annotated in both our reference and Nagalakshmi and carrying at least five primary alignments under each, the released reference clipped less for 3236 transcripts and more for 606, with 2020 unchanged (Wilcoxon signed rank *P* below 10^−300^, matched pairs rank biserial correlation 0.80). Restricted to the 3070 transcripts whose boundaries were actually extended, the median reduction is 4.4 nt per read (95% CI 3.9–4.9), and the reduction scales with the amount of UTR added (Spearman ρ = 0.55). For scale, the same test against the ORF-only reference gives a median reduction of 108.3 nt. The effect relative to the prior reference is therefore consistent and mechanistically interpretable, but modest.

The distributional summary agrees. Median total soft-clip length (5′ + 3′ combined) was 52.6 nt for our reference against 55.7 nt for Nagalakshmi, and lower at each end individually (5′ 3.2 against 3.6 nt, 3′ 47.9 against 49.9 nt). Because soft-clipped bases represent sequence the aligner could not place within the reference, this indicates that our boundaries capture more of the true transcript within the annotated sequence itself, rather than leaving it to be clipped at alignment time. The ORF-only reference shows by far the highest clip burden of the four (median 166.9 nt), as expected for a reference that provides no UTR sequence at all. The ±1000 nt flank reference shows the lowest absolute clip values of any reference (48.1 nt), but this reflects the generosity of a flank far larger than any real UTR rather than a more accurate boundary, and should be read alongside its substantially worse multi-mapping and antisense rates above rather than as evidence of superior boundary placement (Fig. 3d).

This pattern was independently reproduced on short-read data that played no role in constructing the annotation. Of 34 270 624 total short reads, 29 835 578 (87.06%) mapped to our reference, compared with 28 594 996 (83.43%) to Nagalakshmi and 22 216 695 (64.82%) to the ORF-only transcriptome, a gain of ~1.2 million additional mapped reads relative to Nagalakshmi alone, recovered without any change to the underlying sequencing data. As with the DRS data, the ±1000 nt flank reference reached a nominally higher mapping rate (91.63%) only by accepting a correspondingly inflated multi-mapping rate (83.37% vs. 16.07% for our reference), confirming that this naive baseline trades specificity for coverage rather than offering a genuine improvement. Together, these results show that the merged reference inherits the clean mapping behaviour of the Nagalakshmi annotation while recovering additional correctly mapped reads, on both the data used to build it and an independent dataset, and does so without the multi-mapping cost of simply widening the reference indiscriminately. Supplementary Fig. S8 shows representative read pileups from both independent datasets for direct visual comparison.

### Changing the reference moves few abundance estimates, and the movement concentrates on newly overlapping genes

A higher mapping rate is only useful if the reads are placed on the right genes. To test this, reads from an independent direct RNA library (SRR32918129) and from two published short-read libraries were aligned to the prior reference and to the released reference in the same run with identical settings, and per-gene abundance was estimated as best-hit counts normalized to transcripts per million. Estimates remain concordant between the two references, with Spearman ρ = 0.96 on direct RNA and 0.98 on short reads, and 83.3% and 85.8% of genes within 1.2 fold, respectively. On direct RNA, 4.5% of genes move by twofold or more, and 2.7% on short reads. Following individual reads, 85.4% keep the same best-hit gene on direct RNA; 0.77% are recovered by the released reference that were unmapped under the prior one, and 47 of 3 423 258 are lost. On short reads, no reads are lost and 3.0% are recovered.

The genes whose extensions newly reach a neighbouring ORF on the same strand (*n* = 161 on direct RNA, 198 on short reads) do move more than the rest, though the effect is modest: Cliff’s delta is 0.124 (*P* = 0.007) and 0.152 (*P* = 3 × 10⁻⁴), with 8.7% and 5.6% of them moving twofold against 4.4% and 2.6% of other genes. At the read level, 0.33% of direct RNA reads and 0.60% of short reads are assigned to a different gene across one of these 605 newly overlapping pairs. The large majority of this figure arises at a single locus, the RDN37 rDNA repeat, on both technologies (84% of direct RNA, 75% of short read)—a known multi-mapping sink independent of this annotation. These counts are an upper bound, because a read crossing a newly overlapping pair is not necessarily assigned incorrectly.

### Metagene coverage confirms that extended boundaries capture genuine transcript signal

The mapping-rate comparisons establish that more reads map to our reference overall, but not where those reads map relative to the annotated boundary. To address this directly, we computed metagene coverage profiles anchored at the ORF start and the ORF end for all four transcriptome references, using a shared ORF-relative coordinate system so that mean read depth at a given position is directly comparable across references, and restricting to genes with defined Nagalakshmi *et al*. UTR bounds and an ORF of at least 200 nt, which leaves 4696 genes for our reference and for Nagalakshmi et al. and 4700 for the two naive references (Methods).

The ORF-only reference shows the expected null result by construction in both data types: mean read depth is exactly zero at every position upstream of the annotated start and downstream of the annotated end (DRS: 467.9 reads per base at −10 nt falling to 0 from position 0 onwards at the ORF end; short reads: 276.3 reads per base at −10 nt falling to 0 at the same boundary), since no sequence exists there for reads to align to (Fig. 4).

**Figure 4:**
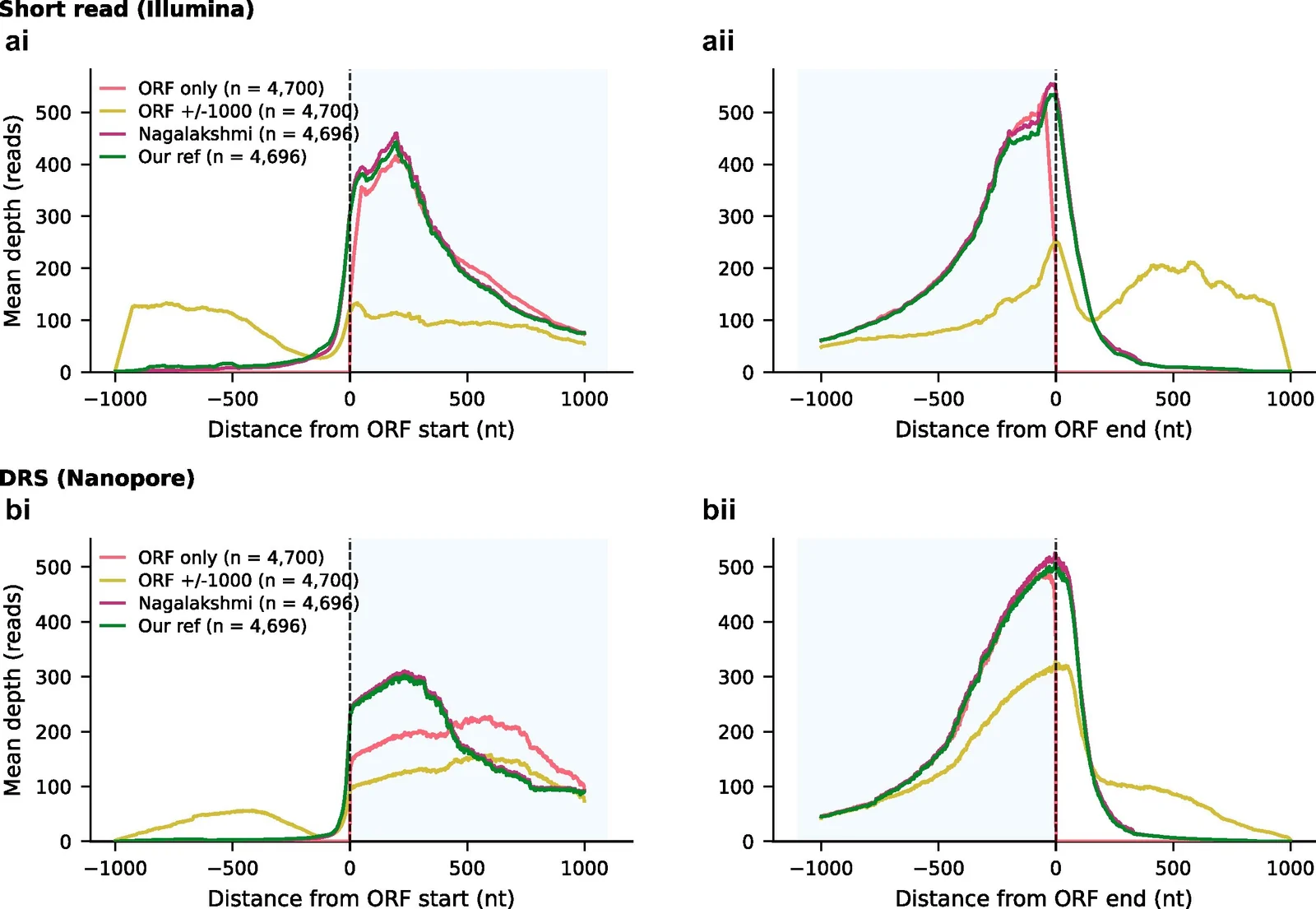
Metagene coverage around the ORF boundaries for four reference transcriptomes. Mean read depth against distance from the annotated ORF boundary, which sits at position 0 and is marked with a dashed line, over the genes shared by all four references. Light shading marks the side of the boundary lying inside the ORF. Four references are overlaid: ORF only, ORF ±1000 nt, the merged annotation from this study, and Nagalakshmi et al. (2008). Each curve carries its own n on the panel, 4700 transcripts for the two ORF references and 4696 for the other two. The difference is a 200 nt minimum ORF length applied in the metagene code and stated in Methods. (ai, aii) Short read (Illumina) profiles at the ORF start and at the ORF end. This study and Nagalakshmi are near identical. The ORF only reference tracks them inside the ORF and drops abruptly at the boundary, because no sequence exists beyond it for a read to align to. ORF ±1000 nt is depressed throughout, consistent with its multi-mapping rate in Fig. 3b: fixed flanks in a compact genome spread signal across overlapping transcripts. (bi, bii) DRS profiles at the same two anchors. The gradient across the ORF in panel (bi) is the signature of reads anchored at the poly(A) tail, with coverage rising from left to right as fewer reads reach the 5′ end. At the ORF end in panel (bii), this study and Nagalakshmi again coincide, with a sharp peak at position 0. Two cautions. These profiles zero pad wherever the reference sequence runs out, so a far flank averages real zeros together with positions that do not exist. The effect grows with distance from the boundary, and curve shape beyond a few hundred nucleotides should not be read as depth. Supplementary Fig. S9 avoids the problem by truncating each curve on support instead. Separately, a metagene pooled over every gene cannot show much effect of changing an annotation, because most genes were not changed: 3876 of 4696 genes have an unchanged boundary at the ORF start and 4496 at the ORF end. The near identity of this study and Nagalakshmi here is largely a statement about the genes that did not move. Supplementary Fig. S9 splits the same genes on whether the boundary moved.

The ±1000 nt flank reference, in contrast, does not merely show residual coverage in its fixed flank: it shows coverage that approaches, and in places exceeds, the level seen within the ORF body itself, the clearest possible signature of contamination from neighbouring genes. In the DRS data, coverage at 500 nt upstream of the ORF start reaches 53.9 reads per base in the ±1000 nt reference, compared with a typical ORF-body level of around 121 reads per base measured at 800 nt into the gene in the same reference, i.e. coverage nearly halfway back to ORF levels at a position that, in our reference and in Nagalakshmi et al., has already decayed to background (2.2 and 1.6 reads per base respectively). At the ORF end, the effect is more pronounced still: coverage 500 nt downstream of the annotated end reaches 86.3 reads per base in the ±1000 nt reference, *higher* than the 63.3 reads per base measured 800 nt before the boundary in the same reference, while our reference and Nagalakshmi have decayed to 7.1 reads per base at the same distance. The short-read data show the same pattern in even starker form: at 500 nt upstream of the ORF start, ±1000 nt coverage reaches 111.3 reads per base, exceeding the reference’s own ORF-body coverage of 82.6 reads per base measured at 800 nt into the gene, and at 500 nt downstream of the ORF end, it reaches 198.6 reads per base against an ORF-body level of 64.2 reads per base measured at 800 nt before the boundary in the same reference, i.e. in these cases, coverage in the fixed flank is, if anything, higher than coverage inside the annotated coding sequence. This pattern is only explicable by reads originating from the coding sequences of adjacent genes mapping indiscriminately into the fixed flank of the focal gene, a direct consequence of the dense, closely packed gene arrangement of the *S. cerevisiae* genome; it cannot reflect genuine UTR signal for the focal transcript; and it is precisely the artefact that boundary-aware references, by construction, are immune to.

Our reference and the Nagalakshmi et al. reference both show the expected biological profile at each boundary in both data types: a gradual rise in mean read depth approaching the ORF start (DRS: 0.57–227 reads per base between −1000 nt and the annotated ORF start for our reference, 0.60–228 reads per base for Nagalakshmi et al.; short reads: 1.71–305 reads per base for our reference, 1.76–307 reads per base for Nagalakshmi) and a corresponding gradual decay beyond the ORF end, rather than the step-function seen in ORF-only or the inflated, non-decaying tail seen in the ±1000 nt reference. The two UTR-aware references are essentially indistinguishable at the ORF boundary itself; at positions further into the upstream UTR region (e.g. 500 nt upstream of the ORF start, where our reference shows 2.2 vs. 1.6 reads per base for Nagalakshmi et al.), our annotation produces marginally higher mean depth, consistent with a subset of genes carrying extended 5′ UTRs that contribute signal at those upstream positions. The reproduction of every feature of this pattern in the independent short-read dataset rules out the possibility that the extended boundaries are a DRS-specific mapping artefact.

A metagene pooled over every gene cannot show much effect of changing the annotation, because most genes were not changed, and Fig. 4 confirms this by placing our curve on the Nagalakshmi et al. curve in both technologies. We therefore split the same genes by whether the final annotation moved that boundary outwards by more than 20 nt, which separates 830 extended from 3866 unchanged genes at the ORF start and 123 from 4573 at the ORF end (Supplementary Fig. S9). Extended genes carry more signal outside the ORF than unchanged genes at both boundaries and in both technologies. At 100 nt upstream of the ORF start, the short-read depth ratio is 1.73, and at 100 nt past, the ORF end the direct RNA ratio is 1.10. The ordering survives per-gene scaling, which removes between-gene expression differences by expressing each gene’s depth relative to its own ORF mean and summarizing by the median across genes. On that scale, the direct RNA ratio 100 nt upstream of the ORF start is 1.78. Curves are truncated where fewer than one gene in five still has reference sequence, so no part of a curve rests on a handful of long genes. Comparisons further than about 300 nt past the ORF end are not supported and are not reported.

## Discussion

### A new transcriptome reference for yeast, built for direct use

The yeast research community has lacked an updated, readily usable UTR annotation since Nagalakshmi et al. established the field-standard reference using short-read tiling arrays and early RNA-seq (Nagalakshmi et al. 2008). That resource has been remarkably durable, but it carries the structural limitations of the technology that produced it: short-read coverage declines gradually towards transcript ends, and boundary calls made from declining coverage are inherently conservative, with no annotation possible at all for genes whose expression or local genome architecture left the signal too ambiguous to call. DRS removes this limitation at its source by capturing each transcript as a single molecule from 3′ poly(A) to (where read length allows) the immediate vicinity of 5′ cap, making both UTR boundaries directly observable on the same read rather than inferred from the decay of pooled short-read coverage.

The contribution of this work is not simply a new annotation but a complete, directly substitutable transcriptome reference: a merged FASTA, derived by retaining the longer UTR call between our DRS-based segmentation and the Nagalakshmi annotation at every boundary, together with the documented and reproducible pipeline used to build it. This dual deliverable matters for two different audiences. For most users, the FASTA itself is the resource: a drop-in replacement for any existing yeast RNA-seq workflow currently built around Nagalakshmi et al. or an ORF-only reference, requiring no reprocessing of raw data and no familiarity with the underlying segmentation method. For users with their own DRS data, or who wish to apply the same logic to a different strain, condition, or organism, the pipeline itself is reusable, and we provide it openly for that purpose.

### Improvement over the Nagalakshmi 2008 reference

Three lines of evidence in this work support the claim that the merged reference is a genuine improvement rather than a cosmetic update. First, the merge construction guarantees that no gene loses annotated UTR sequence relative to Nagalakshmi et al., a property we confirmed held without exception across thousands of comparable genes (Results). Second, where DRS coverage supports a longer call, it does so for a substantial minority of genes at each boundary; however, most importantly, it supplies a usable boundary for roughly half of the genes Nagalakshmi could not annotate at all, recovering information that has simply been absent from the field’s reference annotation for over a decade. Third, and most importantly, for practical adoption, these extensions are not an artefact of the merge construction: independent mapping-quality and metagene analyses, performed on data not used to build the annotation, show that reads in the extended regions behave exactly as genuine transcript signal should: they map with high specificity, are not enriched for soft-clipping, and form a smooth, biologically shaped coverage profile rather than the elevated, non-decaying signal seen when a reference’s flank is defined arbitrarily rather than from data.

The asymmetry between the 5′ and 3′ results is itself informative. Agreement between our calls and Nagalakshmi’s is markedly tighter at the 3′ end (Spearman ρ = 0.979) than at the 5′ end (ρ = 0.860), and the mean extension at the 5′ end is several-fold larger than at the 3′ end. This is consistent with the underlying biology of DRS: sequencing proceeds 3′–5′ from the poly-A tail, so the 3′ boundary is anchored directly and with high confidence on essentially every read that maps to a transcript, while the 5′ boundary is only as reliable as the longest reads that survive intact to the start of the molecule (Workman et al. 2019). Shorter or degraded reads systematically under report 5′ UTR length, meaning our 5′ UTR calls likely still understate the true 5′ boundary for at least some genes, even after the conservative merge with Nagalakshmi et al. This is a meaningful caveat for any 5′-UTR-specific analysis (translation initiation, upstream ORF usage) built on this resource, and is taken up further below.

### Relationship to comparable long-read efforts in yeast

The most directly comparable prior effort is the UNAGI pipeline of (Al Kadi et al. 2020), which applied Nanopore long-read sequencing of full-length cDNA to improve transcript annotation in *S. cerevisiae*, including 5′ and 3′ UTR boundaries. The present work differs from UNAGI in scope, technology, and deliverable. In scope, UNAGI’s primary contribution is the discovery of novel transcripts and isoforms genome-wide, with UTR annotation as one component of a broader transcript-reconstruction pipeline. Here, UTR characterization relative to an existing, near-complete gene model is the central question, allowing a more targeted and exhaustively validated treatment of UTR length specifically. In technology, the present work uses DRS, which sequences native RNA without a reverse-transcription or Polymerase Chain Reaction (PCR) amplification step and is therefore not subject to the strand and length biases that can accompany cDNA synthesis; UNAGI, in contrast, sequenced full-length cDNA. In deliverable, this work produces an explicit, principled merge against the existing community reference whereas UNAGI’s output is a transcript reconstruction rather than a backward-compatible drop-in replacement for the Nagalakshmi et al. annotation specifically. These distinctions are now supported by a direct gene level comparison (Results). The two annotations agree within 20 nt for 39.6% of genes at the 5′ end and 43.7% at the 3′ end, and each covers genes the other leaves uncalled, 688 and 500 in our case against 192 and 215 in theirs, so neither supersedes the other. That level of agreement is, however, no worse than the agreement between UNAGI and TIF-seq themselves, which is the relevant yardstick.

More broadly, this work sits alongside a small but growing body of long-read transcriptomics in yeast, including UNAGI and the FLAIR-based comparisons it reports (Tang et al. 2020), which collectively make the case that short-read-derived reference annotations from the 2000s era systematically understate UTR extent in compact genomes. We see this resource as complementary to, rather than a replacement for, that broader effort: where UNAGI and similar tools are best suited to discovering novel transcripts and isoforms, the present resource is purpose-built for users whose primary need is an accurate, ready-to-use UTR annotation compatible with the existing Nagalakshmi-based literature.

### The choice to take the maximum UTR length, and its limitations

The central design decision in this work, to retain the longer of the DRS-derived and Nagalakshmi et al. UTR length at each boundary independently per gene and per side, was made to prioritize backward compatibility and safety of adoption over maximal data-driven accuracy. We discuss here both the justification for this choice and its limitations.

The justification is principally practical. A new annotation that replaced Nagalakshmi et al. outright would require every adopting laboratory to evaluate whether the new resource introduces regressions relative to whatever analyses they currently run on the established annotation; for most users, the cost of that evaluation outweighs the benefit of a few hundred nucleotides of additional UTR sequence on a subset of genes. By construction, the longest-UTR merge cannot regress any existing analysis built on Nagalakshmi et al. coordinates: any region annotated by Nagalakshmi et al. remains annotated, and the only possible change is the inclusion of additional sequence beyond what Nagalakshmi et al. reported.

This choice has two related limitations. First, it is conservative by design and therefore does not correct cases in which the Nagalakshmi et al. annotation may be too permissive at a locus; for instance, where short-read coverage extended into what later, longer-read evidence would identify as the start of a neighbouring transcript. The merge logic has no mechanism to shorten a Nagalakshmi et al. call, even where DRS evidence might support doing so; we confirmed that such cases do occur (the DRS-derived boundary was, for more than half of the genes, shorter than the corresponding Nagalakshmi et al. call; see Supplementary Fig. S5), and a fully data-driven re-annotation, unconstrained by backward compatibility, would in principle be free to revise such cases. We view this as an acceptable trade-off for a resource paper rather than a shortcoming to be corrected: a maximally accurate but non-backward-compatible annotation and a conservative, backward compatible one serve different purposes, and we have deliberately built the latter.

Second, and more subtly, ‘maximum across sources’ is not the same guarantee as ‘correct’. Because our own DRS-derived estimate is itself taken as the maximum UTR length observed across all sequencing conditions and RNA fractions (Methods), and is then merged with Nagalakshmi et al. by retaining whichever of the two is longer, the final annotation is, by construction, the longest plausible UTR call available to us from any source examined. This maximizes sensitivity at the cost of an increase in false-positive UTR length for at least some genes, for example where a single sequencing condition or fraction captured an unusually long but non-representative read, or where neighbouring-gene contamination was not fully excluded by our segmentation filter despite the safeguards described in Methods. We have quantified that cost against TIF-seq, which took no part in construction. The final boundary exceeds the major covering isoform by more than 20 nt for 54% of genes at the 5′ end and 47,9% at the 3′ end. Most of that excess falls within an isoform TIF-seq also observed, leaving 7.2% and 3.1% of genes beyond anything the benchmark recorded. We tested this directly rather than assume it. Running the released method separately on the two independent physiological conditions behind the six construction libraries, before either is merged with Nagalakshmi et al., the resulting DRS-only calls agree exactly for 21.5% of genes at the 5′ end and 42.9% at the 3′ end, and agree within 20 nt for 67.7% and 77.9% (Supplementary Table S6). Condition therefore has a real, not negligible, effect on the DRS-derived boundary for a meaningful share of genes. We read this as support for the merge rule’s design rather than a threat to it: if two genuine physiological conditions can each supply real evidence for a different UTR length at the same gene, then no single condition’s call is uniquely correct, and retaining the fullest support seen across the conditions available to us is the more defensible choice than arbitrarily preferring one.

### Outlook and applications

For the practising yeast researcher, the immediate value of this resource is that more of the data is placed. Against the prior reference, the released annotation recovers 0.77% more direct RNA reads and reduces soft clipping at transcript ends, and it does so without any change to existing workflows. These are alignment level improvements and we describe them as such. At the level of abundance, the two references give closely concordant per gene estimates (Spearman ρ = 0.978), so the practical effect of adopting this reference is a modest gain in the fraction of reads placed rather than a systematic change in abundance estimates. We have not benchmarked differential expression calls, because the libraries available to us do not carry a replicate structure that would support such a comparison. Beyond quantification, the recovered and extended UTRs open specific lines of enquiry that an ORF-only or UTR-incomplete reference cannot support: the 3′ UTRs underpin studies of alternative polyadenylation, mRNA stability (Tudek et al. 2021), and the *cis*-regulatory elements bound by RNA-binding proteins, while the 5′ UTRs (with the sensitivity caveat noted above) inform analyses of translation initiation and upstream open reading frames. Because the pipeline is released alongside the annotation, the same approach can be applied to other *S. cerevisiae* strains, to defined growth or stress conditions, or to non-conventional yeasts and other compact genomes, yielding condition- or strain-specific references where a single static annotation is inadequate.

Several extensions follow naturally from this work. The conservative merge could be complemented by a fully data-driven re-annotation permitted to shorten over-extended boundaries. Our comparison against transcript-isoform sequencing TIF-seq (Pelechano et al. 2013), which delimits both transcript ends simultaneously and is fully independent of our annotation, quantifies the scale of that residual. Truncation is the minority case, affecting 5.2% of genes at the 5′ end and 7.2% at the 3′ end, whereas the final boundary exceeds the major TIF-seq isoform by more than 20 nt for 54.0% of genes at the 5′ end and 47.9% at the 3′ end (Supplementary Figs S2 and S6). The genes whose boundaries lie beyond every isoform the benchmark recorded, 6.7% at the 5′ end and 2.7% at the 3′ end, are the cases such a re-annotation would need to address first. As Nanopore read lengths and direct-RNA chemistry continue to improve, the 5′ resolution that currently limits 5′ UTR calls will tighten, allowing the reference to be refreshed simply by re-running the released pipeline on new data. More broadly, integrating this UTR-focused resource with isoform-discovery efforts such as UNAGI would combine accurate boundary annotation with comprehensive transcript cataloguing, moving the field towards a single, continuously updatable yeast transcriptome reference. Finally, because DRS preserves native RNA base modifications, the same reads can additionally be mined for epitranscriptomic signal alongside boundary annotation (Leger et al. 2021), further increasing the information yield of a single DRS dataset.

## Conclusion

We present a merged *S. cerevisiae* transcriptome reference that extends the Nagalakshmi et al. UTR annotation (Nagalakshmi et al. 2008) using DRS, while guaranteeing, by construction and validated independently, that no existing annotation is shortened in the process. The resource fills roughly half of Nagalakshmi’s UTR annotation gaps, improves mapping rate, specificity, and soft-clip burden relative to both the existing reference and naive alternatives on data independent of its construction, and is provided together with its full reproducible pipeline so that it can be applied to new conditions, strains, or sequencing runs as long-read yeast transcriptomics continues to mature. We anticipate this resource will be most immediately useful as a direct substitute reference for existing yeast RNA-seq pipelines, with the underlying pipeline available for groups wishing to extend or refine the annotation further.

## Supplementary Material

foag052_Supplemental_Files

## Data Availability

The transcript FASTA, the GFF3 annotation, and the merged UTR table are deposited at Zenodo under DOI 10.5281/zenodo.20828039 and are also provided as Supplementary data. Both the in-house DRS-based reference and the Nagalakshmi-based comparison reference are released as FASTA files with MD5 checksums f5b10c83168122536a8eed5efe3006cf and 0edf6ab57c48eba0f29344bd75e6695b, respectively, so that users can confirm they hold the exact released sequences. The construction pipeline, the validation harness, and the scripts that generate the figures in this manuscript are available at https://github.com/Arnaroo/Yeast_Annotation. The unmerged DRS boundary calls, i.e. the lengths obtained from the sequencing data before the merge with the Nagalakshmi et al. annotation, are released alongside the merged table as Data/Annotation/DRS_UTR_corrected.tsv, so that users who prefer the data-driven calls without the conservative merge can take them directly. The sequencing data used to build the reference are publicly accessible at the NCBI Sequence Read Archive under BioProject PRJNA1022817, with the eight individual run accessions listed in Supplementary Table S1. The long-read validation data are accessible at the same archive under experiment accession SRX28193073, run SRR32918129. The short-read validation data are accessible at the DDBJ under BioProject PRJDB8048, runs DRR170478 and DRR170479. The transcript-isoform sequencing boundaries used as an independent benchmark were derived from the TIF-seq isoform table of Pelechano et al. (2013), GEO accession GSE39128, by the script released with this work. The UNAGI boundaries used as the second independent benchmark were taken from supplementary files 5 and 6 of Al Kadi et al. (2020), doi 10.1007/s10142-020-00732–1, and processed by the script released with this work.
