# Supplementary material for: A refined Saccharomyces cerevisiae reference transcriptome from direct RNA sequencing, with a reusable pipeline for UTR annotation updates
Source: FEMS Yeast Res. 2026 Sep 23;26:foag052. doi: 10.1093/femsyr/foag052 (PMC13637595; doi:10.1093/femsyr/foag052)
Supplement: Supplementary file 1 [file foag052_supplemental_files.zip › SuppFig1.pdf]

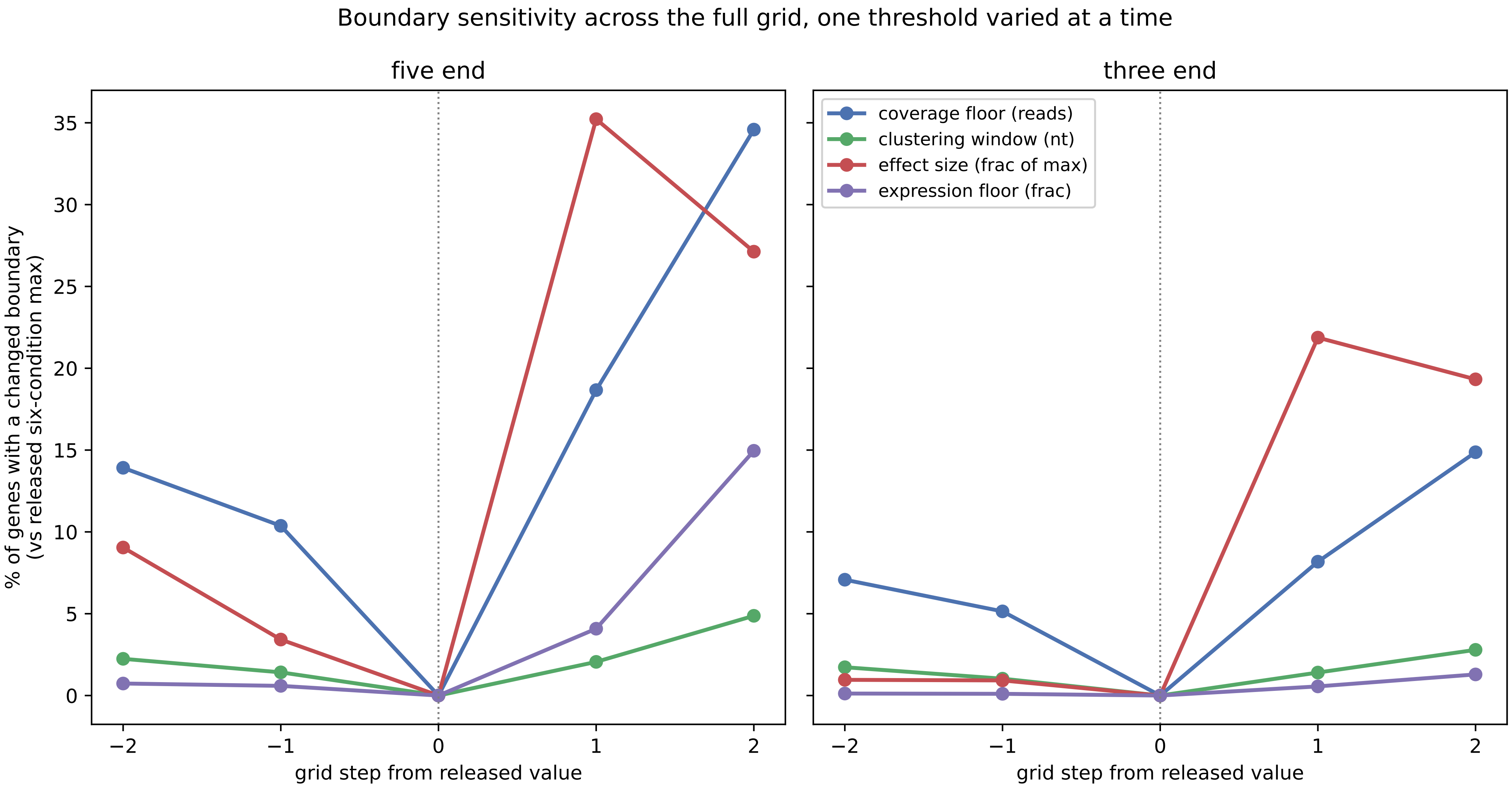

coverage floor (reads): 5, 10, 20, 40, 80 (released 20)
clustering window (nt): 10, 20, 40, 80, 160 (released 40)
effect size (frac of max): 0.025, 0.05, 0.1, 0.2, 0.35 (released 0.1)
expression floor (frac): 0.01, 0.025, 0.05, 0.1, 0.2 (released 0.05)
