# Supplementary material for: A refined Saccharomyces cerevisiae reference transcriptome from direct RNA sequencing, with a reusable pipeline for UTR annotation updates
Source: FEMS Yeast Res. 2026 Sep 23;26:foag052. doi: 10.1093/femsyr/foag052 (PMC13637595; doi:10.1093/femsyr/foag052)
Supplement: Supplementary file 1 [file foag052_supplemental_files.zip › SuppFig2.pdf]

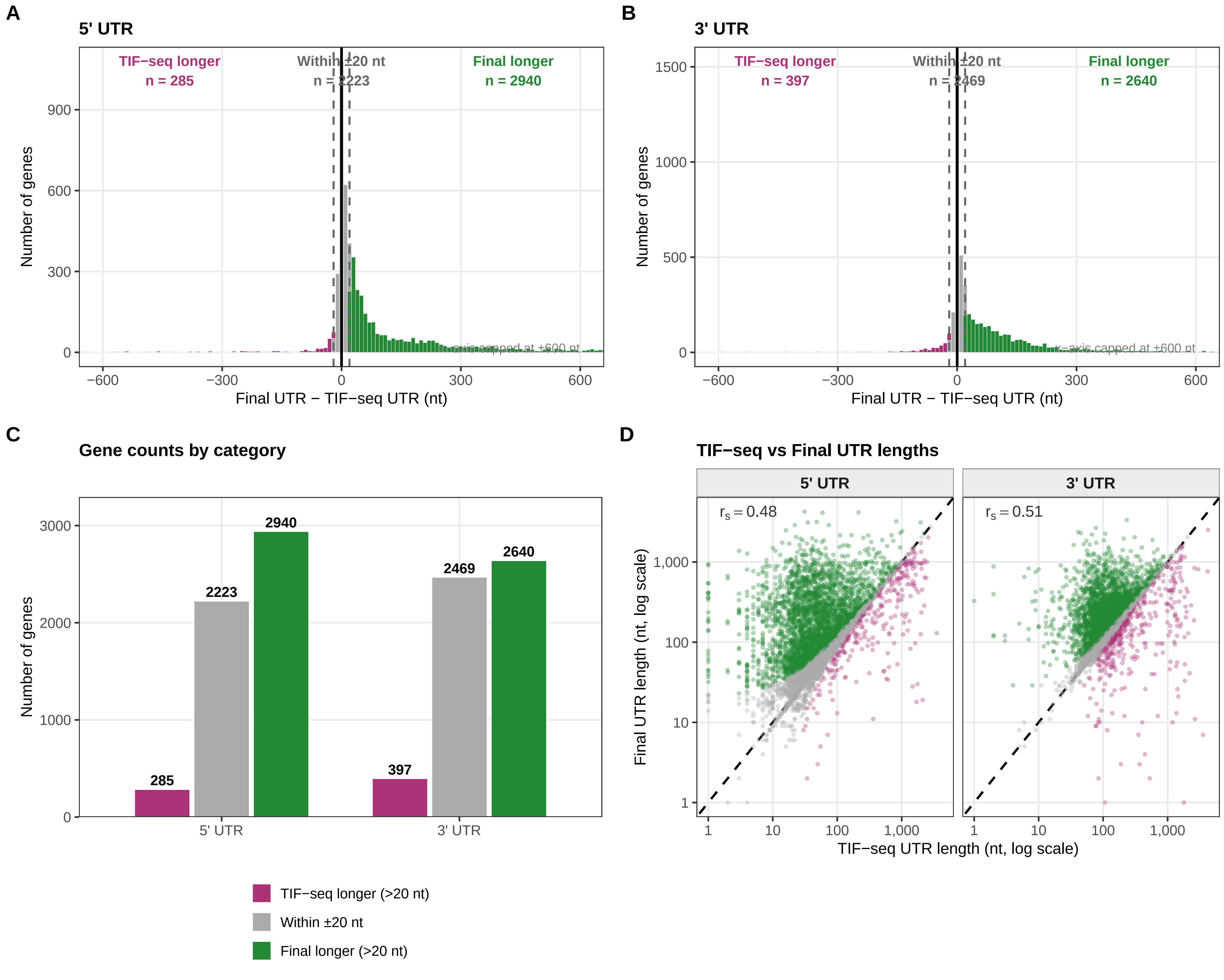

threshold excludes differences plausibly attributable to noise. Histogram x−axes capped at ±600 nt. Dashed line in panel D = y = x. Spearman r_s 5' = 0.48, 3' = 0.51. Purple: TIF−seq longer; green: final annotation longer.
