# Supplementary material for: A refined Saccharomyces cerevisiae reference transcriptome from direct RNA sequencing, with a reusable pipeline for UTR annotation updates
Source: FEMS Yeast Res. 2026 Sep 23;26:foag052. doi: 10.1093/femsyr/foag052 (PMC13637595; doi:10.1093/femsyr/foag052)
Supplement: Supplementary file 1 [file foag052_supplemental_files.zip › SuppFig3.pdf]

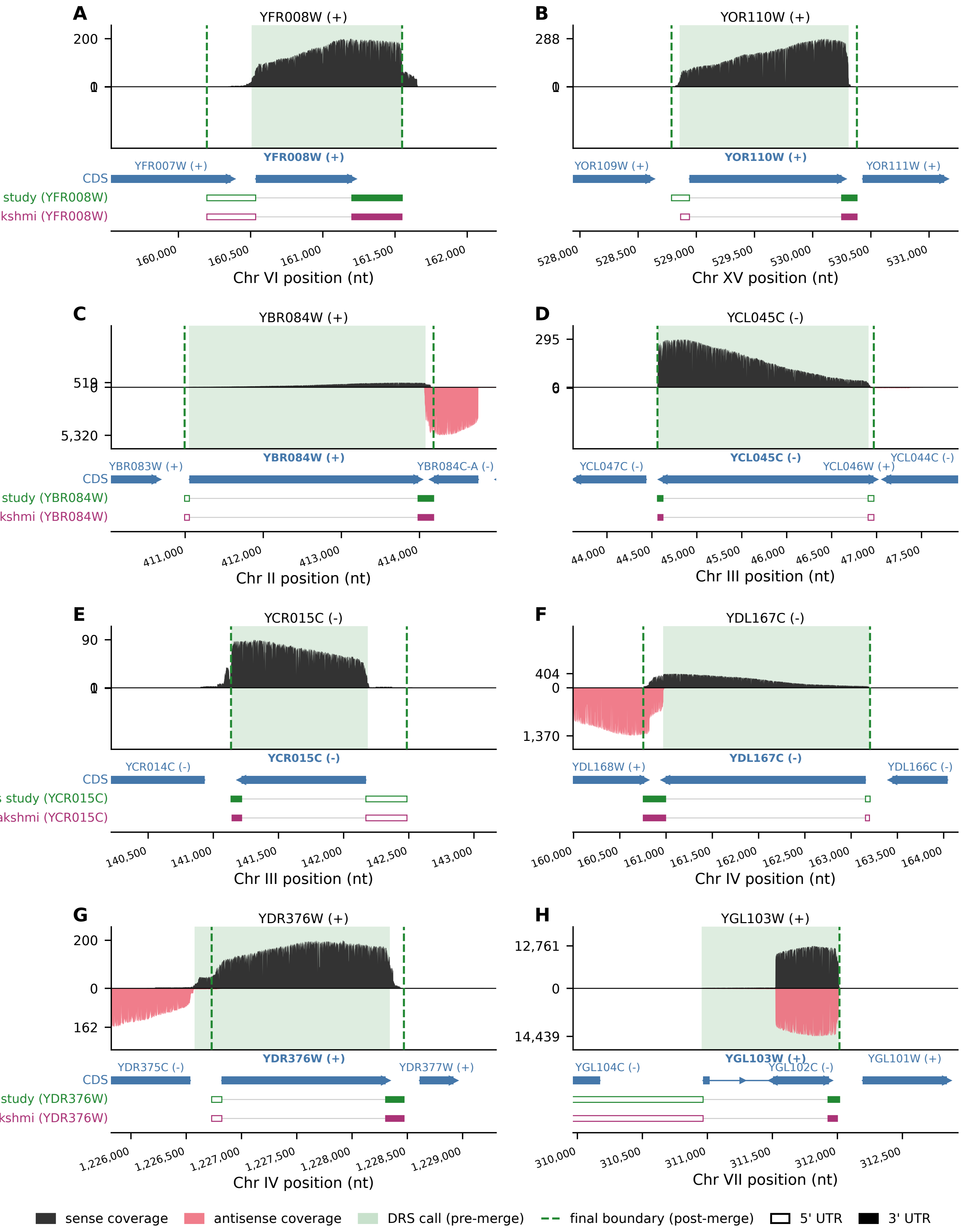
A
YFR008W (+)
200
0
YFR007W (+)
YFR008W (+)
CDS
study (YFR008W)
kshmi (YFR008W)
160,000
160,500
161,000
161,500
162,000
Chr VI position (nt)
B
YOR110W (+)
288
0
YOR109W (+)
YOR110W (+)
YOR111W (+)
528,000
528,500
529,000
529,500
530,000
530,500
531,000
Chr XV position (nt)
C
YBR084W (+)
519
0
5,320
YBR083W (+)
YBR084W (+)
YBR084C-A (-)
CDS
study (YBR084W)
kshmi (YBR084W)
411,000
412,000
413,000
414,000
Chr II position (nt)
D
YCL045C (-)
295
0
YCL047C (-)
YCL045C (-)
YCL046W (+)
YCL044C (-)
44,000
44,500
45,000
45,500
46,000
46,500
47,000
47,500
Chr III position (nt)
E
YCR015C (-)
90
0
YCR014C (-)
YCR015C (-)
CDS
s study (YCR015C)
akshmi (YCR015C)
140,500
141,000
141,500
142,000
142,500
143,000
Chr III position (nt)
F
YDL167C (-)
404
0
1,370
YDL168W (+)
YDL167C (-)
YDL166C (-)
160,000
160,500
161,000
161,500
162,000
162,500
163,000
163,500
164,000
Chr IV position (nt)
G
YDR376W (+)
200
0
162
YDR375C (-)
YDR376W (+)
YDR377W (+)
CDS
study (YDR376W)
kshmi (YDR376W)
1,226,000
1,226,500
1,227,000
1,227,500
1,228,000
1,228,500
1,229,000
Chr IV position (nt)
H
YGL103W (+)
12,761
0
14,439
YGL104C (-)
YGL103W (+)
YGL102C (-)
YGL101W (+)
310,000
310,500
311,000
311,500
312,000
312,500
Chr VII position (nt)
sense coverage
antisense coverage
DRS call (pre-merge)
final boundary (post-merge)
5' UTR
3' UTR
