# Supplementary material for: A refined Saccharomyces cerevisiae reference transcriptome from direct RNA sequencing, with a reusable pipeline for UTR annotation updates
Source: FEMS Yeast Res. 2026 Sep 23;26:foag052. doi: 10.1093/femsyr/foag052 (PMC13637595; doi:10.1093/femsyr/foag052)
Supplement: Supplementary file 1 [file foag052_supplemental_files.zip › SuppFig4.pdf]

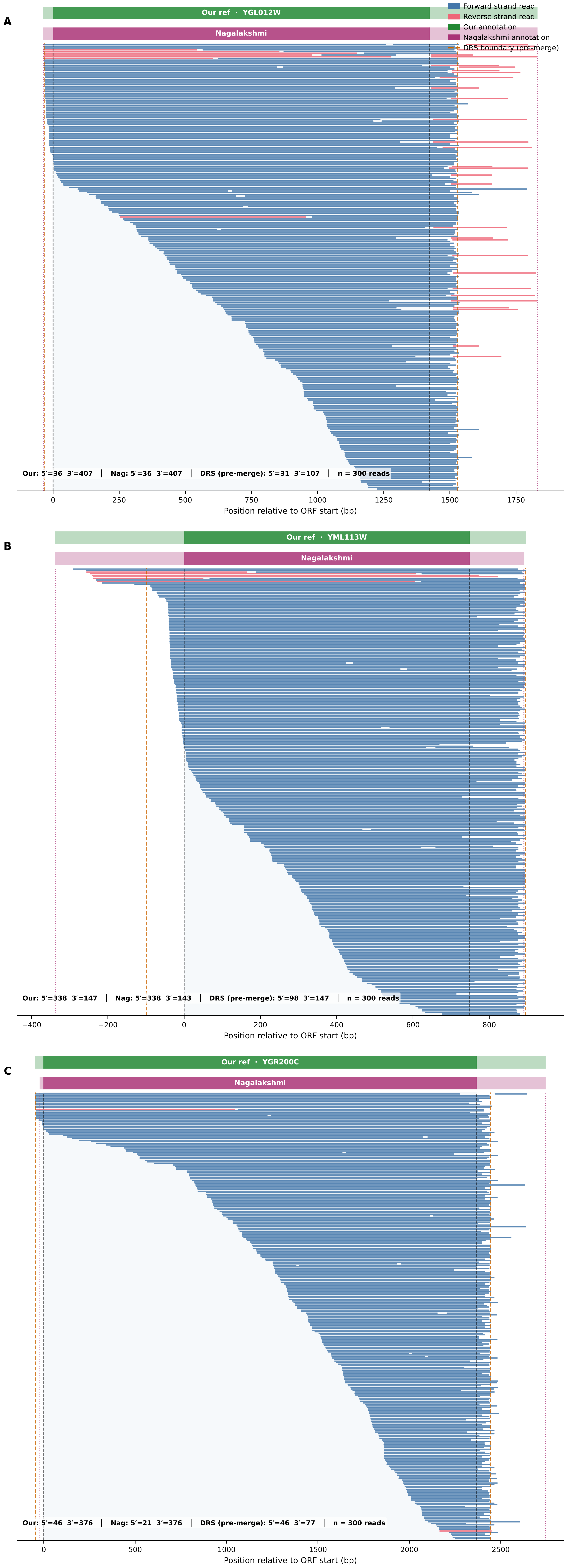
A
Our ref · YGL012W
Nagalakshmi
Forward strand read
Reverse strand read
Our annotation
Nagalakshmi annotation
DRS boundary (pre-merge)
Our: 5′=36 3′=407 │ Nag: 5′=36 3′=407 │ DRS (pre-merge): 5′=31 3′=107 │ n = 300 reads
0 250 500 750 1000 1250 1500 1750
Position relative to ORF start (bp)
B
Our ref · YML113W
Nagalakshmi
Our: 5′=338 3′=147 │ Nag: 5′=338 3′=143 │ DRS (pre-merge): 5′=98 3′=147 │ n = 300 reads
−400 −200 0 200 400 600 800
Position relative to ORF start (bp)
C
Our ref · YGR200C
Nagalakshmi
Our: 5′=46 3′=376 │ Nag: 5′=21 3′=376 │ DRS (pre-merge): 5′=46 3′=77 │ n = 300 reads
0 500 1000 1500 2000 2500
Position relative to ORF start (bp)
