# Supplementary material for: A refined Saccharomyces cerevisiae reference transcriptome from direct RNA sequencing, with a reusable pipeline for UTR annotation updates
Source: FEMS Yeast Res. 2026 Sep 23;26:foag052. doi: 10.1093/femsyr/foag052 (PMC13637595; doi:10.1093/femsyr/foag052)
Supplement: Supplementary file 1 [file foag052_supplemental_files.zip › SuppFig5.pdf]

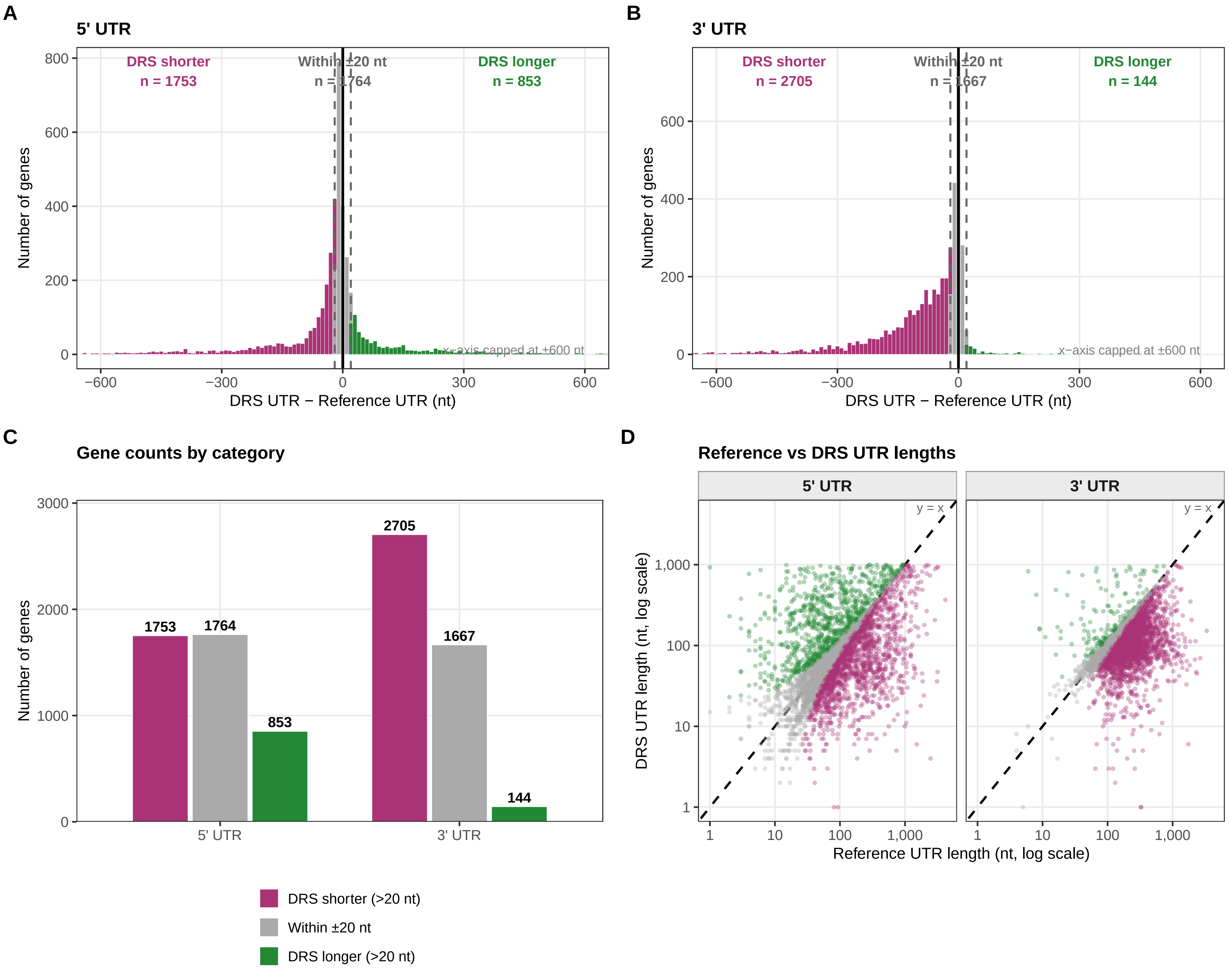

e values fall outside the displayed range. Dashed black line in panel D = y = x (equal UTR lengths). Purple: Nagalakshmi annotation longer (retained by merge). Green: DRS annotation longer (DRS call retained by merge).
