# Supplementary material for: A refined Saccharomyces cerevisiae reference transcriptome from direct RNA sequencing, with a reusable pipeline for UTR annotation updates
Source: FEMS Yeast Res. 2026 Sep 23;26:foag052. doi: 10.1093/femsyr/foag052 (PMC13637595; doi:10.1093/femsyr/foag052)
Supplement: Supplementary file 1 [file foag052_supplemental_files.zip › SuppFig6.pdf]

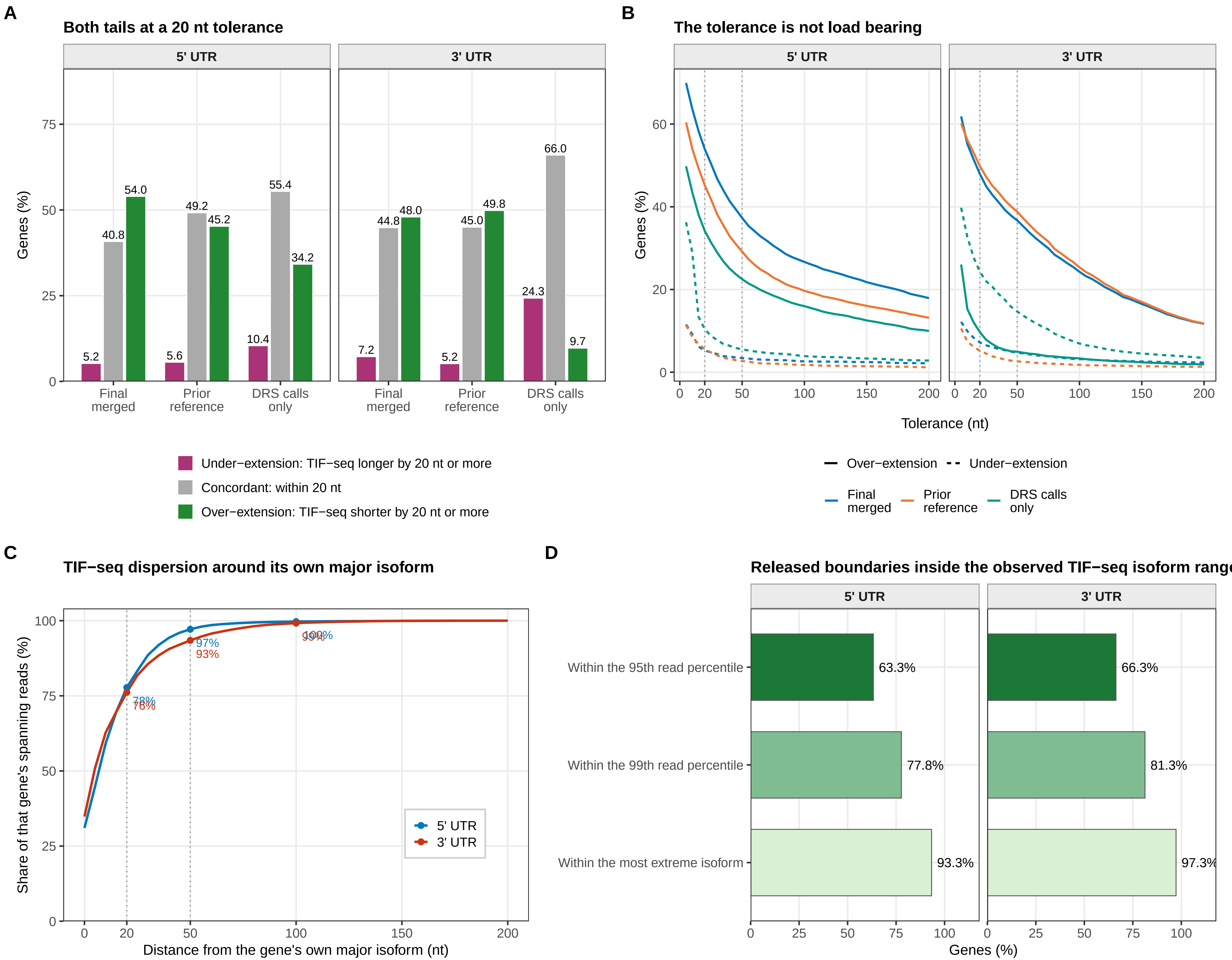

Over−extension and under−extension of transcript boundaries relative to the independent TIF−seq benchmark (Pelechano et al., 2013), reported explicitly in both directions. Differences are signed, annotation minus TIF−seq, over genes where both report a non−zero UTR (final annotation: n = 5448 at the 5' end, n = 5506 at the 3' end). (A) The prior reference over−extends relative to TIF−seq at almost the same rate as the merged annotation, while the raw DRS calls agree with TIF−seq most closely of the three. (B) Both rates vary smoothly with the tolerance and the ordering of the three annotations does not change. (C) The benchmark's own dispersion: a 20 nt tolerance is narrower than the interquartile spread of TIF−seq ends within a single gene. (D) Most released boundaries fall inside the range of ends TIF−seq observed for that gene; 6.7% at the 5' end and 2.7% at the 3' end exceed every isoform TIF−seq recorded.
