# Supplementary material for: A refined Saccharomyces cerevisiae reference transcriptome from direct RNA sequencing, with a reusable pipeline for UTR annotation updates
Source: FEMS Yeast Res. 2026 Sep 23;26:foag052. doi: 10.1093/femsyr/foag052 (PMC13637595; doi:10.1093/femsyr/foag052)
Supplement: Supplementary file 1 [file foag052_supplemental_files.zip › SuppFig7.pdf]

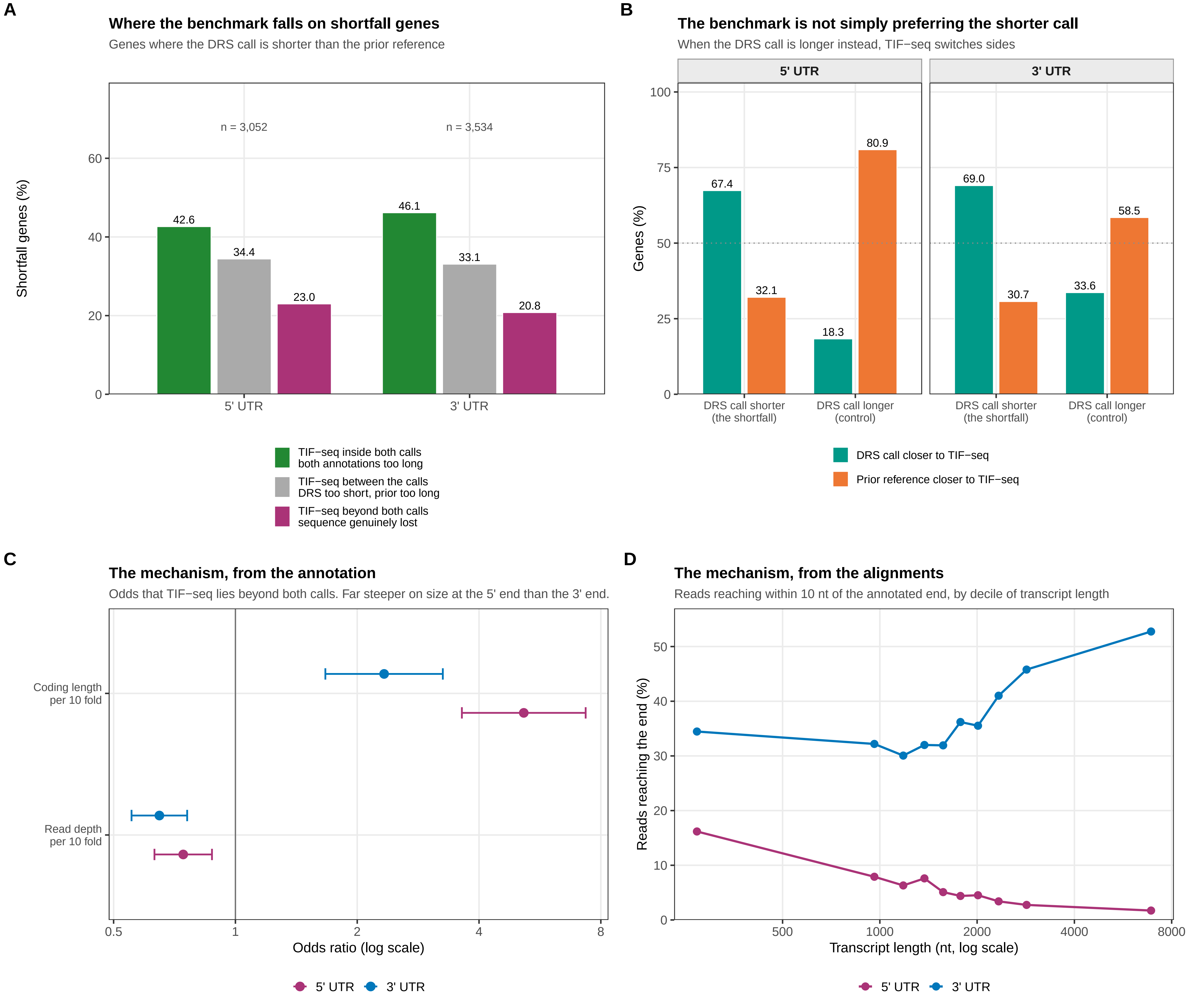

Genes where the DRS−derived UTR is shorter than the prior reference, tested against the independent TIF−seq benchmark (Pelechano et al., 2013), which took no part in constructing the annotation (n = 3,052 at the 5' end, n = 3,534 at the 3'
