# Supplementary material for: A refined Saccharomyces cerevisiae reference transcriptome from direct RNA sequencing, with a reusable pipeline for UTR annotation updates
Source: FEMS Yeast Res. 2026 Sep 23;26:foag052. doi: 10.1093/femsyr/foag052 (PMC13637595; doi:10.1093/femsyr/foag052)
Supplement: Supplementary file 1 [file foag052_supplemental_files.zip › SuppFig8.pdf]

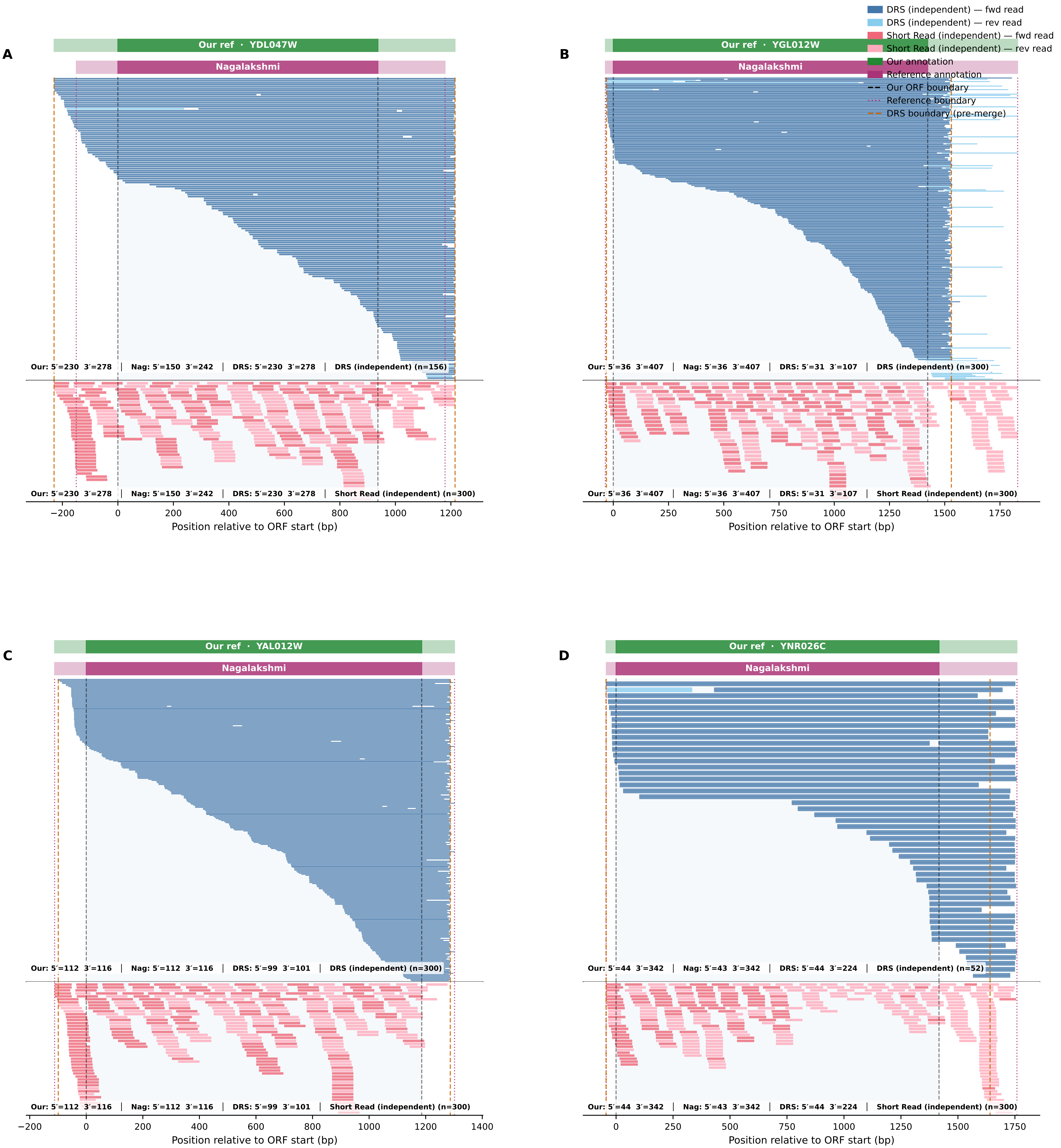

DRS (independent) — fwd read
DRS (independent) — rev read
Short Read (independent) — fwd read
Short Read (independent) — rev read
Our annotation
Reference annotation
Our ORF boundary
Reference boundary
DRS boundary (pre-merge)
A
Our ref · YDL047W
Nagalakshmi
Our: 5′=230 3′=278 | Nag: 5′=150 3′=242 | DRS: 5′=230 3′=278 | DRS (independent) (n=156)
Our: 5′=230 3′=278 | Nag: 5′=150 3′=242 | DRS: 5′=230 3′=278 | Short Read (independent) (n=300)
Position relative to ORF start (bp)
B
Our ref · YGL012W
Nagalakshmi
Our: 5′=36 3′=407 | Nag: 5′=36 3′=407 | DRS: 5′=31 3′=107 | DRS (independent) (n=300)
Our: 5′=36 3′=407 | Nag: 5′=36 3′=407 | DRS: 5′=31 3′=107 | Short Read (independent) (n=300)
Position relative to ORF start (bp)
C
Our ref · YAL012W
Nagalakshmi
Our: 5′=112 3′=116 | Nag: 5′=112 3′=116 | DRS: 5′=99 3′=101 | DRS (independent) (n=300)
Our: 5′=112 3′=116 | Nag: 5′=112 3′=116 | DRS: 5′=99 3′=101 | Short Read (independent) (n=300)
Position relative to ORF start (bp)
D
Our ref · YNR026C
Nagalakshmi
Our: 5′=44 3′=342 | Nag: 5′=43 3′=342 | DRS: 5′=44 3′=224 | DRS (independent) (n=52)
Our: 5′=44 3′=342 | Nag: 5′=43 3′=342 | DRS: 5′=44 3′=224 | Short Read (independent) (n=300)
Position relative to ORF start (bp)
