# Supplementary material for: A refined Saccharomyces cerevisiae reference transcriptome from direct RNA sequencing, with a reusable pipeline for UTR annotation updates
Source: FEMS Yeast Res. 2026 Sep 23;26:foag052. doi: 10.1093/femsyr/foag052 (PMC13637595; doi:10.1093/femsyr/foag052)
Supplement: Supplementary file 1 [file foag052_supplemental_files.zip › SuppFig9.pdf]

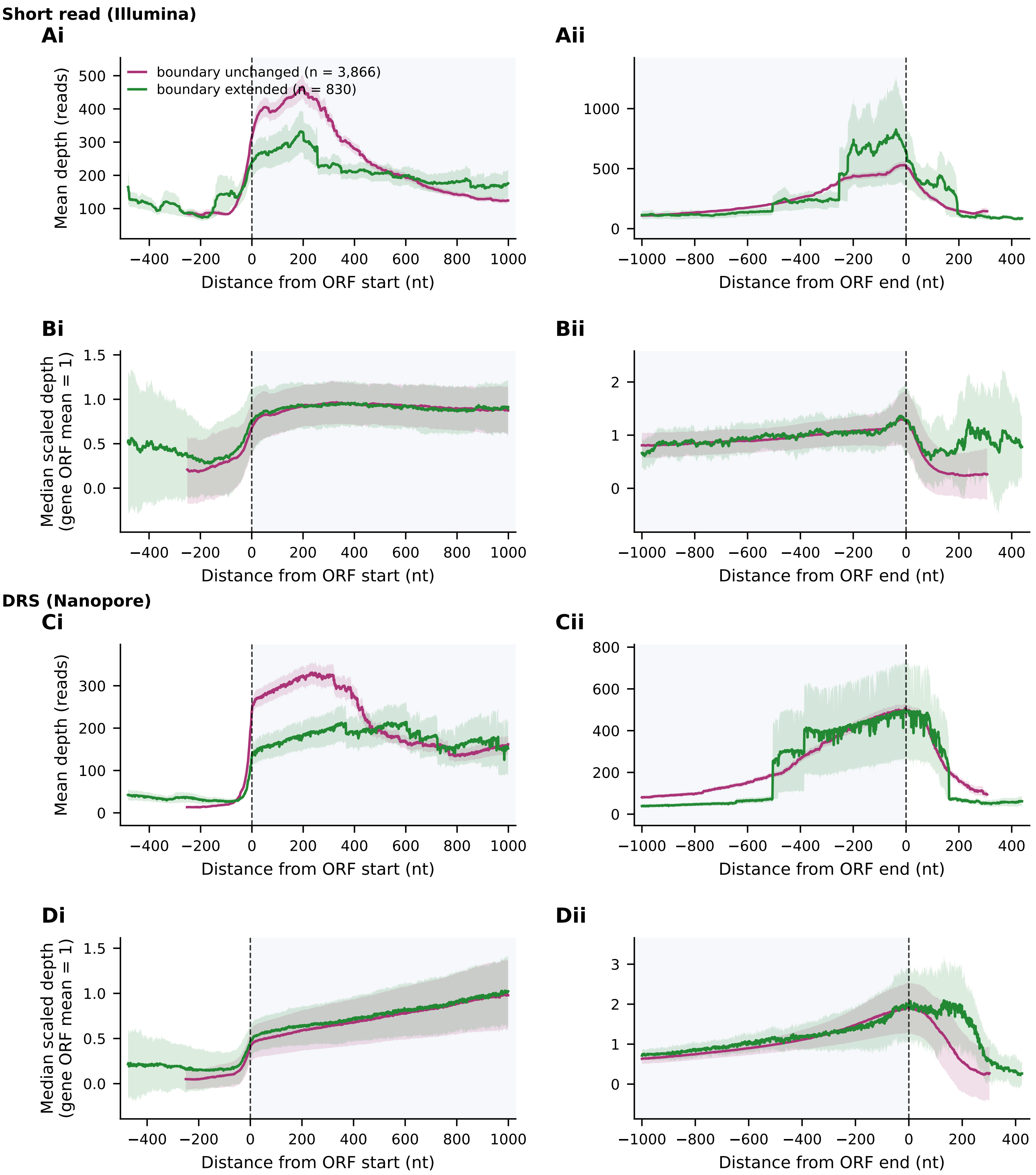

Short read (Illumina)
Ai
Aii
boundary unchanged (n = 3,866)
boundary extended (n = 830)
Mean depth (reads)
Distance from ORF start (nt)
Distance from ORF end (nt)
Bi
Bii
Median scaled depth (gene ORF mean = 1)
DRS (Nanopore)
Ci
Cii
Di
Dii
