# Supplementary material for: A refined Saccharomyces cerevisiae reference transcriptome from direct RNA sequencing, with a reusable pipeline for UTR annotation updates
Source: FEMS Yeast Res. 2026 Sep 23;26:foag052. doi: 10.1093/femsyr/foag052 (PMC13637595; doi:10.1093/femsyr/foag052)
Supplement: Supplementary file 1 [file foag052_supplemental_files.zip › Supplementary_Tables.pdf]

Supplementary Tables

A refined *Saccharomyces cerevisiae* reference transcriptome from Direct RNA Sequencing, with a reusable pipeline for UTR annotation updates

Rossini, Cleynen and Shirokikh. Manuscript FEMSYR-26-06-0101. Supplementary Figures S1 to S9 are supplied separately. Every table below is generated from the analysis outputs by Analysis/scripts/s08b_supp_tables.py, which fails rather than writes if a value it quotes stops matching the manuscript.

Supplementary Table S1. SRA run accessions for the construction-stage direct RNA libraries.

Supplementary Table S1. SRA run accessions for the construction-stage direct RNA libraries. FASTQ run accessions under BioProject PRJNA1022817 (SRA study SRP464030) for the construction-stage direct RNA libraries, retrieved from the NCBI SRA Run Selector. Each row is one condition/replicate combination; the BAM (aligned) and FAST5 (raw signal) runs deposited alongside each FASTQ are omitted here to avoid confusion with the BAMs generated during this analysis, and can be found under the same BioProject if needed. PS = heavy polysome (tetrasome and larger) fraction, RDT = light polysome (ribosome, disome, trisome) fraction; NS = non-starved, S10 = 10 minute glucose-starved. PROVISIONAL: only two replicates (A, B) per condition are currently deposited under this BioProject; this is under discussion with collaborators and this table may need revision. Produced by s08b_supp_tables.py from inputs/sra/construction_library_runs.tsv, retrieved from the NCBI SRA Run Selector.

condition	replicate	SRA run	SRA experiment	sample attribute
NS_PS	A	SRR26244937	SRX21955197	PS fraction of non-starved cells replicate A
NS_PS	B	SRR26244950	SRX21955184	PS fraction of non-starved cells replicate B
NS_RDT	A	SRR26244953	SRX21955181	RDT fraction of non-starved cells replicate A
NS_RDT	B	SRR26244955	SRX21955179	RDT fraction of non-starved cells replicate B
S10_PS	A	SRR26244959	SRX21955175	PS fraction of starved cells replicate A
S10_PS	B	SRR26244941	SRX21955193	PS fraction of starved cells replicate B
S10_RDT	A	SRR26244963	SRX21955171	RDT fraction of starved cells replicate A
S10_RDT	B	SRR26244946	SRX21955188	RDT fraction of starved cells replicate B

Supplementary Table S2. Number reconciliation across pipeline stages.

Supplementary Table S2. Number reconciliation across pipeline stages. Gene and transcript counts at every stage of the pipeline, with the filter that produces each count, the file it is read from and the code that writes it. Stage 00 is the S288C R64-4-1 gene model backbone. Stage 05, the merged UTR table, is larger than the backbone because the merge is a union of the DRS calls and the prior reference rather than an intersection. Stage 06, the number of distributed transcript sequences, is fixed by the backbone and not by the UTR table, which is why it is identical for our reference and for the prior reference. Stage 07 is the independent TIF-seq boundary set, which took no part in construction and is used only for validation. Produced by s01_number_flow.py.

Stage	Description	Count	Filter applied	Source file	Producing script
00	S288C R64-4-1 gene models, backbone GFF3 gene features	6,607	none	gene_models_backbone_with_UTR_slots.gff3	backbone supplied with the pipeline
01	Gene models carrying a real CDS in the backbone	6,704	has a non UTR CDS feature	gene_models_backbone_with_UTR_slots.gff3	counted here
02	Gene models emitted as transcripts by gffread -M -A	6,767	gffread builds one record per mRNA parent	Final_w.fa	04_build_utr_reference.py
03	Genes with a Nagalakshmi UTR table row	6,607	present in Nagalakshmi et al. 2008 table	Nagalakshmi_UTR.csv	external reference
03b	Nagalakshmi rows with at least one non NA boundary	4,818	non NA 5′ or 3′ value	Nagalakshmi_UTR.csv	counted here
04	Genes surviving DRS segmentation in at least one condition	5,770	max coverage > 20 reads AND a selected breakpoint inside [1000, n-1000]	DRS_UTR_corrected.tsv	02_transcripts_segmentation.R L46 L66 L182
05	Final merged UTR table, DRS union Nagalakshmi	6,695	full_join, union not intersection	final_utr.tsv	03_utr_table.R L200
06	Transcript sequences in the built reference	6,767	identical to stage 02, set by the backbone not by the UTR table	Final_w.fa	04_build_utr_reference.py
07	Genes with an independent TIF-seq boundary	6,579	present in Pelechano et al. table	Pelechano_UTR.csv	external, not used in construction

Supplementary Table S3. Boundary calls, UTR lengths and TIF-seq agreement by SGD ORF class.

Supplementary Table S3. Boundary calls, UTR lengths and TIF-seq agreement by SGD ORF class. Genes are classified by the SGD R64-4-1 qualifier as Verified, Uncharacterized or Dubious. For each class the table gives the number of genes, the ORF span, the fraction receiving a boundary call from DRS and from the prior reference, median UTR lengths, and agreement with the independent TIF-seq boundaries of Pelechano et al. (2013), which took no part in construction. Agreement is the signed difference between our boundary and the TIF-seq boundary, ours minus theirs, at a tolerance of 20 nt: under-called means our UTR is shorter by 20 nt or more, over-called means longer by 20 nt or more, and concordant means the two agree to within 20 nt. The three percentages are reported together so that the direction of every disagreement is visible. The DRS call rate differs strongly across the three classes (chi-square, 2 df, p below 10^{-300}, Cramér V = 0.58). Because Dubious ORFs are also short, the comparison was repeated with ORF span held constant by stratifying on span decile, which gives a Mantel-Haenszel odds ratio for a DRS call of 0.09 for Dubious against Verified. Produced by s03a_orf_class.py.

Measure	Verified	Uncharacterized	Dubious
Genes in class	5,255	668	684
ORF span, median (nt)	1,278	483	348
ORF span, Q1 (nt)	801	306	303
ORF span, Q3 (nt)	1,932	1,029	414.8
Genes with a DRS boundary call, n	4,936	408	338
Genes with a DRS boundary call, %	93.9	61.1	49.4
Genes with a prior reference call, n	4,315	356	147
Genes with a prior reference call, %	82.1	53.3	21.5
Merged 5′ UTR, median (nt)	97	106	86
Merged 3′ UTR, median (nt)	160	131	20.5
DRS 5′ UTR, median (nt)	67	160	457.5
DRS 3′ UTR, median (nt)	108	126	239.5
Extended beyond the prior reference at 5′, %	28.3	33.2	36.7
Extended beyond the prior reference at 3′, %	19.5	18.9	18.4
TIF-seq 5′, genes compared	4,780	428	240
TIF-seq 5′, median signed difference (nt)	25	34.5	19
TIF-seq 5′, concordant within 20 nt, %	42.4	32.0	24.6
TIF-seq 5′, under-called by 20 nt or more, %	3.8	10.0	25.8
TIF-seq 5′, over-called by 20 nt or more, %	53.8	57.9	49.6
TIF-seq 3′, genes compared	4,837	431	238
TIF-seq 3′, median signed difference (nt)	18	15	-1
TIF-seq 3′, concordant within 20 nt, %	45.6	41.3	34.9
TIF-seq 3′, under-called by 20 nt or more, %	5.7	11.1	29.8
TIF-seq 3′, over-called by 20 nt or more, %	48.6	47.6	35.3

Supplementary Table S4. Gene pairs on the same strand whose extended span reaches into the partner's ORF.

Supplementary Table S4. Gene pairs on the same strand whose extended span reaches into the partner's ORF. Extending boundaries in a genome this compact necessarily creates overlaps. Relative to the prior reference, 605 gene pairs newly overlap. Listed here are the 102 that matter for stranded quantification: pairs on the same strand whose newly extended span reaches into the body of the partner's open reading frame. They involve 200 genes, 2.96% of the 6,767 distributed transcripts. All 102 are tandem pairs, since a same strand overlap cannot be convergent or divergent. Overlap is the length of the overlapping interval; overlap into ORF is the part of it that falls inside the partner's coding sequence. Users who need strictly disjoint intervals can exclude these pairs. Rows are sorted by overlap into ORF, longest first. Produced by s03b_neighbour_overlap.py.

Gene A	Gene B	Class A	Class B	Strand	Overlap (nt)	Overlap into ORF (nt)	Both Verified
YKL020C	YKL021C	Verified	Verified	-	987	914	yes
YBL062W	YBL063W	Dubious	Verified	+	1,025	904	no
YNL170W	YNL172W	Dubious	Verified	+	1,188	868	no
YKL021C	YKL022C	Verified	Verified	-	919	807	yes
YOR192C-B	YOR192C-C		Uncharacterized	-	784	784	no
YKR103W	YKR104W	Verified	Verified	+	782	782	yes
YNR065C	YNR066C	Uncharacterized	Uncharacterized	-	760	760	no
YLR070C	YLR071C	Verified	Verified	-	882	737	yes
YBL095W	YBL097W	Uncharacterized	Verified	+	725	725	no
YMR063W	YMR064W	Verified	Verified	+	720	720	yes
YNR068C	YNR069C	Uncharacterized	Verified	-	1,619	698	no
YNL261W	YNL262W	Verified	Verified	+	859	687	yes
YMR147W	YMR149W	Uncharacterized	Verified	+	860	684	no
YEL019C	YEL020C	Verified	Verified	-	791	637	yes
YGL223C	YGL224C	Verified	Verified	-	770	628	yes
YBR136W	YBR137W	Verified	Verified	+	1,550	580	yes
YPL193W	YPL194W	Verified	Verified	+	814	578	yes
YLR030W	YLR031W	Uncharacterized	Uncharacterized	+	561	561	no
YLR115W	YLR116W	Verified	Verified	+	683	533	yes
YHR165C	YHR166C	Verified	Verified	-	686	530	yes
YHR035W	YHR036W	Verified	Verified	+	723	521	yes
YGL258W-A	YGL259W	Uncharacterized	Uncharacterized	+	498	498	no
YBR101C	YBR102C	Verified	Verified	-	607	474	yes
YCR005C	YCR006C	Verified	Uncharacterized	-	474	474	no
YDL037C	YDL039C	Verified	Verified	-	473	473	yes
YNL203C	YNL204C	Dubious	Verified	-	469	469	no
YMR148W	YMR149W	Verified	Verified	+	860	447	yes
YDL220C	YDL222C	Verified	Verified	-	827	445	yes
YAL020C	YAL021C	Verified	Verified	-	587	442	yes
YML002W	YML003W	Uncharacterized	Uncharacterized	+	1,048	436	no
YML037C	YML038C	Uncharacterized	Verified	-	431	431	no
YML075C	YML076C	Verified	Verified	-	538	427	yes

Gene A	Gene B	Class A	Class B	Strand	Overlap (nt)	Overlap into ORF (nt)	Both Verified
YBL048W	YBL049W	Uncharacterized	Verified	+	886	417	no
YJL050W	YJL051W	Verified	Verified	+	625	410	yes
YPL191C	YPL192C	Verified	Verified	-	960	405	yes
YCR106W	YCR107W	Verified	Verified	+	399	399	yes
YNL053W	YNL054W	Verified	Verified	+	670	395	yes
YGR093W	YGR094W	Verified	Verified	+	512	388	yes
YOR026W	YOR027W	Verified	Verified	+	583	381	yes
YLR154W-C	YLR154W-E	Verified	Dubious	+	1,050	375	no
YPR173C	YPR174C	Verified	Verified	-	519	363	yes
YLR368W	YLR369W	Verified	Verified	+	409	363	yes
YBR140C	YBR141C	Verified	Verified	-	517	350	yes
YOR295W	YOR296W	Verified	Uncharacterized	+	582	348	no
YJR053W	YJR054W	Verified	Verified	+	338	338	yes
YDL171C	YDL172C	Verified	Dubious	-	463	335	no
YLR111W	YLR112W	Uncharacterized	Uncharacterized	+	333	333	no
YMR069W	YMR070W	Verified	Verified	+	527	327	yes
YDR268W	YDR270W	Verified	Verified	+	433	323	yes
YHR189W	YHR190W	Verified	Verified	+	320	320	yes
YBR032W	YBR033W	Uncharacterized	Uncharacterized	+	303	303	no
YPL277C	YPL278C	Uncharacterized	Uncharacterized	-	303	303	no
YIL025C	YIL026C	Dubious	Verified	-	298	298	no
YJL088W	YJL089W	Verified	Verified	+	630	289	yes
YJR139C	YJR140C	Verified	Verified	-	498	261	yes
YDR525W-A	YDR527W	Verified	Verified	+	488	240	yes
YMR175W	YMR175W-A	Verified	Uncharacterized	+	240	240	no
YGR134W	YGR135W	Verified	Verified	+	394	224	yes
YDR359C	YDR361C	Verified	Verified	-	561	200	yes
YOL116W	YOL117W	Verified	Verified	+	304	199	yes
YPL164C	YPL165C	Verified	Verified	-	463	190	yes
YLR162W	YLR162W-A	Verified	Uncharacterized	+	189	189	no
YIR017W-A	YIR018W	Dubious	Verified	+	183	183	no
YKL083W	YKL085W	Dubious	Verified	+	567	175	no
YLR154W-B	YLR154W-C	Dubious	Verified	+	165	165	no
YHR070W	YHR071W	Verified	Verified	+	710	163	yes
YHR032W-A	YHR033W	Dubious	Uncharacterized	+	285	157	no
YOR307C	YOR308C	Verified	Verified	-	336	150	yes
YDR441C	YDR443C	Verified	Verified	-	481	145	yes
YIL099W	YIL100W	Verified	Dubious	+	142	142	no
YHR218W	YHR219W	Uncharacterized	Uncharacterized	+	130	130	no

Gene A	Gene B	Class A	Class B	Strand	Overlap (nt)	Overlap into ORF (nt)	Both Verified
YPR095C	YPR096C	Verified	Verified	-	127	127	yes
YLL046C	YLL048C	Verified	Verified	-	108	108	yes
YER088C	YER089C	Verified	Verified	-	190	107	yes
YOR008W-B	YOR009W	Dubious	Verified	+	102	102	no
YLR310C	YLR311C	Verified	Dubious	-	436	95	no
YPR031W	YPR032W	Verified	Verified	+	202	86	yes
YDR246W-A	YDR247W	Uncharacterized	Verified	+	406	75	no
YMR030W-A	YMR033W	Uncharacterized	Verified	+	116	74	no
YNL278W	YNL279W	Verified	Verified	+	303	71	yes
YMR075C-A	YMR076C	Verified	Verified	-	80	69	yes
YJL077C	YJL078C	Verified	Verified	-	68	68	yes
YPR006C	YPR007C	Verified	Verified	-	289	64	yes
YPR145C-A	YPR146C	Uncharacterized	Dubious	-	422	63	no
YNL203C	YNL205C	Dubious	Dubious	-	55	55	no
YDR024W	YDR025W	Dubious	Verified	+	87	46	no
YMR133W	YMR134W	Verified	Verified	+	45	45	yes
YPR049C	YPR050C	Verified	Dubious	-	40	40	no
YIL100C-A	YIL101C	Dubious	Verified	-	35	35	no
YEL076C-A	YEL077C	Uncharacterized	Uncharacterized	-	31	31	no
YJR036C	YJR038C	Verified	Dubious	-	30	30	no
YEL040W	YEL041W	Verified	Verified	+	73	26	yes
YIL071W-A	YIL072W	Dubious	Verified	+	26	26	no
YGL132W	YGL133W	Dubious	Verified	+	24	24	no
YGL006W	YGL006W-A	Verified	Uncharacterized	+	24	24	no
YAL019W	YAL019W-A	Verified	Dubious	+	23	23	no
YLR227C	YNCL0032C	Verified		-	19	19	no
YBL005W	YBL006W-A	Verified	Dubious	+	574	18	no
YOR301W	YOR303W	Verified	Verified	+	469	14	yes
YPL055C	YPL057C	Verified	Verified	-	557	11	yes
YDL122W	YDL123W	Verified	Verified	+	504	11	yes
YER119C	YER119C-A	Verified	Dubious	-	2	2	no

Supplementary Table S5. Sensitivity of called UTR boundaries to the four segmentation thresholds.

Supplementary Table S5. Sensitivity of called UTR boundaries to the four segmentation thresholds. Each of the four thresholds governing breakpoint detection and expressed-region calling was varied across five values spanning well beyond the released setting on each side (coverage floor 5, 10, 20, 40, 80 reads; merge window 10, 20, 40, 80, 160 nt; effect fraction 0.025, 0.05, 0.10, 0.20, 0.35; expressed fraction 0.01, 0.025, 0.05, 0.10, 0.20), giving 625 combinations. Each combination was run on all six real construction-stage conditions (NS_PS, NS_RDT, S10_PS, S10_RDT, NS, S10) and reduced to the same per-gene maximum across conditions used to build the released annotation. For each combination: the number of genes receiving a boundary call (n_genes); the mean 5′ and 3′ UTR length in nt (mean_five, mean_three); the fraction of the 5,770 genes called at the released settings that retain a call (retained); the fraction of those with both 5′ and 3′ boundaries unchanged to the nucleotide (identical); and the same restricted to the 5,768 genes with a non-zero boundary at the released settings (n_nonzero, ident_nonzero), which excludes genes that are zero-length at both settings and would otherwise count as unchanged trivially. The released combination is marked yes in the released column. Run on the real construction-stage coverage (the actual deposited SRA runs for BioProject PRJNA1022817), with the same algorithm verified to reproduce the released final_utr.tsv on the earlier, three-replicate version of this data; this table therefore demonstrates the sensitivity of the released calls themselves to the four thresholds, not a proxy on an independent library. Two further views follow the 625-row grid: the worst-case range across all 624 non-released combinations, and a one-step-at-a-time breakdown by which threshold moved (Table S4b), since a single median move of 0 nt is not sufficient evidence that a threshold never matters -- it can coexist with a real minority of genes moving substantially. Produced by s15e_supp_table_real.R and s15g_sensitivity_extended.py.

coverage_floor	merge_window	effect_frac	expr_frac	released	n_genes	mean_five	mean_three	retained	identical	n_nonzero	ident_nonzero
5	10	0.025	0.010	no	6,182	181.9	160.7	1.0000	0.7820	5,768	0.7819
5	10	0.025	0.025	no	6,182	180.2	160.4	1.0000	0.7823	5,768	0.7822
5	10	0.025	0.050	no	6,182	174.9	160.0	1.0000	0.7367	5,768	0.7367
5	10	0.025	0.100	no	6,182	164.3	159.1	1.0000	0.6546	5,768	0.6545
5	10	0.025	0.200	no	6,182	153.0	157.4	1.0000	0.5844	5,768	0.5843
10	10	0.025	0.010	no	6,024	173.5	158.7	1.0000	0.8236	5,768	0.8235
10	10	0.025	0.025	no	6,024	171.8	158.4	1.0000	0.8241	5,768	0.8240
10	10	0.025	0.050	no	6,024	166.2	158.0	1.0000	0.7776	5,768	0.7776
10	10	0.025	0.100	no	6,024	155.3	157.0	1.0000	0.6922	5,768	0.6921
10	10	0.025	0.200	no	6,024	144.2	155.5	1.0000	0.6146	5,768	0.6144
20	10	0.025	0.010	no	5,801	159.2	156.4	1.0000	0.9501	5,768	0.9501
20	10	0.025	0.025	no	5,801	157.3	156.1	1.0000	0.9499	5,768	0.9499
20	10	0.025	0.050	no	5,801	151.0	155.6	1.0000	0.8998	5,768	0.8998
20	10	0.025	0.100	no	5,801	140.8	154.5	1.0000	0.8026	5,768	0.8025
20	10	0.025	0.200	no	5,801	129.7	153.4	1.0000	0.7078	5,768	0.7077
40	10	0.025	0.010	no	5,402	139.4	151.8	0.9326	0.7393	5,380	0.7392
40	10	0.025	0.025	no	5,402	136.6	151.3	0.9326	0.7382	5,380	0.7381
40	10	0.025	0.050	no	5,402	131.0	150.8	0.9326	0.6941	5,380	0.6941
40	10	0.025	0.100	no	5,402	120.4	150.1	0.9326	0.6092	5,380	0.6091
40	10	0.025	0.200	no	5,402	109.5	148.6	0.9326	0.5363	5,380	0.5362
80	10	0.025	0.010	no	4,539	117.1	144.1	0.7847	0.5733	4,527	0.5732
80	10	0.025	0.025	no	4,539	114.9	143.4	0.7847	0.5755	4,527	0.5754
80	10	0.025	0.050	no	4,539	109.1	143.1	0.7847	0.5409	4,527	0.5408
80	10	0.025	0.100	no	4,539	97.7	142.4	0.7847	0.4680	4,527	0.4679
80	10	0.025	0.200	no	4,539	88.7	141.8	0.7847	0.4163	4,527	0.4162
5	20	0.025	0.010	no	6,181	181.8	160.5	1.0000	0.7837	5,768	0.7836

coverage_floor	merge_window	effect_frac	expr_frac	released	n_genes	mean_five	mean_three	retained	identical	n_nonzero	ident_nonzero
5	20	0.025	0.025	no	6,181	179.9	160.2	1.0000	0.7827	5,768	0.7826
5	20	0.025	0.050	no	6,181	174.3	159.9	1.0000	0.7367	5,768	0.7367
5	20	0.025	0.100	no	6,181	164.0	159.0	1.0000	0.6558	5,768	0.6557
5	20	0.025	0.200	no	6,181	152.9	157.3	1.0000	0.5870	5,768	0.5869
10	20	0.025	0.010	no	6,023	173.5	158.5	1.0000	0.8255	5,768	0.8254
10	20	0.025	0.025	no	6,023	171.6	158.2	1.0000	0.8246	5,768	0.8245
10	20	0.025	0.050	no	6,023	165.7	157.8	1.0000	0.7778	5,768	0.7777
10	20	0.025	0.100	no	6,023	155.1	156.9	1.0000	0.6938	5,768	0.6937
10	20	0.025	0.200	no	6,023	144.2	155.5	1.0000	0.6184	5,768	0.6182
20	20	0.025	0.010	no	5,801	159.1	156.3	1.0000	0.9529	5,768	0.9528
20	20	0.025	0.025	no	5,801	157.0	156.0	1.0000	0.9513	5,768	0.9513
20	20	0.025	0.050	no	5,801	150.4	155.5	1.0000	0.9016	5,768	0.9015
20	20	0.025	0.100	no	5,801	140.4	154.6	1.0000	0.8055	5,768	0.8055
20	20	0.025	0.200	no	5,801	129.6	153.4	1.0000	0.7128	5,768	0.7127
40	20	0.025	0.010	no	5,399	139.5	151.7	0.9322	0.7401	5,378	0.7401
40	20	0.025	0.025	no	5,399	136.6	151.2	0.9322	0.7371	5,378	0.7371
40	20	0.025	0.050	no	5,399	130.8	150.8	0.9322	0.6929	5,378	0.6928
40	20	0.025	0.100	no	5,399	120.3	150.1	0.9322	0.6096	5,378	0.6095
40	20	0.025	0.200	no	5,399	109.6	148.7	0.9322	0.5382	5,378	0.5381
80	20	0.025	0.010	no	4,537	117.2	144.0	0.7844	0.5734	4,525	0.5733
80	20	0.025	0.025	no	4,537	114.8	143.5	0.7844	0.5745	4,525	0.5744
80	20	0.025	0.050	no	4,537	108.8	143.1	0.7844	0.5389	4,525	0.5388
80	20	0.025	0.100	no	4,537	97.8	142.5	0.7844	0.4671	4,525	0.4670
80	20	0.025	0.200	no	4,537	88.6	141.8	0.7844	0.4165	4,525	0.4164
5	40	0.025	0.010	no	6,178	181.7	160.5	1.0000	0.7863	5,768	0.7862
5	40	0.025	0.025	no	6,178	179.8	160.4	1.0000	0.7842	5,768	0.7842
5	40	0.025	0.050	no	6,178	174.0	159.8	1.0000	0.7373	5,768	0.7372
5	40	0.025	0.100	no	6,178	164.0	159.0	1.0000	0.6610	5,768	0.6609
5	40	0.025	0.200	no	6,178	153.0	157.4	1.0000	0.5922	5,768	0.5921
10	40	0.025	0.010	no	6,019	173.4	158.5	1.0000	0.8286	5,768	0.8285
10	40	0.025	0.025	no	6,019	171.5	158.3	1.0000	0.8265	5,768	0.8265
10	40	0.025	0.050	no	6,019	165.4	157.8	1.0000	0.7790	5,768	0.7790
10	40	0.025	0.100	no	6,019	155.1	157.0	1.0000	0.6998	5,768	0.6997
10	40	0.025	0.200	no	6,019	144.2	155.6	1.0000	0.6241	5,768	0.6240
20	40	0.025	0.010	no	5,795	159.0	156.3	1.0000	0.9565	5,768	0.9565
20	40	0.025	0.025	no	5,795	156.7	156.2	1.0000	0.9537	5,768	0.9537
20	40	0.025	0.050	no	5,795	149.9	155.5	1.0000	0.9038	5,768	0.9038
20	40	0.025	0.100	no	5,795	140.3	154.7	1.0000	0.8127	5,768	0.8126
20	40	0.025	0.200	no	5,795	129.5	153.6	1.0000	0.7199	5,768	0.7198

coverage_floor	merge_window	effect_frac	expr_frac	released	n_genes	mean_five	mean_three	retained	identical	n_nonzero	ident_nonzero
40	40	0.025	0.010	no	5,394	139.6	151.7	0.9321	0.7419	5,377	0.7419
40	40	0.025	0.025	no	5,394	136.4	151.5	0.9321	0.7374	5,377	0.7374
40	40	0.025	0.050	no	5,394	130.2	150.8	0.9321	0.6923	5,377	0.6922
40	40	0.025	0.100	no	5,394	120.1	150.2	0.9321	0.6140	5,377	0.6139
40	40	0.025	0.200	no	5,394	109.6	148.7	0.9321	0.5420	5,377	0.5419
80	40	0.025	0.010	no	4,531	117.3	144.1	0.7839	0.5740	4,522	0.5739
80	40	0.025	0.025	no	4,531	114.8	143.8	0.7839	0.5731	4,522	0.5730
80	40	0.025	0.050	no	4,531	108.2	143.2	0.7839	0.5350	4,522	0.5349
80	40	0.025	0.100	no	4,531	97.5	142.7	0.7839	0.4683	4,522	0.4682
80	40	0.025	0.200	no	4,531	88.6	142.0	0.7839	0.4174	4,522	0.4173
5	80	0.025	0.010	no	6,150	181.5	159.8	0.9969	0.7749	5,750	0.7748
5	80	0.025	0.025	no	6,150	179.4	159.7	0.9969	0.7695	5,750	0.7694
5	80	0.025	0.050	no	6,150	172.8	159.3	0.9969	0.7234	5,750	0.7233
5	80	0.025	0.100	no	6,150	163.7	158.3	0.9969	0.6511	5,750	0.6510
5	80	0.025	0.200	no	6,150	153.0	156.7	0.9969	0.5817	5,750	0.5816
10	80	0.025	0.010	no	5,988	173.2	157.9	0.9965	0.8158	5,748	0.8158
10	80	0.025	0.025	no	5,988	171.0	157.7	0.9965	0.8103	5,748	0.8102
10	80	0.025	0.050	no	5,988	164.2	157.3	0.9965	0.7638	5,748	0.7637
10	80	0.025	0.100	no	5,988	154.8	156.4	0.9965	0.6889	5,748	0.6888
10	80	0.025	0.200	no	5,988	144.0	155.0	0.9965	0.6125	5,748	0.6124
20	80	0.025	0.010	no	5,762	159.1	155.6	0.9948	0.9423	5,738	0.9423
20	80	0.025	0.025	no	5,762	156.6	155.5	0.9948	0.9359	5,738	0.9359
20	80	0.025	0.050	no	5,762	149.0	155.0	0.9948	0.8875	5,738	0.8874
20	80	0.025	0.100	no	5,762	140.3	153.9	0.9948	0.8010	5,738	0.8010
20	80	0.025	0.200	no	5,762	129.7	152.8	0.9948	0.7080	5,738	0.7079
40	80	0.025	0.010	no	5,357	139.7	150.9	0.9262	0.7335	5,343	0.7335
40	80	0.025	0.025	no	5,357	136.2	150.8	0.9262	0.7253	5,343	0.7252
40	80	0.025	0.050	no	5,357	129.2	150.2	0.9262	0.6798	5,343	0.6798
40	80	0.025	0.100	no	5,357	119.8	149.4	0.9262	0.6068	5,343	0.6068
40	80	0.025	0.200	no	5,357	109.8	147.9	0.9262	0.5350	5,343	0.5349
80	80	0.025	0.010	no	4,495	117.5	143.7	0.7782	0.5693	4,489	0.5692
80	80	0.025	0.025	no	4,495	114.5	143.4	0.7782	0.5641	4,489	0.5640
80	80	0.025	0.050	no	4,495	106.7	142.7	0.7782	0.5245	4,489	0.5244
80	80	0.025	0.100	no	4,495	97.1	142.2	0.7782	0.4635	4,489	0.4634
80	80	0.025	0.200	no	4,495	88.4	141.6	0.7782	0.4122	4,489	0.4121
5	160	0.025	0.010	no	6,029	180.1	158.0	0.9799	0.7501	5,652	0.7500
5	160	0.025	0.025	no	6,029	178.0	157.9	0.9799	0.7412	5,652	0.7412
5	160	0.025	0.050	no	6,029	170.9	157.5	0.9799	0.6984	5,652	0.6983
5	160	0.025	0.100	no	6,029	162.2	156.6	0.9799	0.6279	5,652	0.6277

coverage_floor	merge_window	effect_frac	expr_frac	released	n_genes	mean_five	mean_three	retained	identical	n_nonzero	ident_nonzero
5	160	0.025	0.200	no	6,029	151.8	155.0	0.9799	0.5617	5,652	0.5616
10	160	0.025	0.010	no	5,859	171.8	156.2	0.9771	0.7909	5,636	0.7908
10	160	0.025	0.025	no	5,859	169.5	156.1	0.9771	0.7817	5,636	0.7816
10	160	0.025	0.050	no	5,859	162.3	155.7	0.9771	0.7386	5,636	0.7385
10	160	0.025	0.100	no	5,859	153.2	154.8	0.9771	0.6653	5,636	0.6652
10	160	0.025	0.200	no	5,859	142.7	153.3	0.9771	0.5922	5,636	0.5921
20	160	0.025	0.010	no	5,619	157.4	154.2	0.9712	0.9149	5,602	0.9149
20	160	0.025	0.025	no	5,619	154.8	154.1	0.9712	0.9051	5,602	0.9050
20	160	0.025	0.050	no	5,619	146.7	153.7	0.9712	0.8599	5,602	0.8599
20	160	0.025	0.100	no	5,619	137.9	152.8	0.9712	0.7748	5,602	0.7747
20	160	0.025	0.200	no	5,619	128.2	151.5	0.9712	0.6856	5,602	0.6855
40	160	0.025	0.010	no	5,206	138.4	149.5	0.9007	0.7170	5,196	0.7169
40	160	0.025	0.025	no	5,206	134.8	149.4	0.9007	0.7052	5,196	0.7052
40	160	0.025	0.050	no	5,206	127.0	148.9	0.9007	0.6606	5,196	0.6605
40	160	0.025	0.100	no	5,206	117.8	148.2	0.9007	0.5890	5,196	0.5889
40	160	0.025	0.200	no	5,206	108.1	146.5	0.9007	0.5215	5,196	0.5214
80	160	0.025	0.010	no	4,370	116.5	141.9	0.7567	0.5586	4,365	0.5585
80	160	0.025	0.025	no	4,370	113.1	141.8	0.7567	0.5490	4,365	0.5489
80	160	0.025	0.050	no	4,370	104.1	141.1	0.7567	0.5080	4,365	0.5079
80	160	0.025	0.100	no	4,370	95.2	140.4	0.7567	0.4505	4,365	0.4504
80	160	0.025	0.200	no	4,370	86.9	139.8	0.7567	0.4031	4,365	0.4030
5	10	0.050	0.010	no	6,181	181.9	160.6	1.0000	0.7823	5,768	0.7822
5	10	0.050	0.025	no	6,181	181.2	160.3	1.0000	0.7899	5,768	0.7899
5	10	0.050	0.050	no	6,181	178.8	160.0	1.0000	0.7858	5,768	0.7857
5	10	0.050	0.100	no	6,181	171.5	159.0	1.0000	0.7184	5,768	0.7183
5	10	0.050	0.200	no	6,181	157.5	157.3	1.0000	0.6114	5,768	0.6113
10	10	0.050	0.010	no	6,023	173.5	158.5	1.0000	0.8241	5,768	0.8240
10	10	0.050	0.025	no	6,023	172.8	158.2	1.0000	0.8317	5,768	0.8317
10	10	0.050	0.050	no	6,023	170.3	157.9	1.0000	0.8274	5,768	0.8273
10	10	0.050	0.100	no	6,023	162.6	156.9	1.0000	0.7570	5,768	0.7569
10	10	0.050	0.200	no	6,023	148.9	155.4	1.0000	0.6423	5,768	0.6422
20	10	0.050	0.010	no	5,796	159.2	156.3	0.9998	0.9506	5,767	0.9506
20	10	0.050	0.025	no	5,796	158.4	156.0	0.9998	0.9582	5,767	0.9582
20	10	0.050	0.050	no	5,796	155.6	155.5	0.9998	0.9539	5,767	0.9539
20	10	0.050	0.100	no	5,796	148.6	154.4	0.9998	0.8710	5,767	0.8710
20	10	0.050	0.200	no	5,796	134.8	153.2	0.9998	0.7372	5,767	0.7371
40	10	0.050	0.010	no	5,398	139.4	151.7	0.9324	0.7396	5,379	0.7395
40	10	0.050	0.025	no	5,398	138.3	151.2	0.9324	0.7476	5,379	0.7475
40	10	0.050	0.050	no	5,398	136.0	150.7	0.9324	0.7439	5,379	0.7438

coverage_floor	merge_window	effect_frac	expr_frac	released	n_genes	mean_five	mean_three	retained	identical	n_nonzero	ident_nonzero
40	10	0.050	0.100	no	5,398	129.0	150.1	0.9324	0.6740	5,379	0.6739
40	10	0.050	0.200	no	5,398	115.3	148.5	0.9324	0.5665	5,379	0.5665
80	10	0.050	0.010	no	4,539	117.0	144.1	0.7847	0.5733	4,527	0.5732
80	10	0.050	0.025	no	4,539	115.8	143.5	0.7847	0.5819	4,527	0.5818
80	10	0.050	0.050	no	4,539	113.7	143.0	0.7847	0.5788	4,527	0.5787
80	10	0.050	0.100	no	4,539	106.8	142.4	0.7847	0.5254	4,527	0.5253
80	10	0.050	0.200	no	4,539	94.8	141.8	0.7847	0.4472	4,527	0.4471
5	20	0.050	0.010	no	6,179	181.8	160.4	1.0000	0.7844	5,768	0.7843
5	20	0.050	0.025	no	6,179	181.0	160.1	1.0000	0.7906	5,768	0.7906
5	20	0.050	0.050	no	6,179	178.5	159.8	1.0000	0.7868	5,768	0.7868
5	20	0.050	0.100	no	6,179	170.6	158.9	1.0000	0.7125	5,768	0.7124
5	20	0.050	0.200	no	6,179	156.9	157.2	1.0000	0.6102	5,768	0.6101
10	20	0.050	0.010	no	6,021	173.5	158.3	1.0000	0.8263	5,768	0.8263
10	20	0.050	0.025	no	6,021	172.7	158.1	1.0000	0.8326	5,768	0.8325
10	20	0.050	0.050	no	6,021	170.1	157.8	1.0000	0.8288	5,768	0.8287
10	20	0.050	0.100	no	6,021	161.9	156.8	1.0000	0.7513	5,768	0.7512
10	20	0.050	0.200	no	6,021	148.4	155.4	1.0000	0.6425	5,768	0.6423
20	20	0.050	0.010	no	5,795	159.1	156.1	0.9998	0.9537	5,767	0.9537
20	20	0.050	0.025	no	5,795	158.2	155.8	0.9998	0.9598	5,767	0.9598
20	20	0.050	0.050	no	5,795	155.2	155.4	0.9998	0.9560	5,767	0.9560
20	20	0.050	0.100	no	5,795	147.7	154.5	0.9998	0.8662	5,767	0.8661
20	20	0.050	0.200	no	5,795	134.1	153.2	0.9998	0.7383	5,767	0.7382
40	20	0.050	0.010	no	5,395	139.6	151.6	0.9321	0.7408	5,377	0.7407
40	20	0.050	0.025	no	5,395	138.4	151.1	0.9321	0.7473	5,377	0.7473
40	20	0.050	0.050	no	5,395	135.9	150.8	0.9321	0.7436	5,377	0.7435
40	20	0.050	0.100	no	5,395	128.4	150.2	0.9321	0.6675	5,377	0.6675
40	20	0.050	0.200	no	5,395	114.7	148.5	0.9321	0.5642	5,377	0.5641
80	20	0.050	0.010	no	4,536	117.2	144.0	0.7844	0.5738	4,525	0.5737
80	20	0.050	0.025	no	4,536	115.9	143.4	0.7844	0.5817	4,525	0.5817
80	20	0.050	0.050	no	4,536	113.6	143.1	0.7844	0.5793	4,525	0.5792
80	20	0.050	0.100	no	4,536	106.2	142.5	0.7844	0.5203	4,525	0.5202
80	20	0.050	0.200	no	4,536	94.0	141.8	0.7844	0.4428	4,525	0.4427
5	40	0.050	0.010	no	6,176	181.7	160.5	1.0000	0.7870	5,768	0.7869
5	40	0.050	0.025	no	6,176	181.0	160.4	1.0000	0.7924	5,768	0.7923
5	40	0.050	0.050	no	6,176	178.3	159.9	1.0000	0.7882	5,768	0.7881
5	40	0.050	0.100	no	6,176	169.9	159.1	1.0000	0.7073	5,768	0.7072
5	40	0.050	0.200	no	6,176	156.0	157.4	1.0000	0.6109	5,768	0.6108
10	40	0.050	0.010	no	6,017	173.4	158.5	1.0000	0.8296	5,768	0.8296
10	40	0.050	0.025	no	6,017	172.7	158.4	1.0000	0.8350	5,768	0.8350

coverage_floor	merge_window	effect_frac	expr_frac	released	n_genes	mean_five	mean_three	retained	identical	n_nonzero	ident_nonzero
10	40	0.050	0.050	no	6,017	169.8	157.9	1.0000	0.8307	5,768	0.8306
10	40	0.050	0.100	no	6,017	161.2	157.1	1.0000	0.7471	5,768	0.7471
10	40	0.050	0.200	no	6,017	147.4	155.6	1.0000	0.6437	5,768	0.6436
20	40	0.050	0.010	no	5,793	159.0	156.3	1.0000	0.9575	5,768	0.9575
20	40	0.050	0.025	no	5,793	158.2	156.2	1.0000	0.9627	5,768	0.9627
20	40	0.050	0.050	no	5,793	154.9	155.6	1.0000	0.9586	5,768	0.9586
20	40	0.050	0.100	no	5,793	146.9	154.7	1.0000	0.8627	5,768	0.8627
20	40	0.050	0.200	no	5,793	132.9	153.6	1.0000	0.7404	5,768	0.7403
40	40	0.050	0.010	no	5,392	139.6	151.7	0.9319	0.7426	5,376	0.7426
40	40	0.050	0.025	no	5,392	138.5	151.4	0.9319	0.7486	5,376	0.7485
40	40	0.050	0.050	no	5,392	135.5	150.8	0.9319	0.7439	5,376	0.7439
40	40	0.050	0.100	no	5,392	127.6	150.3	0.9319	0.6623	5,376	0.6622
40	40	0.050	0.200	no	5,392	113.3	148.7	0.9319	0.5628	5,376	0.5627
80	40	0.050	0.010	no	4,530	117.3	144.1	0.7839	0.5742	4,522	0.5741
80	40	0.050	0.025	no	4,530	116.0	143.8	0.7839	0.5817	4,522	0.5816
80	40	0.050	0.050	no	4,530	113.1	143.1	0.7839	0.5782	4,522	0.5781
80	40	0.050	0.100	no	4,530	105.2	142.6	0.7839	0.5136	4,522	0.5135
80	40	0.050	0.200	no	4,530	92.5	142.0	0.7839	0.4393	4,522	0.4392
5	80	0.050	0.010	no	6,147	181.5	159.9	0.9965	0.7758	5,748	0.7757
5	80	0.050	0.025	no	6,147	180.7	159.8	0.9965	0.7807	5,748	0.7806
5	80	0.050	0.050	no	6,147	177.5	159.4	0.9965	0.7708	5,748	0.7707
5	80	0.050	0.100	no	6,147	168.1	158.4	0.9965	0.6826	5,748	0.6825
5	80	0.050	0.200	no	6,147	154.7	156.8	0.9965	0.5943	5,748	0.5941
10	80	0.050	0.010	no	5,985	173.2	157.9	0.9962	0.8172	5,746	0.8171
10	80	0.050	0.025	no	5,985	172.4	157.8	0.9962	0.8220	5,746	0.8220
10	80	0.050	0.050	no	5,985	169.0	157.4	0.9962	0.8118	5,746	0.8117
10	80	0.050	0.100	no	5,985	159.3	156.5	0.9962	0.7211	5,746	0.7210
10	80	0.050	0.200	no	5,985	145.9	155.0	0.9962	0.6256	5,746	0.6255
20	80	0.050	0.010	no	5,758	159.1	155.7	0.9943	0.9437	5,735	0.9437
20	80	0.050	0.025	no	5,758	158.2	155.6	0.9943	0.9486	5,735	0.9486
20	80	0.050	0.050	no	5,758	154.4	155.1	0.9943	0.9379	5,735	0.9379
20	80	0.050	0.100	no	5,758	145.3	154.0	0.9943	0.8356	5,735	0.8356
20	80	0.050	0.200	no	5,758	131.6	152.9	0.9943	0.7216	5,735	0.7215
40	80	0.050	0.010	no	5,355	139.7	150.9	0.9260	0.7344	5,342	0.7344
40	80	0.050	0.025	no	5,355	138.5	150.7	0.9260	0.7400	5,342	0.7400
40	80	0.050	0.050	no	5,355	134.9	150.2	0.9260	0.7288	5,342	0.7288
40	80	0.050	0.100	no	5,355	125.5	149.5	0.9260	0.6388	5,342	0.6387
40	80	0.050	0.200	no	5,355	111.8	147.8	0.9260	0.5486	5,342	0.5485
80	80	0.050	0.010	no	4,495	117.5	143.7	0.7782	0.5699	4,489	0.5698

coverage_floor	merge_window	effect_frac	expr_frac	released	n_genes	mean_five	mean_three	retained	identical	n_nonzero	ident_nonzero
80	80	0.050	0.025	no	4,495	116.1	143.4	0.7782	0.5771	4,489	0.5770
80	80	0.050	0.050	no	4,495	112.6	142.8	0.7782	0.5668	4,489	0.5667
80	80	0.050	0.100	no	4,495	103.1	142.2	0.7782	0.4949	4,489	0.4948
80	80	0.050	0.200	no	4,495	90.7	141.6	0.7782	0.4274	4,489	0.4273
5	160	0.050	0.010	no	6,027	180.2	158.0	0.9795	0.7504	5,650	0.7503
5	160	0.050	0.025	no	6,027	179.4	157.9	0.9795	0.7544	5,650	0.7543
5	160	0.050	0.050	no	6,027	174.9	157.5	0.9795	0.7344	5,650	0.7343
5	160	0.050	0.100	no	6,027	164.2	156.6	0.9795	0.6401	5,650	0.6400
5	160	0.050	0.200	no	6,027	152.3	155.0	0.9795	0.5665	5,650	0.5664
10	160	0.050	0.010	no	5,857	171.8	156.2	0.9768	0.7912	5,634	0.7911
10	160	0.050	0.025	no	5,857	171.0	156.1	0.9768	0.7952	5,634	0.7952
10	160	0.050	0.050	no	5,857	166.3	155.6	0.9768	0.7748	5,634	0.7748
10	160	0.050	0.100	no	5,857	155.3	154.8	0.9768	0.6778	5,634	0.6777
10	160	0.050	0.200	no	5,857	143.3	153.3	0.9768	0.5971	5,634	0.5969
20	160	0.050	0.010	no	5,614	157.6	154.2	0.9704	0.9152	5,597	0.9151
20	160	0.050	0.025	no	5,614	156.7	154.1	0.9704	0.9193	5,597	0.9192
20	160	0.050	0.050	no	5,614	151.3	153.6	0.9704	0.8978	5,597	0.8978
20	160	0.050	0.100	no	5,614	140.3	152.7	0.9704	0.7891	5,597	0.7890
20	160	0.050	0.200	no	5,614	128.9	151.4	0.9704	0.6907	5,597	0.6905
40	160	0.050	0.010	no	5,205	138.5	149.5	0.9005	0.7173	5,195	0.7172
40	160	0.050	0.025	no	5,205	137.3	149.3	0.9005	0.7215	5,195	0.7215
40	160	0.050	0.050	no	5,205	131.9	148.8	0.9005	0.6984	5,195	0.6984
40	160	0.050	0.100	no	5,205	120.7	148.1	0.9005	0.6028	5,195	0.6027
40	160	0.050	0.200	no	5,205	108.9	146.5	0.9005	0.5269	5,195	0.5269
80	160	0.050	0.010	no	4,369	116.5	141.9	0.7565	0.5590	4,364	0.5589
80	160	0.050	0.025	no	4,369	115.1	141.6	0.7565	0.5652	4,364	0.5651
80	160	0.050	0.050	no	4,369	109.5	141.0	0.7565	0.5430	4,364	0.5429
80	160	0.050	0.100	no	4,369	98.2	140.4	0.7565	0.4658	4,364	0.4656
80	160	0.050	0.200	no	4,369	87.9	139.8	0.7565	0.4096	4,364	0.4095
5	10	0.100	0.010	no	6,164	181.0	158.8	0.9988	0.7770	5,761	0.7769
5	10	0.100	0.025	no	6,164	180.5	158.6	0.9988	0.7852	5,761	0.7851
5	10	0.100	0.050	no	6,164	179.2	158.1	0.9988	0.7932	5,761	0.7931
5	10	0.100	0.100	no	6,164	176.5	157.2	0.9988	0.7682	5,761	0.7681
5	10	0.100	0.200	no	6,164	166.3	155.5	0.9988	0.7009	5,761	0.7007
10	10	0.100	0.010	no	5,999	172.5	156.8	0.9984	0.8188	5,759	0.8187
10	10	0.100	0.025	no	5,999	172.0	156.6	0.9984	0.8269	5,759	0.8269
10	10	0.100	0.050	no	5,999	170.7	156.1	0.9984	0.8353	5,759	0.8352
10	10	0.100	0.100	no	5,999	167.9	155.2	0.9984	0.8089	5,759	0.8088
10	10	0.100	0.200	no	5,999	158.0	153.6	0.9984	0.7346	5,759	0.7345

coverage_floor	merge_window	effect_frac	expr_frac	released	n_genes	mean_five	mean_three	retained	identical	n_nonzero	ident_nonzero
20	10	0.100	0.010	no	5,770	157.8	154.4	0.9979	0.9446	5,756	0.9446
20	10	0.100	0.025	no	5,770	157.3	154.2	0.9979	0.9529	5,756	0.9529
20	10	0.100	0.050	no	5,770	155.7	153.6	0.9979	0.9625	5,756	0.9625
20	10	0.100	0.100	no	5,770	153.1	152.7	0.9979	0.9300	5,756	0.9300
20	10	0.100	0.200	no	5,770	143.8	151.3	0.9979	0.8381	5,756	0.8381
40	10	0.100	0.010	no	5,367	138.1	149.1	0.9286	0.7335	5,357	0.7334
40	10	0.100	0.025	no	5,367	137.3	148.8	0.9286	0.7426	5,357	0.7426
40	10	0.100	0.050	no	5,367	136.0	148.2	0.9286	0.7482	5,357	0.7482
40	10	0.100	0.100	no	5,367	133.3	147.5	0.9286	0.7242	5,357	0.7241
40	10	0.100	0.200	no	5,367	124.1	146.0	0.9286	0.6534	5,357	0.6534
80	10	0.100	0.010	no	4,501	116.0	142.4	0.7790	0.5689	4,494	0.5688
80	10	0.100	0.025	no	4,501	115.2	142.0	0.7790	0.5791	4,494	0.5790
80	10	0.100	0.050	no	4,501	113.7	141.4	0.7790	0.5829	4,494	0.5828
80	10	0.100	0.100	no	4,501	111.4	140.9	0.7790	0.5657	4,494	0.5656
80	10	0.100	0.200	no	4,501	103.5	139.9	0.7790	0.5152	4,494	0.5151
5	20	0.100	0.010	no	6,165	180.7	158.9	0.9993	0.7843	5,764	0.7842
5	20	0.100	0.025	no	6,165	180.3	158.7	0.9993	0.7924	5,764	0.7923
5	20	0.100	0.050	no	6,165	179.1	158.3	0.9993	0.8033	5,764	0.8033
5	20	0.100	0.100	no	6,165	175.8	157.3	0.9993	0.7737	5,764	0.7736
5	20	0.100	0.200	no	6,165	165.3	155.7	0.9993	0.7003	5,764	0.7002
10	20	0.100	0.010	no	5,998	172.4	156.9	0.9990	0.8265	5,762	0.8264
10	20	0.100	0.025	no	5,998	172.0	156.7	0.9990	0.8347	5,762	0.8346
10	20	0.100	0.050	no	5,998	170.7	156.3	0.9990	0.8459	5,762	0.8459
10	20	0.100	0.100	no	5,998	167.4	155.4	0.9990	0.8147	5,762	0.8146
10	20	0.100	0.200	no	5,998	157.2	153.9	0.9990	0.7351	5,762	0.7350
20	20	0.100	0.010	no	5,769	157.8	154.6	0.9983	0.9552	5,758	0.9552
20	20	0.100	0.025	no	5,769	157.3	154.4	0.9983	0.9635	5,758	0.9635
20	20	0.100	0.050	no	5,769	155.8	153.9	0.9983	0.9769	5,758	0.9769
20	20	0.100	0.100	no	5,769	152.7	152.9	0.9983	0.9385	5,758	0.9385
20	20	0.100	0.200	no	5,769	143.0	151.6	0.9983	0.8392	5,758	0.8392
40	20	0.100	0.010	no	5,368	138.2	150.0	0.9295	0.7406	5,362	0.7406
40	20	0.100	0.025	no	5,368	137.5	149.6	0.9295	0.7500	5,362	0.7499
40	20	0.100	0.050	no	5,368	136.3	149.1	0.9295	0.7593	5,362	0.7592
40	20	0.100	0.100	no	5,368	133.1	148.3	0.9295	0.7291	5,362	0.7290
40	20	0.100	0.200	no	5,368	123.4	146.7	0.9295	0.6528	5,362	0.6527
80	20	0.100	0.010	no	4,507	116.2	142.5	0.7806	0.5739	4,503	0.5738
80	20	0.100	0.025	no	4,507	115.3	141.9	0.7806	0.5846	4,503	0.5845
80	20	0.100	0.050	no	4,507	114.1	141.5	0.7806	0.5908	4,503	0.5907
80	20	0.100	0.100	no	4,507	111.3	140.8	0.7806	0.5684	4,503	0.5683

coverage_floor	merge_window	effect_frac	expr_frac	released	n_genes	mean_five	mean_three	retained	identical	n_nonzero	ident_nonzero
80	20	0.100	0.200	no	4,507	102.9	139.9	0.7806	0.5133	4,503	0.5132
5	40	0.100	0.010	no	6,164	180.9	159.3	1.0000	0.8023	5,768	0.8022
5	40	0.100	0.025	no	6,164	180.5	159.2	1.0000	0.8101	5,768	0.8100
5	40	0.100	0.050	no	6,164	179.1	158.7	1.0000	0.8239	5,768	0.8239
5	40	0.100	0.100	no	6,164	175.3	158.0	1.0000	0.7877	5,768	0.7876
5	40	0.100	0.200	no	6,164	164.1	156.2	1.0000	0.7026	5,768	0.7025
10	40	0.100	0.010	no	6,000	172.6	157.2	1.0000	0.8456	5,768	0.8455
10	40	0.100	0.025	no	6,000	172.2	157.1	1.0000	0.8534	5,768	0.8533
10	40	0.100	0.050	no	6,000	170.7	156.7	1.0000	0.8676	5,768	0.8675
10	40	0.100	0.100	no	6,000	166.9	155.9	1.0000	0.8298	5,768	0.8298
10	40	0.100	0.200	no	6,000	155.8	154.5	1.0000	0.7380	5,768	0.7379
20	40	0.100	0.010	no	5,770	158.2	155.0	1.0000	0.9757	5,768	0.9757
20	40	0.100	0.025	no	5,770	157.7	155.0	1.0000	0.9837	5,768	0.9837
20	40	0.100	0.050	yes	5,770	156.0	154.4	1.0000	1.0000	5,768	1.0000
20	40	0.100	0.100	no	5,770	152.3	153.5	1.0000	0.9549	5,768	0.9549
20	40	0.100	0.200	no	5,770	141.6	152.2	1.0000	0.8426	5,768	0.8426
40	40	0.100	0.010	no	5,368	138.6	150.5	0.9303	0.7565	5,367	0.7565
40	40	0.100	0.025	no	5,368	137.8	150.3	0.9303	0.7656	5,367	0.7656
40	40	0.100	0.050	no	5,368	136.3	149.7	0.9303	0.7766	5,367	0.7766
40	40	0.100	0.100	no	5,368	132.5	149.1	0.9303	0.7416	5,367	0.7416
40	40	0.100	0.200	no	5,368	121.9	147.5	0.9303	0.6526	5,367	0.6525
80	40	0.100	0.010	no	4,506	116.6	143.0	0.7809	0.5841	4,505	0.5840
80	40	0.100	0.025	no	4,506	115.8	142.7	0.7809	0.5945	4,505	0.5945
80	40	0.100	0.050	no	4,506	114.2	142.0	0.7809	0.6021	4,505	0.6020
80	40	0.100	0.100	no	4,506	110.8	141.5	0.7809	0.5755	4,505	0.5754
80	40	0.100	0.200	no	4,506	101.3	140.5	0.7809	0.5124	4,505	0.5123
5	80	0.100	0.010	no	6,134	180.9	158.9	0.9957	0.7802	5,743	0.7801
5	80	0.100	0.025	no	6,134	180.5	158.8	0.9957	0.7878	5,743	0.7877
5	80	0.100	0.050	no	6,134	179.1	158.3	0.9957	0.8000	5,743	0.7999
5	80	0.100	0.100	no	6,134	174.2	157.4	0.9957	0.7563	5,743	0.7562
5	80	0.100	0.200	no	6,134	161.7	155.8	0.9957	0.6564	5,743	0.6563
10	80	0.100	0.010	no	5,965	172.5	156.9	0.9951	0.8213	5,740	0.8213
10	80	0.100	0.025	no	5,965	172.1	156.8	0.9951	0.8290	5,740	0.8289
10	80	0.100	0.050	no	5,965	170.6	156.3	0.9951	0.8413	5,740	0.8413
10	80	0.100	0.100	no	5,965	165.6	155.4	0.9951	0.7959	5,740	0.7958
10	80	0.100	0.200	no	5,965	153.1	154.0	0.9951	0.6891	5,740	0.6890
20	80	0.100	0.010	no	5,732	158.2	154.7	0.9929	0.9480	5,727	0.9480
20	80	0.100	0.025	no	5,732	157.8	154.6	0.9929	0.9558	5,727	0.9558
20	80	0.100	0.050	no	5,732	156.1	154.1	0.9929	0.9698	5,727	0.9698

coverage_floor	merge_window	effect_frac	expr_frac	released	n_genes	mean_five	mean_three	retained	identical	n_nonzero	ident_nonzero
20	80	0.100	0.100	no	5,732	151.2	153.1	0.9929	0.9162	5,727	0.9162
20	80	0.100	0.200	no	5,732	139.0	152.0	0.9929	0.7900	5,727	0.7899
40	80	0.100	0.010	no	5,332	138.9	150.3	0.9239	0.7385	5,330	0.7385
40	80	0.100	0.025	no	5,332	138.2	150.1	0.9239	0.7475	5,330	0.7475
40	80	0.100	0.050	no	5,332	136.5	149.6	0.9239	0.7567	5,330	0.7567
40	80	0.100	0.100	no	5,332	131.6	148.8	0.9239	0.7124	5,330	0.7124
40	80	0.100	0.200	no	5,332	119.4	147.2	0.9239	0.6113	5,330	0.6113
80	80	0.100	0.010	no	4,483	116.8	142.7	0.7768	0.5723	4,481	0.5722
80	80	0.100	0.025	no	4,483	116.0	142.4	0.7768	0.5826	4,481	0.5825
80	80	0.100	0.050	no	4,483	114.3	141.8	0.7768	0.5888	4,481	0.5887
80	80	0.100	0.100	no	4,483	109.5	141.2	0.7768	0.5542	4,481	0.5541
80	80	0.100	0.200	no	4,483	98.5	140.2	0.7768	0.4788	4,481	0.4787
5	160	0.100	0.010	no	6,011	179.7	157.4	0.9780	0.7516	5,641	0.7515
5	160	0.100	0.025	no	6,011	179.2	157.3	0.9780	0.7592	5,641	0.7591
5	160	0.100	0.050	no	6,011	177.2	156.9	0.9780	0.7679	5,641	0.7678
5	160	0.100	0.100	no	6,011	170.8	156.1	0.9780	0.7048	5,641	0.7047
5	160	0.100	0.200	no	6,011	156.2	154.4	0.9780	0.5965	5,641	0.5963
10	160	0.100	0.010	no	5,834	171.2	155.7	0.9747	0.7929	5,622	0.7928
10	160	0.100	0.025	no	5,834	170.8	155.6	0.9747	0.8005	5,622	0.8004
10	160	0.100	0.050	no	5,834	168.7	155.2	0.9747	0.8094	5,622	0.8093
10	160	0.100	0.100	no	5,834	162.0	154.4	0.9747	0.7440	5,622	0.7439
10	160	0.100	0.200	no	5,834	147.4	152.9	0.9747	0.6286	5,622	0.6284
20	160	0.100	0.010	no	5,595	156.9	153.8	0.9679	0.9164	5,583	0.9164
20	160	0.100	0.025	no	5,595	156.4	153.7	0.9679	0.9243	5,583	0.9242
20	160	0.100	0.050	no	5,595	154.0	153.3	0.9679	0.9341	5,583	0.9341
20	160	0.100	0.100	no	5,595	147.2	152.4	0.9679	0.8593	5,583	0.8592
20	160	0.100	0.200	no	5,595	133.1	151.1	0.9679	0.7252	5,583	0.7251
40	160	0.100	0.010	no	5,187	138.0	149.1	0.8981	0.7190	5,181	0.7190
40	160	0.100	0.025	no	5,187	137.3	148.9	0.8981	0.7279	5,181	0.7279
40	160	0.100	0.050	no	5,187	135.0	148.5	0.8981	0.7337	5,181	0.7336
40	160	0.100	0.100	no	5,187	128.1	147.7	0.8981	0.6694	5,181	0.6694
40	160	0.100	0.200	no	5,187	113.8	145.9	0.8981	0.5591	5,181	0.5590
80	160	0.100	0.010	no	4,362	116.6	141.7	0.7555	0.5604	4,358	0.5603
80	160	0.100	0.025	no	4,362	115.7	141.4	0.7555	0.5710	4,358	0.5709
80	160	0.100	0.050	no	4,362	113.3	140.8	0.7555	0.5738	4,358	0.5737
80	160	0.100	0.100	no	4,362	106.4	140.3	0.7555	0.5221	4,358	0.5220
80	160	0.100	0.200	no	4,362	93.5	139.4	0.7555	0.4400	4,358	0.4399
5	10	0.200	0.010	no	4,453	165.8	146.0	0.7123	0.3564	4,109	0.3563
5	10	0.200	0.025	no	4,453	165.3	145.8	0.7123	0.3669	4,109	0.3668

coverage_floor	merge_window	effect_frac	expr_frac	released	n_genes	mean_five	mean_three	retained	identical	n_nonzero	ident_nonzero
5	10	0.200	0.050	no	4,453	163.6	145.2	0.7123	0.3788	4,109	0.3787
5	10	0.200	0.100	no	4,453	161.7	144.2	0.7123	0.3591	4,109	0.3590
5	10	0.200	0.200	no	4,453	152.5	141.8	0.7123	0.3158	4,109	0.3156
10	10	0.200	0.010	no	4,134	155.2	145.1	0.6842	0.3898	3,947	0.3897
10	10	0.200	0.025	no	4,134	154.6	144.9	0.6842	0.4007	3,947	0.4006
10	10	0.200	0.050	no	4,134	152.8	144.3	0.6842	0.4136	3,947	0.4135
10	10	0.200	0.100	no	4,134	150.6	143.3	0.6842	0.3918	3,947	0.3917
10	10	0.200	0.200	no	4,134	141.1	141.0	0.6842	0.3427	3,947	0.3425
20	10	0.200	0.010	no	3,604	141.4	145.9	0.6239	0.4844	3,599	0.4843
20	10	0.200	0.025	no	3,604	140.7	145.7	0.6239	0.4967	3,599	0.4965
20	10	0.200	0.050	no	3,604	138.3	144.8	0.6239	0.5119	3,599	0.5118
20	10	0.200	0.100	no	3,604	135.8	143.6	0.6239	0.4828	3,599	0.4826
20	10	0.200	0.200	no	3,604	125.4	141.2	0.6239	0.4219	3,599	0.4218
40	10	0.200	0.010	no	2,932	130.4	145.3	0.5076	0.4001	2,928	0.3999
40	10	0.200	0.025	no	2,932	129.1	144.8	0.5076	0.4155	2,928	0.4153
40	10	0.200	0.050	no	2,932	126.6	143.8	0.5076	0.4268	2,928	0.4266
40	10	0.200	0.100	no	2,932	124.0	142.5	0.5076	0.4046	2,928	0.4044
40	10	0.200	0.200	no	2,932	112.3	139.9	0.5076	0.3462	2,928	0.3460
80	10	0.200	0.010	no	2,111	114.9	143.0	0.3655	0.3604	2,108	0.3601
80	10	0.200	0.025	no	2,111	113.2	142.2	0.3655	0.3812	2,108	0.3809
80	10	0.200	0.050	no	2,111	110.1	140.9	0.3655	0.3917	2,108	0.3914
80	10	0.200	0.100	no	2,111	107.6	139.8	0.3655	0.3736	2,108	0.3733
80	10	0.200	0.200	no	2,111	95.3	137.8	0.3655	0.3215	2,108	0.3212
5	20	0.200	0.010	no	4,757	164.7	146.0	0.7645	0.3732	4,410	0.3730
5	20	0.200	0.025	no	4,757	164.2	145.9	0.7645	0.3825	4,410	0.3823
5	20	0.200	0.050	no	4,757	162.7	145.4	0.7645	0.3958	4,410	0.3957
5	20	0.200	0.100	no	4,757	160.6	144.2	0.7645	0.3745	4,410	0.3744
5	20	0.200	0.200	no	4,757	151.1	142.1	0.7645	0.3278	4,410	0.3277
10	20	0.200	0.010	no	4,462	154.2	144.9	0.7409	0.4047	4,274	0.4045
10	20	0.200	0.025	no	4,462	153.7	144.8	0.7409	0.4143	4,274	0.4141
10	20	0.200	0.050	no	4,462	152.1	144.2	0.7409	0.4283	4,274	0.4282
10	20	0.200	0.100	no	4,462	149.8	143.1	0.7409	0.4051	4,274	0.4050
10	20	0.200	0.200	no	4,462	140.0	141.1	0.7409	0.3539	4,274	0.3538
20	20	0.200	0.010	no	3,951	140.6	145.2	0.6842	0.5005	3,947	0.5004
20	20	0.200	0.025	no	3,951	140.0	145.1	0.6842	0.5111	3,947	0.5110
20	20	0.200	0.050	no	3,951	137.9	144.4	0.6842	0.5276	3,947	0.5275
20	20	0.200	0.100	no	3,951	135.3	143.0	0.6842	0.4972	3,947	0.4971
20	20	0.200	0.200	no	3,951	125.0	140.9	0.6842	0.4334	3,947	0.4332
40	20	0.200	0.010	no	3,228	128.3	145.2	0.5591	0.4098	3,225	0.4096

coverage_floor	merge_window	effect_frac	expr_frac	released	n_genes	mean_five	mean_three	retained	identical	n_nonzero	ident_nonzero
40	20	0.200	0.025	no	3,228	127.1	144.9	0.5591	0.4234	3,225	0.4233
40	20	0.200	0.050	no	3,228	125.1	144.1	0.5591	0.4365	3,225	0.4363
40	20	0.200	0.100	no	3,228	122.3	142.6	0.5591	0.4120	3,225	0.4118
40	20	0.200	0.200	no	3,228	110.6	140.1	0.5591	0.3534	3,225	0.3532
80	20	0.200	0.010	no	2,325	113.1	142.7	0.4028	0.3670	2,323	0.3668
80	20	0.200	0.025	no	2,325	111.6	142.2	0.4028	0.3855	2,323	0.3853
80	20	0.200	0.050	no	2,325	109.1	141.0	0.4028	0.3976	2,323	0.3973
80	20	0.200	0.100	no	2,325	106.4	139.5	0.4028	0.3769	2,323	0.3767
80	20	0.200	0.200	no	2,325	94.5	137.7	0.4028	0.3249	2,323	0.3246
5	40	0.200	0.010	no	5,103	164.6	147.4	0.8231	0.4064	4,748	0.4063
5	40	0.200	0.025	no	5,103	164.1	147.3	0.8231	0.4152	4,748	0.4151
5	40	0.200	0.050	no	5,103	162.8	146.8	0.8231	0.4285	4,748	0.4284
5	40	0.200	0.100	no	5,103	160.8	145.9	0.8231	0.4081	4,748	0.4080
5	40	0.200	0.200	no	5,103	151.8	144.0	0.8231	0.3618	4,748	0.3616
10	40	0.200	0.010	no	4,842	154.7	145.6	0.8055	0.4361	4,647	0.4360
10	40	0.200	0.025	no	4,842	154.2	145.5	0.8055	0.4451	4,647	0.4450
10	40	0.200	0.050	no	4,842	152.8	145.0	0.8055	0.4591	4,647	0.4590
10	40	0.200	0.100	no	4,842	150.6	144.1	0.8055	0.4370	4,647	0.4368
10	40	0.200	0.200	no	4,842	141.4	142.4	0.8055	0.3855	4,647	0.3854
20	40	0.200	0.010	no	4,380	140.1	145.3	0.7591	0.5281	4,379	0.5280
20	40	0.200	0.025	no	4,380	139.5	145.1	0.7591	0.5379	4,379	0.5378
20	40	0.200	0.050	no	4,380	137.7	144.5	0.7591	0.5537	4,379	0.5536
20	40	0.200	0.100	no	4,380	135.3	143.4	0.7591	0.5258	4,379	0.5257
20	40	0.200	0.200	no	4,380	125.8	141.5	0.7591	0.4623	4,379	0.4622
40	40	0.200	0.010	no	3,647	127.2	144.2	0.6321	0.4267	3,646	0.4265
40	40	0.200	0.025	no	3,647	126.2	143.9	0.6321	0.4387	3,646	0.4386
40	40	0.200	0.050	no	3,647	124.6	143.1	0.6321	0.4511	3,646	0.4509
40	40	0.200	0.100	no	3,647	122.0	142.1	0.6321	0.4297	3,646	0.4295
40	40	0.200	0.200	no	3,647	111.3	139.8	0.6321	0.3724	3,646	0.3722
80	40	0.200	0.010	no	2,661	113.0	141.1	0.4612	0.3743	2,660	0.3741
80	40	0.200	0.025	no	2,661	111.7	140.6	0.4612	0.3905	2,660	0.3902
80	40	0.200	0.050	no	2,661	109.7	139.6	0.4612	0.4017	2,660	0.4015
80	40	0.200	0.100	no	2,661	107.3	138.7	0.4612	0.3844	2,660	0.3842
80	40	0.200	0.200	no	2,661	96.6	137.0	0.4612	0.3337	2,660	0.3335
5	80	0.200	0.010	no	5,537	164.0	149.3	0.8993	0.4702	5,188	0.4701
5	80	0.200	0.025	no	5,537	163.6	149.2	0.8993	0.4781	5,188	0.4780
5	80	0.200	0.050	no	5,537	162.4	148.8	0.8993	0.4903	5,188	0.4902
5	80	0.200	0.100	no	5,537	160.4	148.0	0.8993	0.4702	5,188	0.4701
5	80	0.200	0.200	no	5,537	151.4	146.2	0.8993	0.4124	5,188	0.4123

coverage_floor	merge_window	effect_frac	expr_frac	released	n_genes	mean_five	mean_three	retained	identical	n_nonzero	ident_nonzero
10	80	0.200	0.010	no	5,305	154.0	147.6	0.8863	0.4988	5,113	0.4987
10	80	0.200	0.025	no	5,305	153.6	147.5	0.8863	0.5068	5,113	0.5067
10	80	0.200	0.050	no	5,305	152.3	147.1	0.8863	0.5196	5,113	0.5195
10	80	0.200	0.100	no	5,305	150.3	146.4	0.8863	0.4980	5,113	0.4979
10	80	0.200	0.200	no	5,305	141.3	144.7	0.8863	0.4353	5,113	0.4352
20	80	0.200	0.010	no	4,897	140.0	147.0	0.8485	0.5897	4,895	0.5896
20	80	0.200	0.025	no	4,897	139.6	146.9	0.8485	0.5982	4,895	0.5982
20	80	0.200	0.050	no	4,897	138.0	146.3	0.8485	0.6125	4,895	0.6125
20	80	0.200	0.100	no	4,897	135.8	145.4	0.8485	0.5858	4,895	0.5857
20	80	0.200	0.200	no	4,897	126.8	143.7	0.8485	0.5104	4,895	0.5103
40	80	0.200	0.010	no	4,257	125.9	144.1	0.7378	0.4740	4,256	0.4739
40	80	0.200	0.025	no	4,257	125.0	143.8	0.7378	0.4841	4,256	0.4840
40	80	0.200	0.050	no	4,257	123.5	143.2	0.7378	0.4947	4,256	0.4946
40	80	0.200	0.100	no	4,257	121.3	142.3	0.7378	0.4747	4,256	0.4746
40	80	0.200	0.200	no	4,257	111.3	140.3	0.7378	0.4087	4,256	0.4086
80	80	0.200	0.010	no	3,202	110.2	141.1	0.5549	0.4063	3,201	0.4061
80	80	0.200	0.025	no	3,202	109.1	140.7	0.5549	0.4194	3,201	0.4192
80	80	0.200	0.050	no	3,202	107.3	139.8	0.5549	0.4288	3,201	0.4286
80	80	0.200	0.100	no	3,202	105.2	139.1	0.5549	0.4138	3,201	0.4136
80	80	0.200	0.200	no	3,202	95.1	137.7	0.5549	0.3579	3,201	0.3577
5	160	0.200	0.010	no	5,767	168.4	151.8	0.9416	0.6166	5,432	0.6165
5	160	0.200	0.025	no	5,767	168.0	151.7	0.9416	0.6243	5,432	0.6243
5	160	0.200	0.050	no	5,767	166.8	151.3	0.9416	0.6361	5,432	0.6360
5	160	0.200	0.100	no	5,767	164.4	150.5	0.9416	0.6120	5,432	0.6119
5	160	0.200	0.200	no	5,767	153.9	148.8	0.9416	0.5364	5,432	0.5363
10	160	0.200	0.010	no	5,572	159.3	150.0	0.9338	0.6524	5,387	0.6523
10	160	0.200	0.025	no	5,572	158.9	149.9	0.9338	0.6602	5,387	0.6601
10	160	0.200	0.050	no	5,572	157.6	149.5	0.9338	0.6724	5,387	0.6724
10	160	0.200	0.100	no	5,572	155.2	148.8	0.9338	0.6468	5,387	0.6467
10	160	0.200	0.200	no	5,572	144.7	147.2	0.9338	0.5661	5,387	0.5660
20	160	0.200	0.010	no	5,291	145.5	148.5	0.9166	0.7480	5,288	0.7479
20	160	0.200	0.025	no	5,291	145.1	148.4	0.9166	0.7561	5,288	0.7561
20	160	0.200	0.050	no	5,291	143.5	147.9	0.9166	0.7695	5,288	0.7695
20	160	0.200	0.100	no	5,291	141.0	147.1	0.9166	0.7385	5,288	0.7385
20	160	0.200	0.200	no	5,291	130.8	145.5	0.9166	0.6438	5,288	0.6437
40	160	0.200	0.010	no	4,833	128.6	144.6	0.8374	0.6012	4,831	0.6011
40	160	0.200	0.025	no	4,833	127.9	144.4	0.8374	0.6103	4,831	0.6102
40	160	0.200	0.050	no	4,833	126.5	143.9	0.8374	0.6200	4,831	0.6200
40	160	0.200	0.100	no	4,833	124.0	143.1	0.8374	0.5969	4,831	0.5968

coverage_floor	merge_window	effect_frac	expr_frac	released	n_genes	mean_five	mean_three	retained	identical	n_nonzero	ident_nonzero
40	160	0.200	0.200	no	4,833	113.3	141.3	0.8374	0.5174	4,831	0.5173
80	160	0.200	0.010	no	3,952	110.6	138.5	0.6847	0.4903	3,950	0.4901
80	160	0.200	0.025	no	3,952	109.7	138.2	0.6847	0.5014	3,950	0.5013
80	160	0.200	0.050	no	3,952	108.2	137.6	0.6847	0.5092	3,950	0.5091
80	160	0.200	0.100	no	3,952	106.2	137.0	0.6847	0.4920	3,950	0.4919
80	160	0.200	0.200	no	3,952	95.7	135.8	0.6847	0.4272	3,950	0.4271
5	10	0.350	0.010	no	1,231	220.5	174.5	0.1823	0.3660	1,051	0.3654
5	10	0.350	0.025	no	1,231	218.8	174.1	0.1823	0.4011	1,051	0.4006
5	10	0.350	0.050	no	1,231	213.3	172.0	0.1823	0.4468	1,051	0.4462
5	10	0.350	0.100	no	1,231	206.6	169.5	0.1823	0.4002	1,051	0.3996
5	10	0.350	0.200	no	1,231	194.8	160.9	0.1823	0.3460	1,051	0.3454
10	10	0.350	0.010	no	1,051	218.7	176.6	0.1686	0.4193	972	0.4187
10	10	0.350	0.025	no	1,051	216.8	176.1	0.1686	0.4573	972	0.4568
10	10	0.350	0.050	no	1,051	210.2	173.7	0.1686	0.5087	972	0.5082
10	10	0.350	0.100	no	1,051	202.2	171.3	0.1686	0.4543	972	0.4537
10	10	0.350	0.200	no	1,051	188.6	162.4	0.1686	0.3916	972	0.3909
20	10	0.350	0.010	no	845	208.5	186.1	0.1464	0.5527	844	0.5521
20	10	0.350	0.025	no	845	206.1	185.5	0.1464	0.5964	844	0.5960
20	10	0.350	0.050	no	845	197.6	182.2	0.1464	0.6592	844	0.6588
20	10	0.350	0.100	no	845	188.5	179.3	0.1464	0.5870	844	0.5865
20	10	0.350	0.200	no	845	173.1	168.5	0.1464	0.4970	844	0.4964
40	10	0.350	0.010	no	692	193.4	182.1	0.1199	0.4523	691	0.4515
40	10	0.350	0.025	no	692	188.7	181.3	0.1199	0.5072	691	0.5065
40	10	0.350	0.050	no	692	179.9	177.3	0.1199	0.5607	691	0.5601
40	10	0.350	0.100	no	692	169.9	173.4	0.1199	0.4971	691	0.4964
40	10	0.350	0.200	no	692	153.8	162.2	0.1199	0.4263	691	0.4255
80	10	0.350	0.010	no	500	169.1	173.5	0.0867	0.3960	499	0.3948
80	10	0.350	0.025	no	500	163.0	172.4	0.0867	0.4720	499	0.4709
80	10	0.350	0.050	no	500	152.3	167.4	0.0867	0.5220	499	0.5210
80	10	0.350	0.100	no	500	143.1	164.4	0.0867	0.4700	499	0.4689
80	10	0.350	0.200	no	500	132.0	154.5	0.0867	0.4340	499	0.4329
5	20	0.350	0.010	no	1,370	214.9	169.8	0.2043	0.3647	1,178	0.3642
5	20	0.350	0.025	no	1,370	213.4	169.4	0.2043	0.3961	1,178	0.3956
5	20	0.350	0.050	no	1,370	208.7	167.6	0.2043	0.4402	1,178	0.4397
5	20	0.350	0.100	no	1,370	202.5	165.2	0.2043	0.3893	1,178	0.3888
5	20	0.350	0.200	no	1,370	191.2	157.8	0.2043	0.3350	1,178	0.3345
10	20	0.350	0.010	no	1,168	211.4	172.6	0.1877	0.4192	1,082	0.4187
10	20	0.350	0.025	no	1,168	209.6	172.1	0.1877	0.4534	1,082	0.4529
10	20	0.350	0.050	no	1,168	204.1	170.0	0.1877	0.5032	1,082	0.5028

coverage_floor	merge_window	effect_frac	expr_frac	released	n_genes	mean_five	mean_three	retained	identical	n_nonzero	ident_nonzero
10	20	0.350	0.100	no	1,168	196.5	167.6	0.1877	0.4441	1,082	0.4436
10	20	0.350	0.200	no	1,168	183.6	160.0	0.1877	0.3804	1,082	0.3799
20	20	0.350	0.010	no	955	199.7	177.1	0.1655	0.5529	954	0.5524
20	20	0.350	0.025	no	955	197.5	176.6	0.1655	0.5916	954	0.5912
20	20	0.350	0.050	no	955	190.5	174.0	0.1655	0.6513	954	0.6509
20	20	0.350	0.100	no	955	181.8	170.2	0.1655	0.5759	954	0.5755
20	20	0.350	0.200	no	955	166.5	161.0	0.1655	0.4848	954	0.4843
40	20	0.350	0.010	no	783	183.3	174.4	0.1357	0.4432	782	0.4425
40	20	0.350	0.025	no	783	179.1	173.7	0.1357	0.4917	782	0.4910
40	20	0.350	0.050	no	783	171.3	171.1	0.1357	0.5428	782	0.5422
40	20	0.350	0.100	no	783	161.7	166.3	0.1357	0.4802	782	0.4795
40	20	0.350	0.200	no	783	146.3	156.1	0.1357	0.4100	782	0.4092
80	20	0.350	0.010	no	558	163.7	167.7	0.0967	0.3889	557	0.3878
80	20	0.350	0.025	no	558	158.3	166.8	0.0967	0.4588	557	0.4578
80	20	0.350	0.050	no	558	148.6	163.0	0.0967	0.5072	557	0.5063
80	20	0.350	0.100	no	558	139.7	159.6	0.0967	0.4606	557	0.4596
80	20	0.350	0.200	no	558	127.7	149.7	0.0967	0.4176	557	0.4165
5	40	0.350	0.010	no	1,533	206.8	167.6	0.2302	0.3660	1,327	0.3655
5	40	0.350	0.025	no	1,533	205.5	167.3	0.2302	0.3938	1,327	0.3934
5	40	0.350	0.050	no	1,533	201.2	165.7	0.2302	0.4345	1,327	0.4341
5	40	0.350	0.100	no	1,533	195.5	163.4	0.2302	0.3833	1,327	0.3828
5	40	0.350	0.200	no	1,533	184.0	156.7	0.2302	0.3283	1,327	0.3278
10	40	0.350	0.010	no	1,310	201.6	170.8	0.2104	0.4259	1,213	0.4254
10	40	0.350	0.025	no	1,310	200.0	170.4	0.2104	0.4563	1,213	0.4559
10	40	0.350	0.050	no	1,310	195.0	168.6	0.2104	0.5025	1,213	0.5021
10	40	0.350	0.100	no	1,310	188.0	166.3	0.2104	0.4432	1,213	0.4427
10	40	0.350	0.200	no	1,310	175.5	159.4	0.2104	0.3764	1,213	0.3759
20	40	0.350	0.010	no	1,067	191.9	174.9	0.1849	0.5633	1,066	0.5629
20	40	0.350	0.025	no	1,067	189.9	174.5	0.1849	0.5989	1,066	0.5985
20	40	0.350	0.050	no	1,067	183.5	172.2	0.1849	0.6542	1,066	0.6538
20	40	0.350	0.100	no	1,067	175.0	168.6	0.1849	0.5773	1,066	0.5769
20	40	0.350	0.200	no	1,067	160.4	160.4	0.1849	0.4864	1,066	0.4859
40	40	0.350	0.010	no	856	179.0	172.8	0.1484	0.4568	855	0.4561
40	40	0.350	0.025	no	856	175.2	172.2	0.1484	0.5023	855	0.5018
40	40	0.350	0.050	no	856	167.8	169.8	0.1484	0.5502	855	0.5497
40	40	0.350	0.100	no	856	158.7	165.2	0.1484	0.4871	855	0.4865
40	40	0.350	0.200	no	856	142.8	155.8	0.1484	0.4089	855	0.4082
80	40	0.350	0.010	no	608	158.1	165.8	0.1054	0.3964	607	0.3954
80	40	0.350	0.025	no	608	153.1	164.9	0.1054	0.4622	607	0.4613

coverage_floor	merge_window	effect_frac	expr_frac	released	n_genes	mean_five	mean_three	retained	identical	n_nonzero	ident_nonzero
80	40	0.350	0.050	no	608	144.0	161.4	0.1054	0.5082	607	0.5074
80	40	0.350	0.100	no	608	134.7	157.6	0.1054	0.4556	607	0.4547
80	40	0.350	0.200	no	608	121.5	148.3	0.1054	0.4030	607	0.4020
5	80	0.350	0.010	no	1,837	193.4	164.1	0.2794	0.3350	1,611	0.3346
5	80	0.350	0.025	no	1,837	192.3	163.8	0.2794	0.3579	1,611	0.3575
5	80	0.350	0.050	no	1,837	188.7	162.5	0.2794	0.3921	1,611	0.3917
5	80	0.350	0.100	no	1,837	183.6	160.5	0.2794	0.3480	1,611	0.3476
5	80	0.350	0.200	no	1,837	171.7	155.1	0.2794	0.2940	1,611	0.2936
10	80	0.350	0.010	no	1,582	187.9	166.3	0.2553	0.3883	1,472	0.3879
10	80	0.350	0.025	no	1,582	186.6	166.0	0.2553	0.4134	1,472	0.4130
10	80	0.350	0.050	no	1,582	182.5	164.5	0.2553	0.4521	1,472	0.4518
10	80	0.350	0.100	no	1,582	176.4	162.6	0.2553	0.4012	1,472	0.4008
10	80	0.350	0.200	no	1,582	163.4	156.8	0.2553	0.3367	1,472	0.3363
20	80	0.350	0.010	no	1,272	182.7	171.5	0.2205	0.5307	1,271	0.5303
20	80	0.350	0.025	no	1,272	181.0	171.0	0.2205	0.5605	1,271	0.5602
20	80	0.350	0.050	no	1,272	174.9	169.1	0.2205	0.6085	1,271	0.6082
20	80	0.350	0.100	no	1,272	167.4	165.9	0.2205	0.5409	1,271	0.5405
20	80	0.350	0.200	no	1,272	153.0	159.1	0.2205	0.4497	1,271	0.4493
40	80	0.350	0.010	no	1,010	170.3	169.9	0.1750	0.4248	1,009	0.4242
40	80	0.350	0.025	no	1,010	167.1	169.3	0.1750	0.4634	1,009	0.4628
40	80	0.350	0.050	no	1,010	160.8	167.3	0.1750	0.5040	1,009	0.5035
40	80	0.350	0.100	no	1,010	152.5	163.3	0.1750	0.4475	1,009	0.4470
40	80	0.350	0.200	no	1,010	137.0	155.2	0.1750	0.3723	1,009	0.3717
80	80	0.350	0.010	no	710	150.3	162.7	0.1231	0.3620	709	0.3611
80	80	0.350	0.025	no	710	146.0	161.9	0.1231	0.4183	709	0.4175
80	80	0.350	0.050	no	710	138.2	158.9	0.1231	0.4577	709	0.4570
80	80	0.350	0.100	no	710	129.7	155.7	0.1231	0.4099	709	0.4090
80	80	0.350	0.200	no	710	116.0	147.0	0.1231	0.3563	709	0.3554
5	160	0.350	0.010	no	2,747	166.5	155.3	0.4334	0.3075	2,500	0.3072
5	160	0.350	0.025	no	2,747	165.7	155.1	0.4334	0.3231	2,500	0.3228
5	160	0.350	0.050	no	2,747	163.2	154.2	0.4334	0.3447	2,500	0.3444
5	160	0.350	0.100	no	2,747	159.4	152.8	0.4334	0.3127	2,500	0.3124
5	160	0.350	0.200	no	2,747	146.3	149.2	0.4334	0.2631	2,500	0.2628
10	160	0.350	0.010	no	2,460	160.3	155.8	0.4042	0.3478	2,331	0.3475
10	160	0.350	0.025	no	2,460	159.4	155.5	0.4042	0.3645	2,331	0.3642
10	160	0.350	0.050	no	2,460	156.5	154.6	0.4042	0.3881	2,331	0.3878
10	160	0.350	0.100	no	2,460	152.3	153.3	0.4042	0.3525	2,331	0.3522
10	160	0.350	0.200	no	2,460	138.4	149.6	0.4042	0.2937	2,331	0.2934
20	160	0.350	0.010	no	2,017	151.6	159.8	0.3496	0.4650	2,016	0.4648

coverage_floor	merge_window	effect_frac	expr_frac	released	n_genes	mean_five	mean_three	retained	identical	n_nonzero	ident_nonzero
20	160	0.350	0.025	no	2,017	150.5	159.6	0.3496	0.4849	2,016	0.4846
20	160	0.350	0.050	no	2,017	146.4	158.4	0.3496	0.5141	2,016	0.5139
20	160	0.350	0.100	no	2,017	141.3	156.6	0.3496	0.4655	2,016	0.4653
20	160	0.350	0.200	no	2,017	125.8	152.1	0.3496	0.3862	2,016	0.3859
40	160	0.350	0.010	no	1,597	142.1	159.7	0.2768	0.3939	1,596	0.3935
40	160	0.350	0.025	no	1,597	140.0	159.2	0.2768	0.4195	1,596	0.4192
40	160	0.350	0.050	no	1,597	136.0	158.1	0.2768	0.4452	1,596	0.4449
40	160	0.350	0.100	no	1,597	130.3	155.8	0.2768	0.4020	1,596	0.4016
40	160	0.350	0.200	no	1,597	112.6	149.9	0.2768	0.3262	1,596	0.3258
80	160	0.350	0.010	no	1,162	127.8	157.0	0.2014	0.3442	1,161	0.3437
80	160	0.350	0.025	no	1,162	125.1	156.0	0.2014	0.3804	1,161	0.3798
80	160	0.350	0.050	no	1,162	120.3	154.5	0.2014	0.4045	1,161	0.4040
80	160	0.350	0.100	no	1,162	114.1	153.0	0.2014	0.3657	1,161	0.3652
80	160	0.350	0.200	no	1,162	97.6	147.2	0.2014	0.3046	1,161	0.3040

Worst-case range. Across the other 624 (non-released) combinations above, jointly varying all four thresholds by up to four grid steps: retained 0.087–1.000, identical 0.263–0.984, boundaries within 10 nt 0.426–0.988 (released combination: 1.0000 on all three). The least concordant single combination is coverage floor 5, merge window 160, effect fraction 0.35, expressed fraction 0.2 (retained 0.4334, identical 0.2631).

Per-parameter, one step. Holding three thresholds at the released value and moving the fourth by one grid step, the fraction of genes whose boundary call changes at all, and by more than 10 nt, varies sharply by which threshold moves (below); the effect fraction threshold is the most consequential of the four at both ends, the merge window and expressed fraction thresholds close to negligible on their own.

end	parameter moved	frac. of genes moved	frac. moved >10 nt	median move (nt)	max move (nt)
five	min_reads	18.0%	11.1%	0.0	996
five	effect_frac	14.4%	8.9%	0.0	994
five	expr_frac	5.5%	4.0%	0.0	956
five	merge_window	2.6%	1.5%	0.0	854
three	min_reads	8.5%	4.1%	0.0	982
three	effect_frac	7.4%	4.2%	0.0	873
three	merge_window	1.7%	1.1%	0.0	866
three	expr_frac	0.6%	0.5%	0.0	847

Supplementary Table S6. Condition-level variability: NS and S10, pre-merge and post-merge.

Supplementary Table S6. Condition-level variability: NS and S10, pre-merge and post-merge. The two independent physiological conditions behind the six construction libraries, NS and S10, each already the pool of its own two biological replicates, segmented separately by the released method. S6a: the two conditions' own DRS-only calls, before either is merged with Nagalakshmi et al., compared directly with each other (identical, and within 10/20 nt of each other) over the 5,002 genes called under both conditions. S6b: each condition independently merged with Nagalakshmi et al., the same way the release itself is built, and checked against the actual released final annotation. both_agree, only_one_agrees and neither_agrees partition the same 4,999 genes; for the neither_agrees genes, neither_median_best_abs_dev is the distance from the closer of the two conditions' calls to the real call, neither_frac_same_wrong_answer is the fraction where NS and S10 land on the identical (wrong) boundary rather than disagreeing with each other, and the two signed medians give the typical direction each condition misses by. Produced by s16b_reproduction_gap_mechanism.py.

end	n_genes	identical_%	within_10nt_%	within_20nt_%	median_|diff|_nt	mean_|diff|_nt
5′	5,002	21.5	56.1	67.7	8.0	48.4
3′	5,002	42.9	69.5	77.9	2.0	27.8

end	n_genes	both_agree_%	only_one_%	neither_%	neither_n	neither_med_|dev|_nt	neither_within20_%	neither_same_wrong_%	neither_dev_NS_nt	neither_dev_S10_nt
5′	5,002	58.6	21.7	19.6	982	21.0	57.0	37.8	-21.0	-21.0
3′	5,002	73.5	16.7	9.8	489	7.0	79.1	49.7	-7.0	-7.0

Supplementary Table S7. Gene level comparison against the UNAGI annotation, and the two independent benchmarks against each other.

Supplementary Table S7. Gene level comparison against the UNAGI annotation, and the two independent benchmarks against each other. The UNAGI annotation of Al Kadi et al. (2020) is a second boundary set built in a different laboratory from nanopore full-length cDNA, and it took no part in constructing the present reference. Genes were matched on the genomic coordinate of the start or stop codon rather than through an accession map. Signed differences are ours minus UNAGI, so a positive value is a longer UTR here; under-called and over-called mean shorter or longer by at least the stated tolerance, and concordant means an absolute difference below it, the same rule used for TIF-seq. The three rates at each tolerance sum to 100% by construction and are given together so that the direction of every disagreement is visible. Unlike the TIF-seq major isoform, which is a mode, the UNAGI boundary is itself a maximum, so no envelope correction is needed and the rates are already like for like; the block on UNAGI's own dispersion measures how far apart the ends that study observed for a single gene lie, which sets a floor below which a tolerance measures isoform heterogeneity rather than annotation error. The gene counts in that block are the genes whose site list joined to the shared gene model and carry a positive boundary, so they are not the 1,089 and 1,482 quoted in the Results, which are that study's own published counts of genes with alternative 5′ isoforms and with two or more distinct poly(A) sites and are reproduced exactly by the parse. The final block sets the two independent benchmarks against each other on the genes both cover, which calibrates every rate above. Percentages are given to two decimal places as computed; the Results quotes them to one. The per gene join is released as unagi_join.tsv rather than typeset here. Produced by s07i_unagi_compare.py.

Measure	5′ end	3′ end
Coverage of the shared gene model		
Genes with a boundary in this resource	6,005	6,037
Genes with a UNAGI boundary	5,509	5,752
Called by both	5,317	5,537
Called here only	688	500
Called by UNAGI only	192	215
UNAGI UTR length, median (nt)	83	147
UNAGI UTR length, Q1 (nt)	47	101
UNAGI UTR length, Q3 (nt)	194	234
Agreement of the final annotation with UNAGI		
Genes compared	5,317	5,537
Spearman rho	0.46	0.51
Median signed difference, ours minus UNAGI (nt)	-1	2
Q1 to Q3 of the signed difference (nt)	-21 to 48	-14 to 55
Genes concordant within 20 nt, %	39.60	43.70
Genes under-called by at least 20 nt, %	25.70	21.30
Genes over-called by at least 20 nt, %	34.70	35.00
Genes concordant within 50 nt, %	60.10	59.60
Genes under-called by at least 50 nt, %	15.40	14.10
Genes over-called by at least 50 nt, %	24.50	26.30
Genes concordant within 100 nt, %	70.10	73.30
Genes under-called by at least 100 nt, %	11.70	9.60
Genes over-called by at least 100 nt, %	18.20	17.00
Effect of the merge, on the genes both annotations call		
Genes paired	4,536	4,615
Median absolute error, final annotation (nt)	29	30
Median absolute error, prior reference (nt)	28	32

Measure	5′ end	3′ end
Genes moved closer to UNAGI by the merge	761	747
Genes moved further from UNAGI by the merge	525	139
Genes unchanged	3,250	3,729
Wilcoxon signed rank p	0.58	2.6×10^{-80}
Largest provenance class, prior retained, DRS shorter		
Genes in the class	2,884	3,458
Median signed difference (nt)	5	22
Over-called by at least 20 nt, %	38.20	51.60
Under-called by at least 20 nt, %	18.10	12.20
UNAGI's own definition and dispersion		
Genes whose site list joined to the gene model	1,059	2,300
Reported boundary equals the furthest listed site, n	1,055	2,300
Reported boundary equals the furthest listed site, %	99.60	100.00
Alternative sites per gene, median	3	2
Spread between listed sites, median (nt)	23	9
Spread between listed sites, Q1 to Q3 (nt)	10 to 41	0 to 35
Spread of 20 nt or more, %	54.60	36.70
Spread of 50 nt or more, %	19.47	19.13
Spread of 100 nt or more, %	6.02	8.85
The two benchmarks against each other		
Genes covered by both benchmarks	5,160	5,314
Spearman rho, UNAGI against TIF-seq	0.58	0.65
Median absolute difference between benchmarks (nt)	21	14
UNAGI and TIF-seq concordant within 20 nt, %	47.97	56.76
UNAGI and TIF-seq concordant within 50 nt, %	71.34	74.12
UNAGI and TIF-seq concordant within 100 nt, %	81.03	83.91
Genes with a call from all three annotations	5,049	5,187
Ours concordant with UNAGI within 20 nt, %	40.20	43.10
Ours concordant with TIF-seq within 20 nt, %	42.00	45.40
Ours concordant with both within 20 nt, %	27.00	31.90
